# International Society of Sports Nutrition position stand: effects of dietary antioxidants on exercise and sports performance

**DOI:** 10.1080/15502783.2026.2629828

**Published:** 2026-02-17

**Authors:** Drew E. Gonzalez, Broderick L. Dickerson, Brandon M. Roberts, Jennifer A. Kurtz, Hunter S. Waldman, Adam M. Gonzalez, Matthew J. McAllister, Jeffery L. Heileson, Richard J. Bloomer, Shawn M. Arent, Darren G. Candow, Jeffrey R. Stout, Karen A. Hecht, Bill Campbell, Chad M. Kerksick, Douglas Kalman, Jose Antonio, Richard B. Kreider

**Affiliations:** a Exercise & Sport Nutrition Laboratory, Human Clinical Research Facility, Department of Kinesiology & Sport Management, Texas A&M University, College Station, TX, USA; b Occupational, Performance, and Nutrition Laboratory, Department of Kinesiology, Sam Houston State University, Huntsville, TX, USA; c Sargent College of Health & Rehabilitation Sciences, Boston University, Boston, MA, USA; d Department of Kinesiology, Appalachian State University, Boone, NC, USA; e Department of Kinesiology, University of North Alabama, Florence, AL, USA; f Department of Allied Health and Kinesiology, Hofstra University, Hempstead, NY, USA; g Metabolic and Applied Physiology Laboratory, Department of Health & Human Performance, Texas State University, San Marcos, TX, USA; h Nutrition Service Division, Walter Reed National Military Medical Center, Bethesda, Maryland, USA; i College of Health Sciences, University of Memphis, Memphis, TN, USA; j Department of Exercise Science, Arnold School of Public Health, University of South Carolina, Columbia, SC, USA; k Faculty of Kinesiology and Health Studies, University of Regina, Regina, SK, Canada; l School of Kinesiology & Rehabilitation Sciences, University of Central Florida, Orlando, FL, USA; m AstaReal, Inc., Burlington, NJ, USA; n University of South Florida, Performance & Physique Enhancement Laboratory, Tampa, FL, USA; o Exercise and Performance Nutrition Laboratory, Department of Kinesiology, Lindenwood University, St. Charles, MO, USA; p Nova Southeastern University, Department of Nutrition, Dr. Kiran C Patel College of Osteopathic Medicine, Fort Lauderdale, FL, USA; q Nova Southeastern University, Department of Health and Human Performance, Fight Science Lab, Fort Lauderdale, FL, USA

**Keywords:** Oxidative stress, inflammation, adaptation, ergogenic aid

## Abstract

Following a comprehensive review, the International Society of Sports Nutrition (ISSN) has developed an official position on the role of dietary antioxidants in exercise and sport. Antioxidants play a complex, context-dependent role in vivo; they can facilitate recovery from exercise but may also hinder training adaptations when consumed at supraphysiological doses. While endogenous antioxidant systems can effectively maintain redox balance, dietary sources, particularly whole foods, can help mitigate excessive oxidative stress following intense/heavy training or inadequate recovery. The influence of dietary antioxidants depend on timing, dosage, type, and individual factors. The ISSN’s official position encompasses the following: (1) Redox balance exists on a spectrum, with mild oxidative eustress driving beneficial physiological adaptations and excessive oxidative distress impairing health, recovery, and performance; (2) Moderate levels of exercise-induced reactive oxygen and nitrogen species (ROS/RNS) can support training adaptations but excessive levels can result in muscle damage, inflammation, and reduced physical performance and immune function; (3) Endogenous and exogenous antioxidants protect cells by neutralizing free radicals and reducing oxidative damage to biomolecules; (4) FDA labeling for “antioxidant” claims applies to nutrients with established RDIs and demonstrated antioxidant activity; this typically includes vitamins C and E, β-carotene (a source of vitamin A), selenium, zinc, copper, and manganese; (5) While dietary antioxidants show potential for both direct and indirect effects, the evidence varies, and their use should be tailored to individual performance or recovery goals; (6) Long-term exercise augments endogenous antioxidant defense and should be the primary strategy for enhancing redox capacity before considering supplementation; (7) Whole foods and beverages rich in flavonoids, polyphenols, carotenoids, vitamins, and minerals are preferred antioxidant sources; (8) Dietary supplementation is best reserved for nutrient insufficiencies or deficiencies, inadequate dietary intake, or periods of high training distress; (9) Responses to supplementation vary by individual factors, such as training status, baseline antioxidant capacity, demographics, diet, and injury risk, with some antioxidant compounds offering cognitive, behavioral, or physical-related benefits; and (10) Creatine monohydrate (i.e. 0.1 g/kg/day), omega-3 fatty acids (1000–6000 mg/day EPA+DHA for 6–12 weeks), tart cherry (480 mg powder or 60–90 mL juice/day for 7–14 days), and astaxanthin (4–12 mg/day for 4–12 weeks) rank among the top nutrients for their antioxidant effects, with moderate- to high-quality evidence supporting their use in recovery or performance without interfering with training adaptations. Most others show weak or low efficacy. This position promotes an individualized, evidence-based approach, recognizing that small to moderate exercise-induced oxidative stress aids adaptation, while excess oxidative stress causes harm; it also emphasizes food-forward and dietary supplementation strategies.

## 1 Introduction

Athletes and human performance professionals (e.g. strength and conditioning coaches, sports dietitians, and tactical facilitators) frequently seek strategies to gain a competitive edge. These individuals often turn to dietary supplements to enhance exercise, athletic, or occupational performance and recovery. Accordingly, dietary or exogenous antioxidants have been proposed as ergogenic aids because of their ability to neutralize free radicals and exhibit anti-inflammatory properties [1–4].

**Figure 1.**
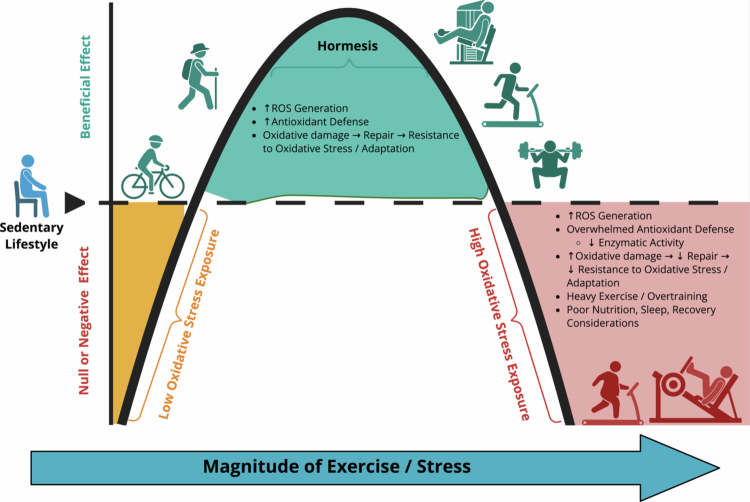
Exercise-induced oxidative stress and the hormetic response. Conceptual model illustrating the dose–response relationship between the magnitude of exercise-induced stress and its physiological effects, with a specific focus on redox balance and adaptation. The x-axis represents increasing magnitude of exercise or stress exposure, while the y-axis reflects the net biological effect, ranging from null or negative to beneficial. At very low levels of stress (e.g. sedentary behavior or minimal physical activity), insufficient reactive oxygen species (ROS) signaling results in limited antioxidant defense activation and minimal adaptive stimulus. Moderate increases in exercise stress fall within the hormetic zone, characterized by transient elevations in ROS that stimulate endogenous antioxidant defenses, enhance cellular repair mechanisms, and promote resistance to oxidative stress, thereby supporting favorable physiological adaptation. In contrast, excessive or prolonged stress exposure – such as high training loads, overreaching or overtraining, inadequate recovery, poor sleep or nutrition, or adverse environmental conditions (e.g. altitude or air pollution) – leads to excessive ROS generation, overwhelmed antioxidant capacity, impaired repair processes, and diminished adaptive responses. Collectively, the figure highlights the nonlinear, inverted-U relationship between exercise stress and biological outcomes, emphasizing the importance of appropriate training loads and recovery to maximize adaptation while minimizing maladaptation. Abbreviation: ROS, reactive oxygen species.

Oxygen consumption increases during exercise [5] and contributes to the production of free radicals, such as reactive oxygen species (ROS) and reactive nitrogen species (RNS), whereas prolonged and/or heavy exercise training acutely augments this production [6]. Exercise-induced oxidative stress (EIOS) appears to exhibit both positive and negative effects, with moderate increases in ROS/RNS levels promoting favorable physiological adaptations, while excessive levels can damage cells and contribute to the development or progression of fatigue and/or various disease states (Figure 1) [7].

Exogenous antioxidants can influence EIOS, and some evidence suggests that they may attenuate the ROS-mediated pathways necessary for training adaptations [7]. Current evidence supports supplementation, primarily when correcting an insufficiency or deficiency, meeting dietary needs that cannot be met through food, or during periods of elevated stress, such as overreaching, back-to-back competitions, or in austere environments (e.g. smog and altitude) [1,4,8–10]. One’s age, sex, training status, dietary habits, and genetic polymorphisms, as well as factors such as redox biology and exercise type, may influence individual responses to supplementation, making the selection of an appropriate dietary antioxidant challenging [1,11]. Therefore, decisions regarding the use of exogenous antioxidants must be carefully considered.

This document presents the International Society of Sports Nutrition’s (ISSN) position on the effects of dietary antioxidants on exercise and sports performance, with a focus on EIOS and training adaptations, as well as recovery and performance parameters. The key objectives of this ISSN position stand are to (1) provide an overview of the mechanisms of dietary antioxidants, (2) discuss the scientific literature regarding dietary antioxidants and exercise/sports performance and recovery, and (3) discuss whether resistance-trained and endurance athletes should consider supplementing with antioxidants. Based on the available evidence, the dietary antioxidants discussed in this position stand are rated by the authors as having weak, moderate, or high levels of support for improving exercise- and sport-related outcomes.

## 2 Methods

ISSN position stands are invited papers that the ISSN Editors and Research Committee identify as being of interest to our readers who need position stands to guide evidence-based practice. Editors and/or the Research Committee identify a lead author or team of authors to perform a comprehensive literature review. The draft is then sent to leading scholars for review and comment. The paper is then revised into a consensus statement, reviewed, and approved by the Research Committee and Editors as the official position of the ISSN. This paper also provides a classification of the included dietary antioxidants, ranking them according to the strength of available evidence regarding their effects on exercise performance and antioxidant-related outcomes. Consistent with the 2018 ISSN position stand on research and recommendations [12], the primary writing team classified dietary antioxidants across both the *performance* and *antioxidant* domains using the following evidence-based criteria:

(1) **High** – *Strong evidence to support efficacy and apparently safe*: Supplements backed by solid theory and supported by most well-controlled studies in relevant populations, using proper dosing regimens, demonstrating both effectiveness and safety.
(2) **Moderate** – *Limited or mixed evidence to support efficacy*: Supplements with a solid scientific basis but inconsistent results in the existing research. These supplements need more high-quality studies to clarify their effects; however, there is currently no evidence indicating that they are unsafe or harmful when used properly.
(3) **Low** – *Little to no evidence to support efficacy and/or safety*: Supplements that generally lack a solid scientific basis and/or for which the available evidence consistently shows no effectiveness. Supplements with documented safety issues or potential risks are also included in this category.

Notably, these classifications represent a continuum of scientific evidence rather than rigid categories. Accordingly, some supplements may fall between classifications (e.g. low-to-moderate or moderate-to-high), depending on the consistency, quality, and relevance of the available data.

## 3 What is an antioxidant?

An antioxidant refers to any substance, whether endogenous or exogenous, that reduces or prevents oxidation, defined as the loss of electrons or the addition of oxygen to lipids, carbohydrates, proteins, and deoxyribonucleic acid (DNA) [13]. The antioxidant defense system comprises endogenous enzymatic (i.e. superoxide dismutase [SOD], catalase [CAT], and glutathione peroxidase [GPx]) and non-enzymatic (e.g. glutathione [GSH], uric acid, and coenzyme Q10 [CoQ10]) antioxidants, in addition to exogenous/dietary antioxidants from food and supplemental sources (e.g. tart cherry, blueberry, and vitamin E). To be included in the academic definition of an antioxidant, the compound must exhibit antioxidant activity.

Our definition differs from the FDA 21 Code of Federal Regulations (CFR) § 101.54(g), which permits the “antioxidant” label only for nutrients that (1) demonstrate, after absorption, the ability to inactivate free radicals or prevent free radical-initiated reactions; (2) have an established reference daily intake (RDI); (3) meet defined minimum content levels (e.g. ≥20% of the RDI per serving for a “high” claim); and (4) are specifically identified on the label. According to these requirements, only vitamins C, E, and A (β-carotene), selenium, zinc, copper, and manganese are recognized by the FDA as dietary antioxidants. Other bioactive compounds, such as creatine, CoQ10, N-acetylcysteine, resveratrol, and quercetin, exhibit direct or indirect antioxidant effects but lack an RDI and thus are not FDA-recognized; however, these compounds are included in this analysis.

## 4 Mechanisms of action

Antioxidants can be derived from a wide variety of sources to mitigate oxidative damage to cellular components [14]. Antioxidants are considered reducing agents that neutralize or scavenge pro-oxidants (i.e. ROS, RNS, and free radicals) by several mechanisms, such as electron donation or quenching singlet oxygen. Within the context of health and disease states, antioxidants have the following mechanisms of action: (1) act as agents to catalytically eliminate ROS (i.e. SOD enzymes); (2) act as agents that minimize the availability of potent catalysts, such as iron ions, copper ions, heme, and heme-containing proteins, thus preventing or reducing the opportunity for oxidative damage to these metal ions; (3) act as singlet oxygen (¹O₂) quenchers, such as carotenoids, which can remove excess energy from singlet oxygen and return this molecule to normal oxygen; (4) act as “sacrificial agents,” which preferentially interact with ROS, halting their ability to damage “more important” biomolecules; and (5) undergo structural sequestration or compartmentalization, wherein the physical containment or breakdown of damaged cellular components prevents further propagation of oxidative injury [15]. In addition to direct mechanisms, many dietary and supplemental compounds function as indirect antioxidants by activating protective pathways, such as the Keap1/Nrf2/ARE system. These pathways can enhance phase II detoxification and the activity of antioxidant enzymes that remain active during redox reactions [16,17]. Taken together, these are the mechanisms of action by which antioxidants mitigate oxidative damage during exercise or sports.

## 5 Redox balance and oxidative stress

To understand the application of dietary antioxidants, it is essential to understand the concepts of redox balance, the distinction between oxidative eustress and stress, and EIOS. The following sections provide a brief review of these concepts. For further detail, see reviews by Helmut Sies [18–20] and Scott Powers [7,21].

### 5.1 Redox balance and oxidative stress

Helmut Sies defined “oxidative stress” as a disturbance in the balance of pro-oxidants and antioxidants, favoring pro-oxidants [18]. Since then, Sies’s definition has been adapted to be more comprehensive – “an imbalance between oxidants and antioxidants in favor of the oxidants, leading to a disruption of redox signaling and control, and/or molecular damage [7,19,22].” It is essential to appreciate this definition, given the complex cellular redox dynamics that occur *in vivo*. As the fields of redox biology and medicine have advanced, our understanding of oxidative stress and redox balance has also evolved. It is proposed that a steady-state redox setpoint exists, and any deviation from this setpoint results in the redox balance being in a state of stress [19]. Sies and colleagues [20] recognized that there are both states of oxidative stress (technically *oxidative distress*) and oxidative eustress, with the latter referring to an essential aspect of redox control and physiological redox signaling that supports health and adaptation. An important nuance here is the difference between oxidative stress and oxidative damage. Oxidative stress can be reversible, resulting in temporary redox imbalance or perturbed signaling, whereas oxidative damage is typically irreversible, leading to downstream structural alterations in biomolecules [23]. This distinction is essential for understanding biomarker data in exercise and recovery, as studies may report that biomarkers indicate oxidative damage rather than temporary oxidative stress.

### 5.2 Oxidative stress and eustress: ROS, RNS, and free radicals

Free radicals are molecules with unpaired electrons that are highly reactive in chemical reactions with other molecules [20]. These radicals can be categorized depending on whether they are derived from oxygen or nitrogen (i.e. ROS or RNS). The term “ROS” encompasses the following: singlet oxygen (¹O₂), superoxide (O_2_^·–^; the parent ROS molecule), hydrogen peroxide (H_2_O_2_; formed from the dismutation of O_2_^·–^), the hydroxyl radical (·OH, formed from reactions involving H_2_O_2_ and O_2_^·–^), and peroxyl radicals (ROO·). Notably, ·OH poses a high risk of oxidative damage due to its high reactivity. In terms of RNS, nitric oxide (NO) is considered the parent RNS and can react with O_2_^·–^ to form peroxynitrite (ONOO^−^), which is a reaction that can occur three times faster than the dismutation of O_2_^·–^, suggesting that the formation of ONOO^–^ is highly likely to occur when O_2_^·–^ is present. Consequently, the formation of ONOO^–^ reduces the amount of available NO. Additionally, because ONOO^−^ is a strong oxidizing agent, it can damage thiol groups and nitrate cellular proteins. Ultimately, these ROS/RNS can react with other radicals or cellular components, resulting in oxidative damage. Whether an individual can resolve an oxidative challenge and return to redox balance determines whether the free radicals impart distress or eustress. A state of oxidative distress can negatively impact an individual's health and performance if it remains chronically elevated [7].

### 5.3 The paradoxical nature of EIOS

Originally, exercise-induced ROS/RNS were thought to be purely detrimental to muscle function and performance [7,21,24]. However, the hormesis model describes a biphasic response in which low to moderate exposure to ROS/RNS stimulates the adaptive upregulation of endogenous antioxidants, thereby improving cellular function. In contrast, chronic or excessive exposure to ROS/RNS causes oxidative damage [25–28]. This bell-shaped response means that too few ROS fails to trigger beneficial adaptations. Moreover, excessive ROS/RNS, which can result from overtraining, inadequate recovery, or under-fueling, can lead to inflammation, muscle damage, and immunosuppression [28]. Although there is no known optimal dose of exercise-induced ROS/RNS for each individual, applying low to moderate training intensity with sufficient recovery generally promotes favorable redox signaling, while excessive stress impairs performance and health [7].

### 5.4 Oxidative stress, exercise, and chronic disease risk

Numerous diseases are thought to be linked to chronic exposure to oxidative stress, either as a cause or a result of the disease [29]. This phenomenon is interesting because exercise, which is linked to reduced susceptibility to chronic diseases and all-cause mortality [30,31], can also trigger the production of ROS/RNS. However, this highlights the concept of eustress, as exercise promotes favorable adaptations [32] and upregulates the endogenous antioxidant defense system [21,33,34]. Powers and colleagues [7] concluded that EIOS is a “friend” rather than a “foe,” given the substantial body of evidence that demonstrates exercise is linked to a reduction in all-cause mortality. In terms of supplementation with dietary antioxidants, athletes, including tactical athletes and master-level athletes or elderly individuals, may consider leveraging these compounds for health-related outcomes that extend beyond the exercise- and sport-specific contexts. However, dietary antioxidant intake may introduce competing goals. On the one hand, some antioxidants may interfere with exercise-induced adaptations through hormesis. On the other hand, daily antioxidant intake may help maintain redox balance and confer benefits. This raises questions about the considerations at play when working with “special populations,” that are influenced by multitude of factors affecting their health and physical performance (*see Section 12 on Special Considerations*).

## 6 Oxidative stress during exercise

### 6.1 Exercise and free radical production

Exercise causes the production of ROS/RNS. Early work by Dillard and colleagues [35] demonstrated that a 60-min cycle ergometer exercise bout at 50% V̇O_2_max resulted in increased expired pentane, an index of lipid peroxidation. Approximately one decade later, Lovlin et al. [36] and Gohil et al. [37] reported evidence that cycling exercise bouts triggered oxidative stress, as shown by increased concentrations of the lipid peroxidation marker thiobarbituric acid reactive substances (TBARS) and decreased GSH levels accompanied by increased oxidized GSH (GSSG). Several other research teams reported similar effects of acute aerobic exercise on the production of oxidative stress biomarkers [6,7,21,34,38,39]. Then, reports on the impact of anaerobic exercise emerged, as seen in Marzatico et al. [40], who demonstrated that six sets of 150-m sprints increased blood malondialdehyde (MDA) concentrations, another marker of lipid peroxidation, in sprint-trained athletes but not in marathon runners. McBride and colleagues [41] found that performing three sets of eight resistance exercises at a 10-repetition maximum load resulted in increased blood MDA immediately post-exercise. Importantly, these studies [35–37,40,41], among others [42–44], evaluated blood oxidative stress biomarkers, which is the most common method used to analyze EIOS. Other reports have also demonstrated that resistance exercise training can increase oxidative stress biomarkers via muscle biopsies, saliva, and urine [45–47].

### 6.2 Sources of free radical production during exercise

The discovery of EIOS has led researchers to identify possible sites of ROS/RNS generation within and around skeletal muscle. Interestingly, while it was initially believed that mitochondria were the primary source of ROS generation, it has become increasingly apparent that this is not the case. Boveris and colleagues [48] demonstrated that approximately 2%–5% of the molecular oxygen consumed by mitochondria produces O_2_^·–^, which was thought to increase only with the metabolic demands of exercise (i.e. increased oxidative phosphorylation in contracting skeletal muscle). However, several other sites of ROS/RNS generation contribute more substantially to EIOS, which include phospholipase A_2_ (PLA_2_) and NADPH oxidases [49]. It has been postulated that PLA_2_ contributes to ROS/RNS production through several mechanisms, such as (1) the activation of NADPH oxidases via signaling intermediates that facilitate enzyme assembly, (2) the release of arachidonic acid, which, when metabolized by enzymes, leads to the formation of ROS and RNS byproducts (e.g. O_2_^·–^, H_2_O_2_, ONOO^−^), and (3) the stimulation of mitochondrial ROS production via a calcium (Ca^2+^)-dependent isoform of PLA_2_ (i.e. iPLA_2_ or cPLA_2_). During exercise, when the mitochondrial membrane is disrupted by PLA_2_, leakage of electrons from complexes I and III of the electron transport chain can generate ROS [50]. Powers et al. [7] concluded that more research is warranted to confirm this postulation.

In terms of NADPH oxidase, there are two isoforms within skeletal muscle: NOX1, NOX2, and NOX4 [51]. The NOX2 isoform is found in the sarcolemma and T-tubule, while the NOX4 is found in the sarcoplasmic reticulum and mitochondria [52]. Importantly, NOX2 appears to be the primary site of NADPH-mediated ROS production in exercising muscle, as it is activated by agonists such as cytokines and angiotensin II, as well as by contractile or mechanical stress. Upon mechanical stress or contraction of skeletal muscle, intracellular signaling (e.g. increased calcium or ROS) can promote the translocation of cytosolic subunits to the myocyte membrane, forming an active complex in which NADPH transfers electrons to oxygen molecules to generate O_2_^·–^. Given its mechano- and chemo-sensitivity, NOX2-mediated ROS production increases progressively with the onset and intensity of exercise. Importantly, NOX isoforms have been identified as the primary source of O_2_^·–^ production during rest and exercise [7].

Xanthine oxidation is another implicated source of ROS production in skeletal muscle. The enzyme xanthine oxidase (XO) is located within the capillary endothelial cells that surround skeletal muscle, and muscle contraction can trigger a reaction that activates XO to generate O_2_^·–^, which, when converted to H_2_O_2_ by extracellular SOD, can cross the sarcolemma to enter the myocyte and cause further pro-oxidative effects. XO-mediated O_2_^·–^ production can also interact with NO, produced by skeletal muscle-expressed isoforms of nitric oxide synthase (NOS; nNOS and eNOS) to form ONOO^–^ [53]. The proximity of key sites of ROS/RNS production to skeletal muscle aligns with evidence showing that localized EIOS serves as a proximal signal driving adaptive responses [54].

## 7 Effects of exercise-induced oxidative stress on performance parameters

Previous reports suggest a connection between oxidative stress, fatigue, and muscle damage, which can impact exercise performance [55–57]. Interestingly, a link exists between increased oxidative stress biomarkers and performance or recovery. For example, Stein and colleagues [58] reported that US Special Forces members who were not selected in the SFAS course had higher levels of metabolites associated with increased oxidative stress. These levels could be due to factors such as poor nutrition, non-functional overreaching or overtraining, or incomplete recovery from prior physical exertion. Furthermore, those selected individuals demonstrated resistance to oxidative stress and exhibited greater physical performance than non-selected individuals [58]. While this study is specific to military selection [58], the concept has been described elsewhere [7,34,59–63]. Additionally, the inflammatory response that occurs during and after exercise mobilizes and activates the immune system to respond to muscle damage. This immune response is further stimulated by stress hormones (i.e. catecholamines, growth hormone, and cortisol), which are released in response to increasing metabolic demands during exercise [64,65]. These physiological responses to exercise can influence subsequent training or competition bouts.

### 7.1 Force production, muscle fatigue, and recovery

Force production, defined as the capacity of an individual muscle or group of muscles to generate contractile tension, is a critical element of exercise and sports performance. As such, Reid et al. [66] developed a theoretical model describing the association between skeletal muscle redox homeostasis and force production. In short, Reid’s model resembles the exercise-induced hormesis model [26,27,67], suggesting that, under normal physiological conditions, ROS/RNS production plays a crucial role in adapting to exercise [6]. In contrast, excessive production can lead to oxidative damage to the structural integrity of skeletal muscle proteins involved in muscle contraction [39,68]. Specifically, ROS/RNS can influence skeletal muscle contraction through (1) the modulation of Ca^2+^ handling/excitation‒contraction coupling, (2) direct oxidation of contractile proteins, (3) neuromuscular transmission (i.e. impairment of acetylcholine release and receptor sensitivity at the neuromuscular junction site), and (4) nitrosative stress (i.e. excessive NO production leading to ONOO^−^, which in turn can cause protein nitration). Ultimately, these effects can lead to reduced cross-bridge formation, impaired muscle fiber recruitment, and a diminished ability to sustain muscle contractions, thereby inhibiting force output and muscular endurance. Another important aspect of this cascade of events is that ROS/RNS production can promote inflammation via the nuclear factor kappa B (NF-κB) redox-sensitive transcription factor. For example, H_2_O_2_ or ONOO^–^ can phosphorylate and degrade the inhibitor of κB (IκB), thereby releasing NF-κB, which can translocate to the nucleus and activate the transcription of pro-inflammatory cytokines. These cytokines can further reduce force output through similar mechanisms (i.e. impaired excitation‒contraction coupling) or proteolytic signaling, promoting greater fatigue and slower recovery. Under conditions of high-intensity, prolonged, or fatiguing exercise, ROS/RNS production may overwhelm the endogenous antioxidant capacity, leading to oxidative stress, oxidative damage, muscle fatigue and damage, and even delayed onset muscle soreness (DOMS) [69,70]. However, importantly, at low-to-moderate levels of ROS/RNS production, these radicals can facilitate Ca^2+^ release (short-term force production influence) and trigger signaling pathways for adaptation for a more long-term influence on force production (i.e. upregulation of peroxisome proliferator-activated receptor-γ coactivator 1-α [PGC-1α] for mitochondrial biogenesis).

Current evidence suggests that EIOS is likely to affect recovery between training sessions or competitions, primarily due to its association with fatigue, muscle damage, and impaired immune function [49,71]. The post-exercise recovery phase is characterized by oxidative stress stemming from the infiltration and activation of immune cells, such as neutrophils and macrophages, into damaged skeletal muscle tissue [34,72,73]. While these immune cells play a key role in the normal inflammatory and tissue repair response, they may also produce excessive ROS via NOX2 activation. In addition, ROS levels can trigger temporary, reversible, and irreversible oxidative modifications in skeletal muscle, including S-nitrosylation, S-glutathionylation, and disulfide bond formation [74–76]. Specifically, S-glutathionylation of cysteine residues can lead to protein deactivation and inhibition of activity via extracellular vesicles [74]. Therefore, this kind of redox alteration after translation could play a significant role in skeletal muscle signaling during exercise and recovery [77,78]. One additional area that links EIOS to perceived recovery among athletes is DOMS, which often occurs after extreme or unaccustomed exercise [69]. It has been shown that ROS are involved in DOMS, with inflammatory agents in the muscle identified as the source of production [79]. Early studies revealed a connection between ROS, lipid peroxidation, and DOMS after a downhill running protocol was used [79,80]. The timing between ROS production and peak DOMS seems to depend on the context, with some studies showing that ROS levels increase after peak soreness, suggesting that they are out of sync [79]. Conversely, other evidence suggests that ROS production and inflammation may occur closer to the onset of DOMS, especially in untrained people performing unfamiliar eccentric exercise [79,81]. An individual's training history and exercise background probably influence the timing and intensity of oxidative and inflammatory responses after muscle damage [44,69,82–86]. Indeed, adaptations associated with chronic training may attenuate or delay oxidative stress and inflammatory signaling, potentially altering the temporal relationship between ROS production, neutrophil infiltration, and perceived muscle soreness compared with untrained individuals.

Numerous studies have shown that training leads to adaptations in the antioxidant system, thereby reducing oxidative stress. However, evidence suggests that intense exercise can trigger an increase in oxidative stress [87,88], which may contribute to the onset of overtraining and potentially increase oxidative stress [89–91]. Athletes who display overtraining syndrome may be in a state of chronic oxidative stress, which is likely to be indicated by elevated blood biomarkers or reduced plasma antioxidant capacity [92]. These findings suggest that oxidative stress is not only a contributor to overtraining but also a symptom of overtraining. The concept of using an antioxidant to accelerate recovery is intriguing because it could lead to improvements in athletic performance or an increase in the volume of training one can perform. A faster recovery could enable athletes to increase their training frequency or intensity, thereby enhancing their overall performance and competitive edge. However, it is also important to consider this in the context of chronic adaptation.

### 7.2 Muscular adaptation

It has been clearly demonstrated that the contraction of skeletal muscles will generate ROS/RNS and that low-to-moderate levels of ROS/RNS production can facilitate adaptation in the long term as a means to improve the muscle’s ability to handle future exposure to stressors (i.e. subsequent bouts of training or competition) [7,21]. The type, volume, duration, and intensity of the stressor can dictate the balance between beneficial and detrimental outcomes and whether it creates a state of oxidative distress (commonly referred to as oxidative stress) or eustress. In the context of oxidative eustress (i.e. a modest increase in ROS/RNS), the (1) activation of redox-sensitive transcription factors (i.e. NF-κB) [93], (2) promotion of mitochondrial biogenesis via PGC-1α [94], (3) enhancement of the endogenous antioxidant defense system (e.g. upregulation of endogenous antioxidants, such as catalase) [33,95], and (4) facilitation of muscle remodeling and hypertrophy via the modulation of mitogen-activated protein kinase (MAPK) signaling (i.e. p38 and ERK1/2) [94,96] are all potential ways in which skeletal muscle can adapt to better handle stressors in the future encounter (i.e. subsequent training/competition). Furthermore, preserving redox homeostasis, which involves maintaining ROS/RNS at non-harmful levels while still allowing them to function as signaling molecules, is crucial for a cell to mitigate its toxic effects [97]. As such, mature skeletal muscle cells, along with myogenic stem and progenitor cells, possess antioxidant systems that allow them to adapt to changes in the redox environment [98]. Oxidative stress can also alter the contractility of striated muscle cells under both physiological and pathological conditions, with modifications to the titin protein, directly and indirectly, affecting muscle elasticity and stiffness through changes in protein kinase signaling pathways and protease activation [98]. In contrast, when the stressor creates a state of oxidative stress characterized by excessive ROS/RNS levels, muscle contractile function, inhibition of muscle protein synthesis, mitochondrial damage, and suppression of satellite cell activity are likely impaired, which can ultimately hinder muscle repair and growth, as well as exercise performance [97,99,100].

### 7.3 Endurance performance

High-intensity or prolonged endurance training increases free radical production and endogenous antioxidant defense. Previous reports have shown that endurance training increases SOD1 and SOD2 activity in skeletal muscle by 20%–112%, while increasing GPx activity by 20%– 177% [6,34,101]. Work by Berzosa et al. [102] and Georgakouli et al. [103] demonstrated that a 30-min submaximal cycle ergometer exercise bout at 70% maximum workload and 50%–60% of heart rate reserve increased plasma total antioxidant capacity (TAC). The upregulation of endogenous antioxidant capacity in response to elevated oxidative stress is widely considered a key mechanism underlying endurance-related adaptations (i.e. mitochondrial biogenesis). However, prolonged or high-volume endurance training may have drawbacks, including temporary decreases in antioxidant capacity [4]. These decreases affect not only endogenous antioxidant enzymes but also circulating dietary antioxidants, such as vitamin E, during intense training periods, further impacting overall antioxidant levels [104]. This increased oxidative stress can lead to immune suppression, potentially impairing recovery and increasing the risk of illness and overtraining [59,92,105–108]. One proposed mechanism is that leukocyte-derived ROS and RNS, which are critical for immune defense, become dysregulated or suppressed with excessive training loads, potentially reducing the body’s ability to fight infections [109]. This effect has been demonstrated in athletes competing in ultra-endurance events, including ultramarathons and Ironman triathlons [110–112]. While concerns about cumulative oxidative damage persist, some researchers argue that current evidence does not definitively demonstrate that high-intensity endurance training causes lasting oxidative damage in well-adapted or trained athletes [113].

**Takeaway summary:** EIOS can have either beneficial or detrimental effects on force production, recovery, muscle adaptations, and endurance-related performance outcomes, depending on the level of ROS/RNS production from exercise or training bout. Nevertheless, it is recommended to consider the training goals (recovery versus adaptation) and phase of training or competition (i.e. in-season or off-season) concerning these outcome variables to ensure that (1) the level of EIOS is appropriate to generate a favorable impact on the individual and their performance and/or health, and (2) any interventions with dietary antioxidants are not blunting key adaptations and normal physiological responses necessary for improvement through training.

## 8 How do antioxidants combat oxidative damage and inflammation

Three main categories of antioxidants meet the definition of substances that can slow the oxidation of proteins, lipids, carbohydrates, and DNA. Two key endogenous antioxidant enzymes are SOD and catalase. SOD converts O_2_^·–^ (a highly reactive free radical) to H_2_O_2_ (a less reactive free radical), which can then be further broken down by catalase to yield water (H_2_O) and oxygen (O_2_). This reaction prevents the formation of ·OH, which is considered the most reactive free radical. Second, non-enzymatic antioxidants, such as uric acid and GSH, can scavenge ROS/RNS and donate electrons to highly reactive free radicals, converting them to more stable, less reactive species. For instance, uric acid can react with ·OH to form allantoin and H_2_O or neutralize ONOO^–^ to prevent protein nitration. Similarly, GSH can donate electrons to ·OH or H_2_O_2_, among other free radicals, to convert them to more stable and less reactive species. Finally, functional enzymes (i.e. proteases, lipases, etc.) not only modulate the general metabolism of macromolecules but also exhibit indirect roles in mitigating oxidative damage via multiple mechanisms (i.e. autophagy, blunting misfolded protein translation, mobilizing stored lipids, reducing lipid accumulation, etc.). Dietary antioxidants are generally considered an extension of the non-enzymatic antioxidants mentioned above (Table 1).

**Table 1.** Classes of antioxidants.

Antioxidant class	Key examples/subclasses	Common food sources	Solubility	Structural characteristics	Antioxidant mechanisms
Flavonoids	Flavonols, flavones, flavanones, flavanols, isoflavones	Fruits, vegetables, herbs, cereals, nuts, seeds, stems, flowers	Water-soluble	Benzopyran backbone (C6-C3-C6); phenolic or polyphenolic groups	Hydrogen/electron donation from hydroxyl (–OH) groups to neutralize free radicals (e.g. ROO·, ·OH) Metal chelation (e.g. Fe²⁺) via 3’,4’-dihydroxy B-ring or 3-OH/4-keto on C-ring Induction of Nrf2 signaling
Anthocyanins (Flavonoid subclass)	Cyanidin, delphinidin, malvidin	Berries, red/purple grapes, red cabbage, eggplant skin	Water-soluble	Flavylium ion; pH-dependent color shifts; C6-C3-C6 skeleton	Hydrogen/electron donation from –OH groups Transition metal chelation Support of endogenous defense via Nrf2
Polyphenols (Non-flavonoid)	Phenolic acids, stilbenes (e.g. resveratrol), lignans	Tea, coffee, wine, whole grains, seeds, legumes	Mostly water-soluble	Multiple phenol rings; varied backbones	Direct ROS/RNS scavenging Modulation of signaling pathways like Nrf2
Tocopherols and Tocotrienols	α-, β-, γ-, δ-tocopherol	Vegetable oils, nuts, seeds, green leafy vegetables	Lipid-soluble	Chromanol ring + phytyl tail (tocopherol); unsaturated tail (tocotrienol)	Lipid peroxyl radical scavenging (ROO·) in cell membranes Chain-breaking antioxidant in lipid environments
Carotenoids	Astaxanthin, β-carotene, lutein, lycopene, zeaxanthin	Carrots, tomatoes, sweet potatoes, spinach, kale	Lipid-soluble	Long conjugated double-bond system (polyene chain)	Quenching of singlet oxygen (¹O₂) Scavenging peroxyl and other radicals Indirect modulation of gene expression
Trace Elements (Cofactor-based antioxidants)	Selenium, Zinc, Copper, Manganese	Brazil nuts, seafood, meat, whole grains, legumes	Varies (bioavailable as ions or chelated forms)	Inorganic elements, often bound in metalloenzymes (e.g. GPx, SOD)	Cofactors for endogenous antioxidant enzymes (e.g. Glutathione Peroxidase, Superoxide Dismutase) Facilitate neutralization of H₂O₂ and superoxide anion (O₂·^–^) Indirectly support redox homeostasis

Nrf2, nuclear factor erythroid 2-related factor 2; ROS, reactive oxygen species; RNS, reactive nitrogen species; SOD, superoxide dismutase; GPx, glutathione peroxidase; ROO·, peroxyl radical; ·OH, hydroxyl radical; Fe²⁺, ferrous ion; α-, beta; β-, beta; γ-, gamma; δ-, delta; ¹O₂, singlet oxygen; H₂O₂, hydrogen peroxide; O₂·^–^, superoxide anion.

The earliest report assessing the impact of a dietary antioxidant, 1200 IU/day of vitamin E for two weeks, on exercise performance was conducted by Dillard and colleagues [35], who found that vitamin E blunted the increase in pentane. This was thought to occur when vitamin E donated a hydrogen atom to lipid ROO·, thereby interrupting lipid peroxidation. Other antioxidants, such as vitamin C, can help “recycle” vitamin E by reducing it back to α-tocopherol, allowing it to continue serving as an antioxidant [114–116]. Vitamin C can also directly neutralize ROS, such as O₂·^–^, H₂O₂, ·OH, and ¹O₂, by donating electrons to render the ROS less reactive [117]. Other antioxidants, such as N-acetylcysteine (NAC) and astaxanthin (AST), can also directly scavenge ROS/RNS and other free radicals while also modulating the redox-sensitive NF-κB and nuclear factor erythroid 2-related factor 2 (Nrf2) pathways [118–124]. Importantly, antioxidants, such as NAC, can work as precursors to other endogenous antioxidants [125]. For example, NAC can provide cysteine, which is the rate-limiting amino acid for GSH synthesis [126]. Another key factor in how dietary antioxidants exert their free-radical neutralizing effects is their molecular structure and solubility. Lipid-soluble antioxidants, such as AST or vitamin E, are especially effective at intercepting radicals within the phospholipid bilayer [127,128]. In contrast, water-soluble antioxidants (e.g. NAC) predominantly operate in aqueous compartments (i.e. blood plasma and the cytosol) to neutralize free radicals [125]. In general, dietary antioxidants can serve as (1) direct free radical scavengers, (2) precursors to other non-enzymatic antioxidants, and (3) modulators of redox-sensitive pathways, which can reduce pro-inflammatory cytokine production and increase endogenous antioxidant enzyme expression [129].

Currently, data suggest that acute dietary antioxidant supplementation can be beneficial if the aim is to mitigate muscular fatigue and recover quickly; however, if the goal is to improve performance or training adaptations, depending on the specific nutrient in question (i.e. vitamin E or vitamin C versus AST), dietary antioxidant supplementation may not be warranted, especially given that chronic, high-dose antioxidant supplementation has been shown to impair performance and exercise adaptations [1,2,4,34,130]. Previous work has demonstrated that there are variations in individual EIOS responses to training, which may offer valuable insights into why some studies report differing results with exercise training and antioxidant treatment [10,131–134]. For instance, Margaritelis and colleagues [131] found that acute, high-intensity eccentric, muscle-damaging exercise led to minimal or no oxidative stress, or even reductive stress, for more than one out of every three individuals among 98 young males. These observations suggest that a person's redox status should be evaluated beforehand when examining the effects of antioxidant intake on EIOS. Margaritelis et al. [132] proposed that if an individual is deficient in a specific nutrient, supplementation with that specific antioxidant may be warranted and could support performance-related outcomes. However, concluding that antioxidant supplementation is necessary solely based on oxidative stress is not ideal, as interindividual differences likely influence how an individual responds to EIOS and subsequent antioxidant intake [135]. There are likely many reasons (e.g. health and/or performance) for an individual to supplement with an antioxidant. Nevertheless, individuals must consider whether they may be deficient in a dietary antioxidant and the strength of the evidence supporting its use.

## 9 Common dietary sources of antioxidants: a food-first framework

Exogenous antioxidants include vitamins, minerals, flavonoids, anthocyanins, tocopherols, polyphenols, and carotenoids, which can be obtained from whole foods and/or dietary supplements [136,137]. In general, athletes can obtain optimal amounts of exogenous antioxidants through a well-balanced diet. Nevertheless, athletes and sports performance professionals need to understand which common whole food sources are rich in dietary antioxidants (see Table 1) to (1) ensure optimal intake through diet and (2) identify potential gaps due to personal preferences, dietary restrictions, or food allergies. In such cases, targeted supplementation may be warranted to compensate for insufficient intake from a food-first approach.

The various foods containing these antioxidant compounds (i.e. flavonoids, polyphenols, tocopherols, and carotenoids) can leverage ROS/RNS scavenging abilities largely due to the molecular structure and classifications (i.e. water- or lipid-soluble) of these compounds. For instance, flavonoids – found in fruits, herbs, cereals, nuts, stems, vegetables, seeds, and flowers – are characterized by a benzopyran backbone consisting of two aromatic rings (A and B) and a heterocyclic C ring [138]. This structure confers phenolic or polyphenolic properties that contribute to their antioxidant activity. For example, the hydroxyl (-OH) groups attached to the A, B, or C rings can donate a hydrogen atom or an electron to a free radical (i.e. ROO·), or the 3',4'-dihydroxy groups on the B ring or the 3-OH and 4-keto groups on the C-ring can allow for the chelation of redox-active metals, preventing Fenton reactions to reduce the potential generation of ·OH. Anthocyanins, a subclass of flavonoids, contain a flavylium ion that gives them red, blue, or purple pigmentation (depending on pH). These compounds have the same C6-C3-C6 skeleton as do other flavonoids; therefore, anthocyanins can also donate a hydrogen atom or an electron from their -OH groups to neutralize free radicals (i.e. O₂·^–^, ·OH, ROO·, (¹O₂) or chelate transition metals to prevent the formation of ·OH. Another important function of these compounds is indirect antioxidant effects through the activation of Nrf2 signaling, demonstrating that dietary antioxidants not only directly neutralize ROS/RNS but also support endogenous antioxidant defenses. Table 1 provides a general overview of each dietary antioxidant class and its mechanism of action.

## 10 Dietary supplementation: short- and long-term effects in exercise and sport

Observational studies have reported that approximately 50%–100% of athletes supplement with vitamins, antioxidants, or other substances to enhance performance and immune function [139–141]. A wide array of dietary antioxidant supplements are marketed for their ability to promote recovery and mitigate exercise-induced oxidative damage and inflammation. Identifying clinically relevant antioxidants can be challenging because of the large number of bioactive compounds with antioxidant activity. Although many of these compounds exhibit antioxidant activity in preclinical studies, they may lack robust clinical evidence to confirm their efficacy or have not been assigned an RDI by the FDA. As such, selecting an appropriate dietary antioxidant is often more nuanced than choosing other ergogenic aids, such as beta-alanine, which has a well-defined mechanism for enhancing high-intensity exercise performance by buffering hydrogen ions.

Several factors can influence an individual’s response to dietary antioxidant supplementation, including (1) training status (e.g. sedentary, recreationally active, resistance- or endurance-trained); (2) baseline endogenous antioxidant capacity and oxidative stress levels; (3) sex and gender differences; (4) age-related changes in redox homeostasis; (5) overall nutritional status and habitual dietary antioxidant intake; (6) the type, dosage, and timing of antioxidant administration; (7) the chemical form and bioavailability of the antioxidant compound; (8) the type, intensity, and duration of the exercise stimulus; and (9) environmental factors or air quality. Therefore, it is crucial for the athlete or the professional working with the athlete or individual to consider these factors in addition to the body of evidence supporting claims made about an antioxidant.

There are arguments for and against the use of dietary antioxidants among athletic and exercise-training populations. Further research warranted to establish whether the dietary antioxidant in question is beneficial or detrimental to training adaptation or performance, given the respective training or sport-specific setting [9]. Current evidence suggests that during periods of intensified training, such as planned overreaching phases or consecutive days of competition, antioxidant supplementation may offer benefits, particularly when the primary goal is to enhance short-term recovery [4]. For athletes undergoing such phases, reductions in subjective or objective markers of muscle soreness or improvements in recovery kinetics may lead to better preparedness for subsequent performance. However, for athletes outside these demanding conditions, it remains unclear whether acute improvements in recovery, attributed to reduced muscle damage, ultimately translate into meaningful performance gains. Table 2 summarizes all the dietary antioxidants discussed subsequently, along with the level of evidence supporting their inclusion in exercise and sport.

**Table 2.** Level of efficacy of common dietary antioxidant.

Antioxidant	Mechanism of action	Potential benefits	Dosage & duration	Level of evidence to support efficacy	
				Performance	Antioxidant
Alpha-lipoic acid	Scavenges ROS, recycles glutathione, metal chelation	May aid recovery, reduce muscle damage and oxidative stress	100–1800 mg/day for up to 6 months	Weak/Low	Weak/Low
Ashwagandha	Scavenges free radicals, modulates Nrf2 and NF-κB pathways	Improves strength, power, recovery; modulates cortisol/testosterone	240–1250 mg/day for ≥4–12 weeks	Weak-to-moderate	Weak/Low
Astaxanthin	Lipid membrane protection, scavenges ROS/RNS	Enhances fat oxidation, reduces muscle damage, improves recovery	4–12 mg/day for 4–12 weeks	Moderate	Weak-to-moderate
Beetroot	Nitrate donor, improves oxygen efficiency (details pending)	Improves oxygen efficiency, may enhance endurance	250–500 mL/day of beetroot juice (containing 300–600 mg of nitrates) for single, 3–15 days, or up to 4–6 weeks	Moderate-to-high	Weak/Low
Blackcurrant	High anthocyanins; antioxidant; NO signaling; mitochondrial protection	Improves endurance and increases fat oxidation	300 mg/day extract providing 105–210 mg anthocyanins for 7 days; 1–2 h pre-exercise	Moderate	Weak/Low
Cocoa flavanols	Flavanol-rich extract, supports NO production, improves endothelial function	May improve vascular function and reduce exercise-induced oxidative stress	430–700+ mg CF ~2 h pre-exercise or 200–1000 mg/day for 2 weeks–3 months	Weak/Low	Weak/Low
Coenzyme Q10	Supports ETC, scavenges ROS, regenerates vitamins E & C	Reduces oxidative stress, limited performance impact	30–600 mg/day for 2–12 weeks	Weak/Low	Moderate
Curcumin	Modulates NF-κB, direct antioxidant, anti-inflammatory	Reduces pain, inflammation, may improve performance short-term	180–2000 mg/day for 3 days–3 months	Weak/Low	Moderate
Creatine monohydrate	Possible ROS scavenging, anti-inflammatory cytokine effects	Performance enhancement with potential antioxidant/anti-inflammatory support	20 /day loading or 0.1 g/kg/day for 5–10 weeks	High	Weak–moderate
Fucoxanthin	Carotenoid-based ROS scavenger, anti-inflammatory	Improves aerobic capacity, reduces inflammation; cognitive support	2–21 mg/day for 2–16 weeks	Weak/Low	Weak/Low
Glutathione/NAC	Boosts glutathione synthesis; NAC supports redox balance	Increases glutathione, supports aerobic performance in deficient individuals	Glutathione: 200–1000 mg/day, NAC: 1.2–2.0 g/day	Weak-to-moderate	Weak-to-moderate
Green Tea Catechins (EGCG)	Potent flavonols; antioxidant; Nrf2 activation; supports redox enzymes	Improves antioxidant capacity, modest fat oxidation, small soreness reductions	400–800 mg/day catechins or 2–3 cups tea/day for 7–14 days; avoid acute high-dose pre-exercise	Weak-to-moderate	Weak-to-moderate
Lutein & Zeaxanthin	Scavenge ROS, support vision/cognition, concentrated in retina	Improves visual acuity, reaction time, possible muscular benefits	10–34 mg/day for 8–12 weeks	Weak/Low	Weak/Low
Omega-3 fatty acids	Reduces ROS via mitochondrial & Nrf2 mechanisms	Reduces inflammation and oxidative stress, supports recovery	1000–6000 mg/day EPA + DHA for 6–12 weeks	Moderate	Moderate–High
Pomegranate polyphenols	Ellagitannins/polyphenols; antioxidant and anti-inflammatory	Improves recovery from eccentric exercise; reduces soreness; beneficial hemodynamics	500–1000 mL/day juice or extracts providing 500–1000 mg polyphenols for 3–7 days pre-/post-exercise	Weak-to-moderate	Moderate
Pycnogenol	Neutralizes ROS, enhances endogenous antioxidants, NOS modulation	Improves endurance, cognitive function, recovery	60–200 mg/day for 4–12 weeks	Weak/Low	Weak/Low
Quercetin	antioxidant/free-radical scavenging; activation/upregulation of Nrf2 antioxidant pathway; modulation of AMPK–SIRT1–PGC-1α signaling promoting mitochondrial biogenesis; anti-inflammatory effects	Reduces soreness, supports mitochondrial health, performance potential	~500–1000 mg/day (commonly 500 mg twice daily or 1000 mg/day); acute (single-dose or 7 days) to subacute (1–4 weeks) and some chronic trials up to 8 weeks	Weak-to-moderate	Weak-to-moderate
Resveratrol	Activates SIRT1, AMPK, Nrf2, affects mitochondrial function	May improve endurance and mitochondrial function, but could blunt training	250–480 mg/day for 4–8 weeks	Weak/Low	Weak/Low
Selenium	Cofactor for GPx, thioredoxin reductase, reduces IL-6	Supports antioxidant enzyme function, reduces inflammation	50–250 μg/day for 3–42 weeks	Weak/Low	Weak/Low
Spirulina	Increases GSH, SOD, vitamin C, anti-inflammatory	Improves antioxidant status, may reduce oxidative stress	500–10,000 mg/day for 15–60 days	Weak/Low	Weak/Low
Sulforaphane	Activates Nrf2, indirect antioxidant, anti-inflammatory	Enhances recovery, reduces soreness, does not blunt adaptation	20–40 mg/day for 2–4 weeks	Weak/Low	Weak/Low
Tart Cherry	Anthocyanins/polyphenols reduce DOMS, inflammation	Reduces DOMS, supports sleep and inflammation control	480 mg powder or 60–90 mL juice for 7–14 days	Moderate-to-high	Moderate
Urolithin A	Enhances mitophagy and mitochondrial efficiency; reduces inflammation	Improves muscular endurance, fat oxidation, VO2max response, reduces muscle damage	500–1000 mg/day for 4–8 weeks	Weak/Low	Weak-to-moderate
Vitamins E & C	Direct scavengers, regenerate each other, mixed effects	Antioxidant defense, may reduce fatigue; may blunt training at high doses	Vit E: 400–800 IU/day, Vit C: 1000 mg/day	Weak/Low	Moderate–High
Zinc	Cofactor for Cu/Zn-SOD, Nrf2 activation, membrane stabilizer	Enhances SOD activity, reduces oxidative/inflammatory biomarkers	50–100 mg/day for 2–16 weeks	Weak/Low	Weak/Low

The dosing and duration ranges presented for several supplements are extremely broad, which may reduce the table’s usefulness for synthesis. Many of these intervals (e.g. ALA 100–1800 mg; ashwagandha 240–1250 mg; and curcumin 180–2000 mg) reflect the full scope of published studies rather than the most evidence-supported dosing. To improve clarity and consistency, I recommend revisiting these values and potentially narrowing them to typical or commonly used ranges in RCTs; ROS, reactive oxygen species; RNS, reactive nitrogen species; ETC, electron transport chain; GPx, glutathione peroxidase; IL-6, interleukin 6; GSH, glutathione; SOD, superoxide dismutase; Cu/Zn-SOD, copper/zinc superoxide dismutase; NF-κB, nuclear factor kappa-light-chain-enhancer of activated B cells; Nrf2, nuclear factor erythroid 2-related factor 2; SIRT1, sirtuin 1; AMPK, AMP-activated protein kinase; NOS, nitric oxide synthase; DOMS, delayed onset muscle soreness; Vit E, vitamin E; Vit C, vitamin C; EPA, eicosapentaenoic acid; DHA, docosahexaenoic acid; mg, milligrams; mL, milliliters; g, grams; μg, micrograms; IU, international units; kg, kilograms.

## 11 Key findings and strategies for dietary antioxidants in exercise and sports

### 11.1 Alpha-lipoic acid

Alpha-lipoic acid (ALA), also known as thioctic acid, serves as a cofactor for various multi-enzyme complexes (e.g. pyruvate dehydrogenase, α-ketoglutarate dehydrogenase, and branched-chain ketoacid dehydrogenase), which play a role in the oxidation of keto acids, energy production, and amino acid metabolism [142]. In terms of its antioxidant capabilities, ALA can recycle endogenous glutathione; scavenge hydroxyl and peroxide radicals, singlet oxygen, and hypochlorous acid; and form chelate complexes with metal ions [143,144]. To date, these antioxidant effects of ALA have primarily been shown in clinical human trials with Alzheimer’s disease [145] and diabetic patients [146–148], as well as overweight/obese adults [149]. Doses ranging from 100 to 1200 mg/day of ALA have been evaluated, with higher doses up to 1800 mg/day for up to 6 months. Nevertheless, only a handful of studies have examined the application of ALA for exercise and sports performance [150–152]. First, Fogarty et al. [152] reported that daily supplementation with 1000 mg/day of ALA for 14 days among 12 healthy males could selectively protect against DNA oxidation, as demonstrated by participants expressing (1) 5.4% shorter DNA tail length (indicating DNA fragmentation and damage within cells) and (2) lower (*p* < 0.05) plasma 8-OHdG (2.1%), lipid hydroperoxides (7.75%), protein carbonyls (70%), and hydrogen peroxide (0.4) levels following a muscle-damaging exercise protocol. Then, Morawin et al. [151] assessed the effects of ALA (i.e. 1200 mg/day of thiogamma for 10 days) on erythropoietin release following an exercise bout consisting of 90 min of running followed by 15 min of eccentric running at 65% V̇O_2_max at a −1% gradient among 16 healthy young adults. They found that ALA increased erythropoietin release following eccentric running exercise and decreased H_2_O_2_ and other markers of oxidation, but did not affect creatine kinase (CK). Finally, Isenmann and colleagues [150] assessed the effects of both single-dose acute (150 mg dose) and short-term (i.e. 2 × 150 mg/day for 6 days) ALA supplementation on muscle strength recovery and performance among 17 male resistance- and endurance-trained athletes in a randomized, placebo-controlled, crossover manner. A single dose of ALA did not affect 1-RM back squat strength or biomarkers of skeletal muscle damage, oxidative stress, or inflammation. However, the short-term ALA supplementation protocol resulted in participants maintaining their 1-RM back squat strength compared with placebo, which declined and was lower at the final time point. Collectively, reports on ALA suggest that one week of supplementation may reduce biomarkers of muscle damage and oxidation; however, more research and longer studies are needed before definitive recommendations can be made regarding supplementation strategies.

### 11.2 Ashwagandha

Ashwagandha (ASH; *Withania somnifera*) is an emerging adaptogenic compound that is considered a fundamental Ayurvedic remedy. ASH has garnered significant attention over the last decade for its potential anti-stress, antioxidant, anti-inflammatory, and immunomodulating properties [153]. ASH is supplemented primarily as a root extract and is known for mediating the neuroendocrine response (i.e. cortisol/testosterone) and various oxidative stress and inflammatory biomarkers, with the potential to impact muscle strength, power, cardiorespiratory fitness, and recovery [153,154]. Recently, ASH has gained increased attention for its antioxidant and anti-inflammatory properties, which may enhance physical performance and recovery [155,156]. ASH contains several bioactive compounds, including withanolides (e.g. withaferin A, withanolide D), alkaloids, sitoindosides, flavonoids, tannins, and polyphenols. It is thought to modulate cellular pathways, such as Nrf2 (promoting endogenous antioxidants such as SOD, CAT, and GPx) and NF-κB (reducing pro-inflammatory cytokines) [155–157]. These mechanisms may aid post-exercise recovery, and some studies have reported positive effects on strength and body composition [158,159].

Few studies have examined the effects of ASH on exercise and sports performance. Bonilla et al. [155] conducted a meta-analysis demonstrating ASH’s effectiveness in improving strength and power outcomes at doses of 240–600 mg/day for ≥6 weeks, though most data were obtained from untrained populations. Furthermore, Wankhede et al. [158] assessed the effect of 300 mg/day of ASH and found improvements in bench press and leg extension strength compared to the placebo group. In addition, the ASH group also experienced greater increases in arm muscle cross-sectional area and chest girth, as well as a greater reduction in body fat percentage [158]. Ziegenfuss et al. [160] found that 12 weeks of ASH supplementation at 500 mg/day, coupled with a resistance training program, resulted in greater squat and bench press strength than the placebo. Additionally, only the ASH group showed improvements in upper- and lower-body power and perceived recovery scores [160]. Finally, Raut et al. [161] reported increased muscular force production during handgrip, quadriceps, and back extensor exercises after 30 days of supplementation with increasing doses of 750–1250 mg/day of ASH in physically active, healthy individuals. Collectively, these findings demonstrate that ASH supplementation can improve performance and recovery; however, more research is warranted.

### 11.3 Astaxanthin

Astaxanthin, a lipid-soluble, red-orange-colored ketocarotenoid, has attracted attention for its potent antioxidant and anti-inflammatory properties [118–122,162–164]. The effective dosage and duration of AST supplementation (*Haematococcus pluvialis* microalgae) range from 4 to 12 mg/day for 4–12 weeks to favorably impact cardiometabolic health outcomes [119,165] and reduce inflammatory and oxidative stress biomarkers [166–182]. The unique structure of AST, which has two β-ionone rings connected by a polyene chain, allows it to cross the phospholipid membrane thickness and neutralize ROS/RNS both at the membrane periphery and inside the lipid bilayer [183,184], is naturally found in salmon, lobster, shrimp, and crab. This mechanism may increase fatty acid oxidation during exercise [185] and attenuate or limit EIOS, inflammation, and muscle damage, ultimately promoting quicker recovery [172,186–189]. Owing to its limited dietary availability in whole foods, supplementation with AST is needed for ergogenic benefits. AST is generally recognized as safe (GRAS), and the only notable side effect occurs when it is incompletely absorbed in the gut and is excreted in the stool, imparting a red color [128].

Several studies support the use of AST as potentially beneficial for athletes. For instance, Fleischmann et al. [188] and Sawaki and colleagues [190] found reduced postexercise blood lactate concentrations following supplementation protocols of 12 mg/day AST for 30 days and 6 mg/day AST for 4 weeks, suggesting improved metabolic recovery. Malmsten et al. [191] reported that the use of 4 mg/day AST over six months, when combined with exercise, increased the number of barbell squat repetitions by 55% in paramedic students. With respect to substrate utilization, Brown and colleagues [192] reported that 12 mg/day AST for 7 days increased fat oxidation (≈69%) and reduced respiratory exchange ratios (≈3%) in trained male cyclists. However, most studies have not demonstrated an effect of AST on substrate utilization [189,193,194]. AST has also shown promise in attenuating biomarkers of EIOS, muscle damage, and inflammation following intense exercise [168,172]. For example, Baralic et al. [168] reported increased antioxidant enzyme activity (≈17% –≈42%) following 90 days of 4 mg/day AST supplementation in soccer players, with a follow-up study finding that the increase in high-sensitivity C-reactive protein (CRP) levels with training was attenuated in the AST group [167]. Finally, Djordjevic et al. [172] demonstrated that elite soccer players who ingested 4 mg/day AST for 90 days displayed a blunted increase in postexercise blood creatine kinase levels compared to those receiving placebo (≈21% and ≈29%, respectively). Taken together, these findings demonstrate the ergogenic value and antioxidant and anti-inflammatory benefits of AST in the context of sport and exercise.

Studies using AST have shown either no adverse effects [187] or even a positive effect on performance and training adaptation [191,195]. For example, Barker and colleagues [187] reported that 12 mg/day AST for 4 weeks did not affect resistance training outcomes but reduced muscle soreness (≈57%; *p* = 0.01) and perceived muscle damage (≈60%; *p* = 0.02) 24 hours postexercise. This finding demonstrates the ability of ASTs to enhance recovery without impairing training adaptations. While this may seem minimal, one cannot overlook the importance of improving an individual’s perception of soreness or recovery, as this may lead to improved work output in training and competition [196].

### 11.4 Beetroot

Beetroot (*Beta vulgaris*) is a nitrate-dense root vegetable that also contains a range of bioactive compounds, including betalains, phenolic acids, flavonoids, ascorbic acid, and carotenoids. These constituents possess antioxidant and anti-inflammatory properties that may offer benefits in both clinical and athletic contexts [197–199]. However, the focus of beetroot supplementation has focused primarily on its nitrate content and NO-mediated effects; emerging evidence suggests that other phytochemicals, particularly betalains, may also contribute to performance and recovery. Betalains have demonstrated the ability to reduce reactive oxygen and nitrogen species (ROS/RNS), modulate inflammatory gene expression, and aid recovery from exercise-induced muscle damage [200]. A recent review even suggested that beetroot juice may outperform nitrate salts alone because of these additional compounds and their combined effects on NO production, mitochondrial function, and vascular health [201]. As such, beetroot supplementation has been explored as a strategy to mitigate oxidative stress, reduce inflammation, and enhance recovery [197,201].

Despite these promising mechanisms, evidence regarding the effects of beetroot on inflammation and oxidative stress is inconsistent, with a series of studies by Clifford et al. [202–205]. demonstrated no impact on markers of EIOS or inflammation following strenuous exercise protocols, including drop jumps, sprint tests, and marathon running; however, some noteworthy ergogenic and recovery effects were observed. First, Clifford et al. [203] examined the effects of single doses of beetroot juice (250 mL or 125 mL) versus placebo on recovery and inflammation after eccentric exercise in 30 recreationally active males. The 250 mL group showed faster recovery in countermovement jump performance at 48 and 72 hours postexercise and reported higher pressure pain thresholds. However, beetroot juice had no effect on markers of exercise-induced oxidative stress, inflammation, or muscle damage. Clifford et al. [204] found that 2 × 250 mL/day for 3 days of beetroot juice supplementation led to quicker recovery for the countermovement jump (7.6%) and relative strength index (13.8%) than did a placebo, with no effect on the biochemical markers among 20 male, team-sports players. Their third study [205] revealed that 3 days of supplementation with beetroot juice (containing ≈210 mg nitrate and ≈405 mg/GAE/L polyphenol content), sodium nitrate (containing ≈210 mg nitrate but no polyphenols), or placebo did not impact any biomarkers following eccentric drop jumps, but the beetroot juice group experienced an attenuation of their pressure pain threshold among 30 recreationally active males. Finally, Clifford and colleagues [202] conducted a marathon field study among 34 experienced runners who consumed either beetroot juice or a placebo during the 3 days post-marathon recovery phase and found no effect on any biomarkers of EIOS or inflammation. Interestingly, Kozłowska et al. [206] demonstrated that 4 weeks of 26 g/day beetroot juice supplementation increased GPx-1 and GPx-3 activity, elevated β-carotene, reduced malondialdehyde (MDA) and advanced oxidation protein products (AOPP), and improved V̇O_2_max. In addition, Daab et al. [207] demonstrated that 2 × 150 mL/day of beetroot juice supplementation for 7 days (3 days pre- and post-exercise) led to an attenuated decrease in countermovement jump performance and maximal voluntary contraction output during the recovery days post-exercise, while no effect was noted for CK, lactate dehydrogenase (LDH), and CRP. Finally, findings from Vilar et al. [208] reported among 32 ultra-endurance runners participating in a 107-km mountain race demonstrated no effect on EIOS or inflammatory biomarkers. Taken together, there appears to be a benefit in terms of recovery, as shown by the attenuated decrease in countermovement jump performance; however, there is little to no effect on EIOS or inflammation.

Similarly, recent systematic reviews have highlighted the inconsistency in the effects of beetroot supplementation on markers of inflammation and oxidation [209–211]. For example, Jones et al. [209] concluded that beetroot consistently improved functional recovery and soreness but did not influence systemic CRP, IL-6, IL-8, tumor necrosis factor-α (TNF-α), or lipid peroxidation products. Based on six studies that measured markers of inflammation, Rojano-Ortega et al. reported no significant differences between beetroot and placebo [210]. Additionally, beetroot supplementation did not attenuate oxidative stress compared with placebo in three of the four included studies [210]. Only one study reported significant changes in GPx-1 and MDA; however, the authors postulated that these changes were most likely reflective of training-related adaptations rather than direct antioxidant effects [206]. Beetroot (as nitrate-rich juice) appears to aid in functional recovery and some performance outcomes; however, its effects on systemic inflammatory and oxidative stress biomarkers are inconsistent. It is important to note that, based on the ISSN 2018 position stand, nitrate supplementation is recommended with either beetroot or sodium nitrate, at amounts ranging from 300 to 600 mg, 2–3 h before exercise (acute/single dose), as well as for daily use for up to a duration of approximately 6 weeks [12].

### 11.5 Blackcurrant anthocyanins

Blackcurrant is a type of berry, similar to blackberries and blueberries, that has been studied for their health-promoting benefits, primarily because of their high polyphenolic and anthocyanin contents [212]. In general, berries contain high concentrations of flavonoids, particularly anthocyanins, which are natural pigment responsible for the blue, purple, red, and orange colors of many fruits and vegetables, which are suggested to confer significant health benefits [213]. To this end, blackcurrant anthocyanins (delphinidin-3-rutinoside) contain between 130 and 460 mg/100 g of fruit weight of total anthocyanins, while the total daily estimated consumption amounts range from 3 to 215 mg/d [213]. Furthermore, blueberries contain approximately 62–300 mg/100 g of fruit weight of total anthocyanins, suggesting that blackcurrants may be more ideal sources of anthocyanins [213]. Tang et al. [214] demonstrated that blackcurrant anthocyanins have the ability to attenuate inflammation and oxidative stress while preventing the depletion and damage to the mitochondrial content within an animal model, further supporting the potential for blackcurrant as an effective dietary antioxidant, which may be a better source to leverage in the diet or via supplementation in comparison to other sources such as blueberry anthocyanins.

To date, New Zealand (NZ) blackcurrants appear to be the most highly concentrated in terms of anthocyanins and other phytochemicals compared to those from other countries. Schrage and colleagues found that the NZ blackcurrants contain between 336 and 850 mg/100 mL in juice versus the 170 and 310 mg/100 mL found in non-NZ blackcurrants. Considering this, several research groups have investigated the benefits of blackcurrant supplementation for exercise recovery and sports performance [215–227]. For instance, Willems et al. [227] assessed the impact of 1 week of 300 mg/d of blackcurrant extract supplementation in nine male endurance athletes (i.e. trained cyclists or triathletes with more than 3 years of experience). They found that there was an improvement in the 16.1 km (10-mile) time-trial cycling performance (3.6%) [227]. Another research group [225] also demonstrated that supplementation with blackcurrant juice (containing 300 mg of anthocyanins and 15 mg of vitamin C) resulted in a 1.9% improvement in peak running speed among 23 trained female runners. Furthermore, Cook et al. [228] found that NZ blackcurrant extract supplementation (containing 300 mg/d of CurraNZ*™* and 105 mg of anthocyanins) led to a 27% increase in fat oxidation at 65% of participants’ VO_2_max, alongside a 2.4% improvement in their 16.1 km time-trial performance. Recently, Braakhuis et al. [215] conducted a systematic review and meta-analysis on the effects of NZ blackcurrant within the context of sports performance and found that across nine total studies, there was a 0.45 (95% confidence interval [CI] = 0.09–0.81, *p* = 0.01) mean percent effect on performance. The authors noted that this improvement in sport-specific performance-related outcomes is not only relevant to athletes but also within the magnitude of effect for medal-winning at the Olympic level of competition [215]. Furthermore, the magnitude of the effect is similar to that observed with caffeine (i.e. 0.41, 95% CI = 0.15–0.68, *p* = 0.002) [229]. Interestingly, this meta-analysis by Braakhuis et al. [215] did not affect the reduction in oxidative stress or inflammatory biomarkers. The authors attributed this negative result to the low number of studies included; therefore, they suggested that meaningful conclusions could not be made with respect to these biomarkers. Nevertheless, the present data suggest that NZ blackcurrants can confer a small but meaningful improvement in athletic performance following 7 days of treatment with 105–210 mg blackcurrant anthocyanins when taken between 1 and 2 h before exercise [215].

### 11.6 Cocoa flavanols

Flavonoids are polyphenols composed of two phenyl rings and a heterocyclic ring, and include flavanols, flavonols, isoflavones, flavones, and anthocyanidins. Recent interest has been shown in the application of flavonols from various food sources, including cocoa, wine, fruits, vegetables, and teas. Notably, cocoa, which comes from the seeds of the Theobroma cacao tree’s fruit, is a rich source of flavonols [230]. Cocoa flavanols (CF) are of interest to athletic populations because of their ability to stimulate nitric oxide production, thereby improving vasodilation and endothelial function [231]. Endothelial function, measured by flow-mediated dilation, improves, a benefit acknowledged by the European Food Safety Authority (EFSA), which recommends an intake of approximately 200 mg/day of CF to help sustain normal endothelium-dependent vasodilation. This effect peaks approximately 2 h after ingestion [232,233]. A 2018 systematic review by Decroix et al. [231] noted that CF can improve vascular function, potentially alter substrate oxidation rates, and lessen exercise-induced oxidative stress; however, the authors concluded that more research is needed to assess the chronic effects of CF with and without exercise training. Furthermore, much of the research to date has concluded that the results are equivocal regarding the effects of CF on exercise-induced oxidative stress and exercise performance [234–241]. Nevertheless, Decroix et al. [231] concluded that acute CF supplementation of approximately 400–500 mg or higher 2 h before exercise, or even 2–3 months of CF supplementation, may aid in reducing exercise-induced oxidative stress and improving vascular function during exercise bouts. Furthermore, while the ideal dosage for CF consumption remains a topic of debate, higher total flavanol intake (>700 mg, particularly higher epicatechin intake >80 mg) has been shown to confer beneficial effects.

### 11.7 Coenzyme Q10

Coenzyme Q10 (CoQ10), also known as ubiquinone or ubiquinol, is a naturally occurring, fat-soluble biochemical cofactor that plays a role in energy production and acts as an endogenous antioxidant. Notably, CoQ10 can increase the production of endogenous antioxidant enzymes, such as SOD, or, when reduced to ubiquinol (Q10 H_2_), it can exert a direct antioxidant effect through free radical scavenging and electron donation to neutralize ROS/RNS [242]. CoQ10 has also been shown to regenerate vitamins E and C and reduce the expression of inflammatory biomarkers [243,244]. Given this, researchers have assessed the impact of CoQ10 supplementation on ROS/RNS in disease conditions, such as cardiovascular disease, diabetes, and hypertension [245–247]. Numerous studies have assessed the impact of CoQ10 supplementation on exercise performance [248–267], with doses ranging from 30–300 mg/day in healthy athletes to >600 mg/day in disease populations, for 2–12 weeks. Among these studies, several failed to demonstrate any impact of CoQ10 supplementation on performance and sport-related outcomes [248,249,251,252,255–257,260,262,264,265,267,268]. However, reports have shown favorable outcomes in reducing biomarkers of oxidative stress [253,258–260,264,269] and muscle damage [253,254,259,260,264]. For example, Emami and colleagues [260] found that taking 300 mg daily for 14 days prevents negative effects on myocardial damage and oxidative stress during the swimming competition phase. While CoQ10 has beneficial effects on attenuating biomarkers of inflammation, EIOS, and muscle damage, most studies demonstrate minimal to mixed effects of short- and long-term supplementation on performance-related outcomes [270–272].

### 11.8 Curcumin

Curcumin (1,7-bis(4-hydroxy-3-methoxyphenyl)-1.6 heptadiene-3,5-diona) is the main phenolic compound found in turmeric (*Curcuma longa L.*), which is the oriental yellowish-colored spice of the ginger (*Zingiberaceae*) family. Curcumin has been investigated as an adjunctive treatment for its pain-reducing [273,274] and anti-inflammatory properties [275] and has emerged as a medicinal treatment for chronic/cardiometabolic disease states [276–278]. In the context of exercise and sports performance, several studies have demonstrated a potential beneficial effect of curcumin supplementation, with few to no adverse side effects [279–289]. Suhett and colleagues [290] conducted a systematic review of the literature on the application of curcumin supplementation in sport and physical exercise and found that several studies demonstrated (1) antioxidant and anti-inflammatory effects [279,281–286,288], (2) ergogenic properties following various supplementation protocols (i.e. both short- and long-term) [280,284,287,288], and (3) reductions in pain perception and muscle damage [284,288,289,291]. For example, Delecroix et al. [280] assessed the impact of a single 2 g dose of curcumin co-ingested with 20 mg of piperine among 10 elite rugby players following an exercise-inducing muscle damage protocol and found a lower loss of mean power during sprinting with the curcumin treatment (1.77% loss) compared to the placebo (13.6% loss). Furthermore, 6–10 mg/day of curcumin appears to be associated with reduced pain and muscle damage [280,284,288,289]. In terms of longer-term benefits, data suggest an antioxidant effect (i.e. lower advanced glycation end products and MDA) following 3 months of curcumin supplementation (10 mg/day) [291]. Suhett and colleagues [290] suggested that doses ranging from 180 to 500 mg/day for 3–7 days are efficacious for improving performance and recovery-related parameters following exercise. However, long-term data are limited, and most of the current literature surrounding curcumin suggests that performance and recovery benefits are observed only with short-term supplementation [290]. In summary, despite its poor bioavailability [292], short-term curcumin supplementation (i.e. up to 1 week) at 180–500 mg/day has been shown to improve performance and recovery-related outcomes. Limited data suggest that longer supplementation periods may be beneficial; however, more research is warranted to demonstrate repeatability.

### 11.9 Creatine monohydrate

Although not considered a traditional antioxidant, creatine monohydrate (CrM), arguably the most researched ergogenic aid [293,294], has been purported to possess indirect antioxidative properties [295]. Studies have demonstrated that CrM can reduce free-radical-induced damage and inflammation following exercise in animal and human models [296–304]. Nevertheless, the mechanism by which CrM acts as an antioxidant is not fully understood, with some reports suggesting that CrM can increase endogenous antioxidant enzyme activity and the capacity to neutralize ROS/RNS [305–307]. Relatively few human studies have investigated the antioxidant impact of CrM, with mixed results reported to date. Kingsley et al. [308] demonstrated that 7 days of CrM loading dose (20 g/day) did not favorably impact the levels of markers of lipid peroxidation or plasma concentrations of non-enzymatic antioxidants. However, Rahimi [304] found that 20 g/day of CrM supplementation for 7 days reduced urinary 8-OHdG and plasma MDA levels in response to resistance training. Then, Amiri and Sheikholeslami-Vatani [297] found that 10 weeks of resistance training and CrM supplementation (0.1 g/kg/day) increased GPx enzyme levels. Despite these favorable findings, one report suggested that CrM could act as a pro-oxidant [309]. In terms of anti-inflammatory effects, reports have shown that CrM supplementation can act on specific immune cells and reduce the concentrations of the pro-inflammatory cytokines TNF-α and prostaglandin E_2_ (PGE_2_), eliciting decreased inflammation post-exercise [310–313]. For instance, Santos et al. [310] reported that CrM supplementation (20 g/day for 5 days) attenuated changes in PGE_2_ (60.9%) and TNF-α (33.7%) among runners, while those receiving the placebo exhibited increases in PGE_2_ (6.6-fold) and TNF-α (2.34-fold). Furthermore, Bassit et al. [299] demonstrated that 20 g/day for 5 days of CrM supplementation before completing an Ironman reduced the increase in IL-1, IL-6, INF-α, and TNF-α. These findings have been similarly supported by another report from Rawson et al. [314]. However, there are still mixed results regarding CrM’s anti-inflammatory activity, warranting further research to better understand its antioxidant benefits as a supplement. Notably, CrM is one of the rare dietary supplement with a substantial body of evidence supporting its efficacy as an ergogenic aid [293,294]. Therefore, while CrM may be considered to have a low level of support for its antioxidant ability, it is one nutrient that should be considered for its primary benefit (i.e. exercise performance enhancement), and the potential added benefits (i.e. antioxidant/anti-inflammatory) likely serve as an “add-on.”

### 11.10 Fucoxanthin

Fucoxanthin, a naturally occurring carotenoid found in brown seaweeds, macroalgae, and diatoms [315,316], has a unique structure featuring an allenic bond and nine conjugated double bonds that enhance its stability and antioxidant activity [317,318]. Fucoxanthin is metabolized into Fucoxanthinol and Amarouciaxanthin A, which are considered bioactive compounds. Furthermore, although fucoxanthin is sensitive to heat and air, its bioactivity also stems from its conjugated bonds and high molecular polarity [315,319]. Emerging research suggests that microalgae-based supplements, such as *Phaeodactylum tricornutum (*PT), which contain EPA, DHA, and fucoxanthin, may offer anti-inflammatory and performance-supportive benefits for athletes [315,320–323]. For instance, Stievatter and colleagues [324] demonstrated that PT supplementation (i.e. whole PT containing 294 mg of EPA+DHA and 21 mg of fucoxanthin) for 2 weeks reduced IL-6 levels (46%) and improved functional performance (sit-to-stand time) compared to placebo. In addition, Dickerson et al. [325] demonstrated that PT (4.4 mg of fucoxanthin) combined with supervised exercise training over 12 weeks led to improvements in maximal aerobic capacity, resting heart rate, subjective measures of adherence to a training program, bone mineral indices, HDL-c, and LDL-c/HDL-c ratios in women without affecting weight loss. In addition to potential muscle effects, Abidov et al. [320] conducted a study in postmenopausal women. They reported improvements in total energy expenditure after 16 weeks of high-dose (8 mg/day) fucoxanthin supplementation. There have also been reports of fucoxanthin’s ability to improve aspects of cognitive function among E-gamers [322] and elderly populations [323], attributed mainly to its antioxidant and anti-inflammatory properties, which are important outcomes related to sports performance. Taken together, fucoxanthin supplementation, particularly from the carotenoid-rich microalga PT, shows promise for impacting cardiometabolic and exercise-related outcomes. Importantly, fucoidan, a fucose-rich sulfated polysaccharides, derived from either brown seaweed or echinoderms, can improve the inflammatory and immune response after high-intensity exercise [326]. However, further work is needed in sport-specific populations to understand its impact on exercise and sports performance.

### 11.11 Glutathione and N-acetylcysteine

Glutathione is a predominant endogenous antioxidant consisting of the amino acids glutamate, cysteine, and glycine, while N-acetylcysteine (NAC) is a cysteine precursor that can be used to recycle glutathione [327]. To date, the methods of glutathione administration (i.e. oral versus intravenous) may impact the effectiveness of the dose and duration [328,329]. For instance, Schmitt et al. [329] demonstrated that an oral dose of 450 mg/day for three weeks of glutathione improved antioxidant status, while a single dose of 3000 mg delivered intravenously improved TAC [328]. It has been previously reported that exogenous glutathione is poorly stable and may degrade in the extracellular compartment, rendering it less bioavailable [82]. This may explain why most trials involving healthy adults have generally not demonstrated increases in glutathione concentrations from supplementation [330]; however, a 6-month randomized controlled trial involving apparently healthy male and female subjects reported increases in whole blood, plasma, and erythrocyte concentrations of glutathione following supplementation with low-to-high doses of glutathione (250–1000 mg daily) [331]. In the context of exercise performance, Hwang et al. [332] demonstrated that a combination of glutathione (200 mg/day) and L-citrulline (2 g/day) supplementation, coupled with resistance training for 8 weeks, increased lean mass without affecting any blood clinical chemistry parameters. Another study by Aoi and colleagues [333] found that a 1 g/day dose of glutathione for 2 weeks resulted in suppressed blood lactate levels following 60 minutes of cycling exercise. In terms of NAC, which is chemically similar to glutathione and favors glutathione synthesis, especially among individuals who are deficient in glutathione [132,334,335], the evidence is limited but promising. NAC has been shown to improve aspects of aerobic and anaerobic performance (i.e. V̇O_2_max, time trial, and anaerobic power) by 11.4%–15.4% among individuals who demonstrated baseline glutathione deficiencies. In contrast, performance was unchanged in individuals with high baseline glutathione concentrations [132,334,335]. Kerksick and colleagues [84] supplemented healthy, active males with either 1800 mg of NAC or a placebo for 14 days before a damaging bout of eccentric muscle contractions and three days after the exercise bout. Plasma markers of muscle damage and intramuscular markers of oxidative stress (8-isoprostane and SOD), inflammation (TNF-α, cortisol, neutrophils, neutrophils: lymphocytes), and apoptosis (bax, bcl-2, bax:bcl-2 ratio, and caspase-3) were assessed. NAC supplementation did not affect TNF-α, cortisol, 8-isoprostane, or SOD levels. Currently, data on NAC supplementation suggest that doses of 1.2–2.0 g/day, taken acutely or over several weeks, may affect performance outcomes, with minimal benefits [4]. Ultimately, the true ergogenic and antioxidant effects of NAC in the context of exercise and sports performance remain unclear and require further research [336].

### 11.12 Green tea catechins

Green tea catechins, specifically epigallocatechin gallate (EGCG), are part of the flavonol family, known for their potent antioxidant, anti-inflammatory, and vascular-regulating effects, which are important for exercise and recovery [337–343]. Their structure includes multiple hydroxyl groups that facilitate the neutralization of free radicals, decrease oxidative stress, and modulate nitric oxide bioavailability, potentially enhancing endothelial function and metabolic health [340]. In addition to their direct antioxidant effects, catechins enhance the body's antioxidant defenses by increasing the activity of enzymes, thereby helping the body withstand oxidative stress from exercise [338]. Human studies indicate that consistent pre-exercise catechin supplementation for at least 7–14 days can enhance overall antioxidant capacity and reduce oxidative stress markers following endurance or high-intensity exercise [340]. These effects tend to be more consistent with repeated dosing rather than with a single dose [1]. When performance benefits are observed, they typically involve modest improvements in fat oxidation during submaximal exercise, increased metabolic flexibility, or slight decreases in perceived effort [344]. In a double-blind, prophylactic supplementation trial, Kerksick and colleagues [84] demonstrated that 14 days of NAC (1800 mg/day) or EGCG (1800 mg/day) resulted in a slight reduction in subjective muscle soreness 24 h after eccentric knee extension exercise. However, neither supplement significantly affected strength loss, CK, LDH, inflammatory cell counts, or apoptotic signaling compared with the placebo. Kerksick et al. [85] in a follow-up study revealed that 14 days of NAC or EGCG did not reduce early increases in the MuRF1, UBE3B, or calpain genes after eccentric exercise in young males. Nevertheless, evidence for substantial improvements in time-trial or maximum performance remains inconsistent [342]. Some research suggests that exercise may enhance vascular function and reduce muscle soreness, possibly through the anti-inflammatory properties of catechins [1]; however, the findings are mixed [1]. No effects have been observed with products low in catechins, short-term dosing, or highly trained athletes whose bodies may already be adapted. Taking a high dose of EGCG immediately before resistance training can temporarily reduce blood flow or cause gastrointestinal issues in some individuals, highlighting the importance of proper timing and dosage [1]. In practice, athletes aiming for antioxidant support without impairing training adaptations may consume 400–800 mg/day of catechins (≈300–500 mg EGCG) from standardized extracts, or approximately 2–3 cups of brewed green tea daily [1]. Most protocols begin 7–14 days before major training sessions or competitions to foster the buildup of antioxidant enzymes. Immediate high-dose pre-workout supplements should be avoided, as they can disrupt redox signaling associated with training. Considering the potential risk of increased liver injury at high catechin doses (e.g. exceeding 800 mg EGCG daily) [345], along with an unclear safety threshold and limited evidence of exercise-related benefits, green tea-based catechin supplements may not be the best choice for athletes as antioxidants [345]. Green tea catechins, especially EGCG, can enhance antioxidant defenses, support vascular health, and influence metabolic responses important for endurance and intense training [1,340]. Although the performance improvements are generally modest and vary, regular intake of catechin-rich products can support recovery and maintain redox balance when integrated thoughtfully into an athlete’s diet [1,340].

### 11.13 Lutein and zeaxanthin

Lutein and zeaxanthin (LZX) are xanthophyll carotenoids primarily found in dark leafy green vegetables (i.e. kale, spinach) and egg yolks. Their hydroxylated β-ionone ring structures confer hydrophilic properties, enabling more efficient reactivity with oxidants. LZX is highly concentrated in the eye, where it acts as an antioxidant and a blue-light filter, particularly in the macula and retina, helping protect against age-related macular degeneration and cataracts [346,347]. Although lutein and zeaxanthin primarily accumulate in the macula and retina, numerous observational and clinical studies have shown that a higher dietary intake of L/Z is associated with a lower risk of cataracts [348,349]. These links seem to result from the systemic antioxidant effects of L/Z rather than their presence in the retina. Several meta-analyses and randomized clinical trials have demonstrated that higher intakes (>20 mg/day) of LZX over extended periods (up to 12 months) improve macular pigment optical density and visual function [350–361]. Furthermore, LZX deficiency may impair visual function in sports such as baseball, tennis, and target sports [362]. Yoshida and researchers [363] found that supplementation with 10 mg of lutein and 2 mg of zeaxanthin for 8 weeks improved hand‒eye coordination. Additional studies have reported enhanced visual processing speed, reaction time, and neural efficiency following LZX supplementation at 26–34 mg/day in healthy young adults [357,358]. These improvements could benefit athletes participating in visually demanding or high-speed environments.

There are also data suggesting that LZX supplementation can aid older athletes. For example, Murphy and colleagues [364] performed a cross-sectional analysis and longitudinal follow-up on an elderly population. They found that those with higher LZX concentrations had lower odds of frailty progression, better timed-up-and-go scores, and a more positive bone stiffness index. Additionally, Sahni and colleagues [365] found very similar findings; however, a positive association was observed directly between the serum LZX and muscle strength in older adults. Though these results are not derived from a supplementation or sports perspective, they still offer exciting potential for LZX to confer direct musculoskeletal effects, potentially improving exercise and sports performance. In summary, LZX are potent antioxidants that have shown efficacy in improving visual performance-related outcomes and may carry over into sports. Athletes may benefit from increased LZX intake to support visual acuity, cognitive processing, and potentially muscular function.

### 11.14 Omega-3 fatty acids

Polyunsaturated fatty acids (PUFAs), particularly omega-3 (*ω*-3) fatty acids such as eicosapentaenoic acid (EPA) and docosahexaenoic acid (DHA), play a central role in redox homeostasis and in cellular protection against ROS/RNS damage. Unlike saturated fats, PUFAs contain multiple (two or more) carbon‒carbon double bonds that strengthen membrane fluidity and stability, improving defenses against radicals [366–369]. Mechanistically, *ω*-3 s can (1) activate the Nrf2 pathway, enhancing endogenous antioxidant enzyme activity; (2) reduce ROS/RNS production via mitochondria-modulating mechanisms [370,371]; and (3) increase the amount of cardiolipin present, an inner mitochondrial membrane phospholipid that facilitates oxidative phosphorylation [372,373]. In addition, within mitochondria, DHA suppresses cytochrome C oxidase activity while simultaneously increasing the activity of manganese (SOD), a key mitochondrial antioxidant enzyme [374]. The consumption of diets rich in fish oil results in increased levels and activity of SOD, along with reduced lipid peroxidation, as indicated by lower concentrations of thiobarbituric acid-reactive substances (TBARS) [375]. In conclusion, *ω*-3 intake leads to marked antioxidant responses, primarily by restoring imbalances in endogenous antioxidant systems [374].

Several meta-analyses have demonstrated that supplementation with *ω*-3s can confer an antioxidant benefit across diseased and generally healthy populations. Heshmati and colleagues [376] assessed 39 trials with over 28,000 participants and found improvements in TAC and GPx activity along with reductions in MDA; however, the effects on nitric oxide, GSH, SOD, and CAT were equivocal. In another meta-analysis, *ω*-3 supplementation reduced CRP and increased TAC, but did not affect MDA or GSH [377]. Two other data further support improvements in TAC with prolonged *ω*-3 supplementation alongside mixed findings for nitric oxide, MDA, and endogenous antioxidant enzyme activity [366,376,377].

Evidence from randomized clinical trials has consistently demonstrated an antioxidant and anti-inflammatory benefit. For instance, Ghiasvand and colleagues [378] found that six weeks of 2000 mg/day of EPA, coupled with vitamin E supplementation, increased the production of anti-inflammatory cytokines, attenuated TNF-α release, and had additional effects on MDA. There are also clinical trials showing a decrease in serum and/or plasma MDA concentrations after prolonged *ω*-3 supplementation [379–384], with at least 1440 mg/day of a combined EPA and DHA formula from fish oil. Similarly, the results from clinical trials have shown marked improvements in TAC after at least 12 weeks of treatment with 1000 mg/day *ω*-3, including varying amounts of EPA and DHA [385–388]. In the context of exercise, *ω*-3 also has anti-inflammatory and antioxidative effects and can mitigate post-exercise muscle damage. For example, Barquilha and colleagues [389] found reductions in CRP, IL-6, CK, and LDH, and an increased GSH/GSSG ratio post-resistance training in physically active men and women who were supplemented with 260 mg/day of EPA and 202 mg/day of DHA for six weeks. Lee and colleagues [390] found that the consumption of fish oil (2100 mg/day of EPA and 720 mg/day of DHA) by older adults participating in a 12-week resistance training program decreased the IL-6, CRP, and TNF-α levels. Recently, VanDusseldorp and colleagues [391] demonstrated that fish oil supplementation of 6000 mg/day of *ω*-3 (2400 mg/day of EPA and 1800 mg/day of DHA) for approximately seven weeks resulted in attenuation of muscle damage, CK, and LDH 72 h post-eccentric exercise alongside improvements in subjective measures of muscle soreness in college-aged resistance-trained males. Although the results from exercise studies offer promise regarding the effects of *ω*-3s on muscle damage and oxidative stress markers concurrent with an exercise program, some research has yielded equivocal findings [392]. Nevertheless, supplementing with at least 1000 mg/day of *ω*-3s with a mixed EPA and DHA formula from fish oils or intake of an equivalent dose of *ω*-3 from food sources (i.e. cold water fish, flaxseed, and walnuts) yields antioxidative effects on a variety of populations (diseased, healthy, and athletic).

### 11.15 Pomegranate polyphenols and urolithin A

Pomegranate is a berry-like fruit (produced by *Punica granatum*) with approximately 52% of its total weight being edible, comprising approximately 78% juice and 22% seeds [393]. Typically, supplementation involves juice pressed from the whole fruit; however, pomegranate extracts in liquid or dry powder forms have gained popularity as concentrated sources of bioactive polyphenols [394]. Interest in pomegranate among athletes and active individuals has increased because of its high polyphenol content and associated cardiometabolic and anti-inflammatory benefits [394,395]. The consumption of pomegranate has been shown to decrease oxidative stress, inflammation, blood pressure, and blood lipids, and its polyphenols have been further researched for their effects on exercise performance and recovery [394,395]. Randomized controlled trials indicate that consuming high-polyphenol juice both before and after eccentric or resistance exercise results in faster strength recovery and reduced soreness [396,397]. Systematic reviews highlight that benefits are more consistent when juices contain more than 0.7 g of total polyphenols per 0.5 L, especially during exercise involving large muscle mass, and when juices are consumed at least 60 min prior to activity [394,395,398]. Conversely, studies in less trained groups or with lower-polyphenol products have shown no significant effects [395,399]. Typical supplementation involves 500–1000 mL of polyphenol-rich pomegranate juice (often divided into multiple doses) or standardized extracts, with at least 500–1000 mg administered daily [395,396,399–403]. These are typically initiated 3–7 days before intense training or events and maintained for 2–3 days afterward [395,396,399–403].

Urolithin A (UA) has garnered attention over the last decade for its promising applications in sports performance [404]. Derived from dietary precursors, such as pomegranates, UA is a naturally occurring metabolite that appears to play a crucial role in enhancing mitochondrial function by stimulating mitophagy and mitigating the age-related accumulation of dysfunctional mitochondria [405]. In terms of its health applications, UA has been assessed within the contexts of osteoarthritis [406], immunity [407], and neurodegenerative disease and disorders [408,409]. More recently, randomized controlled trials have shown much promise for UA in the application of sport [410–412]. For instance, Zhao et al. [412] assessed the impact of 8 weeks of 1 g/d of UA supplementation in a randomized, double-blind, placebo-controlled fashion among 20 resistance-trained male athletes. The UA group showed non-statistically significant increases in the 1RM bench press and squat (*Δ* = 3.00 ± 0.17 kg, *p* = 0.051; *Δ* = 1.35 ± 2.73 kg, *p* = 0.499, respectively). However, there were significant improvements in maximum voluntary isometric contraction and repetitions to failure (*Δ* = 36.10 ± 0.62 NM, *p* = 0.000; *Δ* = 2.00 ± 0.56, *p* = 0.001, respectively). Compared to the placebo group, the UA group also improved in maximum voluntary isometric contraction and repetitions to failure after 8 weeks (*Δ* = 43.50 ± 0.77 NM, *p* = 0.048; *Δ* = 2.00 ± 1.22, *p* = 0.011). Additionally, the UA group showed reductions in CRP (*Δ* = –0.79 ± 0.38 mg/L, *p* = 0.032) and SOD (*Δ* = −4.32 ± 0.90 U/mL, *p* = 0.041) compared with the placebo group. Whitfield et al. [411] demonstrated that 1000 mg/d of UA for 4 weeks during an altitude training camp (≈1700–2200 m) led to a reduction in ratings of perceived exertion during a 3000 m time trial (*p* = 0.02), as well as reduced indirect markers of post-exercise muscle damage, such as CK (*p* < 0.001). Furthermore, the UA group experienced a greater within-group increase in V̇O_2_max (5.4 ± 0.9%, 66.4 ± 0.8 to 70.0 ± 1.0 mL·kg^−1^·min^−1^, *p* = 0.009, *d* = −0.83) while only small changes were found in the placebo group (3.6 ± 1.3%, 66.4 ± 0.9 to 68.7 ± 1.0 mL·kg^−1^·min^−1^, *p* = 0.098, *d* = −0.54) [411]. Finally, UA upregulated mitochondrial pathways, decreased inflammatory pathways, and increased the expression of markers of mitophagy [411]. While UA supplementation is promising, more work is needed to understand the optimal dosing and duration.

### 11.16 Pycnogenol

Pycnogenol^®^ (PYC) is a procyanidin-rich extract derived from pine bark (*Pinus pinaster*) known for its potent antioxidant activity. PYC can neutralize ROS/RNS [413], upregulate endogenous enzyme activity [414], modulate NOS [415], inhibit lipid peroxidation [416], and exhibit metal-chelating properties [417]. Clinical studies have shown that daily PYC supplementation (100–200 mg) over 12 weeks can improve cardiometabolic health outcomes, including blood pressure, lipids, glucose, hemoglobin A1c, and biomarkers of oxidative stress and inflammation [418–423]. In addition to its metabolic health effects, PYC has functional benefits, such as reduced osteoarthritic pain and improved physical performance (WOMAC index) following supplementation at 150 mg/day for 90 days [424]. In a more athletic context, PYC has shown potential ergogenic effects, with studies reporting improvement in maximal aerobic capacity, aerobic power, time to fatigue, and recovery parameters in cyclists, triathletes, and military trainees at doses ranging from 60 to 200 mg/day for 4–8 weeks [425–429]. For instance, Hara and associates [426] found that 28 days of 60 mg/day of PYC supplementation led to enhanced maximal aerobic capacity, aerobic power, and caloric expenditure during cycling exercise. Furthermore, supplementation with PYC has also resulted in reductions in EIOS, improved performance on standardized physical ability tests (e.g. sit-ups and push-ups), and quicker total race times in triathlons [426,428]. Reports have also demonstrated PYC’s ability to mitigate exercise-induced cramps and muscle pain at doses ≥100 mg/day for 3–5 weeks [427,429,430]. Finally, PYC has demonstrated positive effects on cognitive function at doses ≥150 mg/day [431–435] in addition to enhanced working memory, pattern recognition, and mood, and reducing serum oxidative markers in both young and older adults, who typically use 100–150 mg/day for 12 weeks or longer [432,433,435–438]. In summary, PYC appears to support aspects of cardiometabolic health, cognitive and physical performance, and recovery in clinical and exercise settings. While data on PYC are limited, athletes may benefit from doses ≥100 mg/day across short- and long-term durations, and future studies should explore optimal supplement strategies.

### 11.17 Quercetin

Quercetin is a flavonoid found predominantly in citrus fruits and green leafy vegetables, with exceptionally high concentrations in capers, red onion, berries, red wine, buckwheat tea, and broccoli [439,440]. Its wide availability in whole foods makes regular intake a practical dietary strategy, with evidence suggesting benefits for metabolic health, including reductions in inflammation [441–443] and free radicals [439,444–447], as well as potential improvements in performance markers such as neuromuscular function and reduced soreness [258,259]. Mechanistically, quercetin is thought to activate the Nrf2 signaling pathway, thereby indirectly enhancing the expression of endogenous antioxidant enzymes [260]. However, findings on its ergogenic potential in athletic populations are limited and inconsistent. Some studies report improvements in outcomes, such as cycling performance, mitochondrial biogenesis, and body composition [448,449], while others report no significant differences compared to controls [450]. Most research has used doses of 400–600 mg, which are administered once to three times daily between meals for 7 days to 8 weeks [450]. Owing to quercetin's the poor bioavailability, its absorption can be enhanced by co-ingesting it with vitamin C or other vitamins and minerals, particularly when it is consumed after meals [450]. After absorption, quercetin undergoes extensive metabolism in the gut and liver, resulting in low levels of active quercetin in the bloodstream [451]. Differences in bioavailability – due to the form of quercetin in foods and individual enzyme variability – can further reduce its effectiveness. These metabolites may exhibit relatively weak antioxidant activity, potentially limiting the overall impact of quercetin on the body. While some evidence supports quercetin’s ability to reduce exercise-induced ROS and improve performance, further research in athletes is needed to confirm its efficacy.

### 11.18 Resveratrol

Resveratrol (3,5,4′-trihydroxystilbene) is a naturally occurring polyphenolic compound found in grapes, red wine, peanuts, and berries [452,453]. It possesses potent antioxidant and anti-inflammatory properties, scavenging ROS/RNS directly from the body. Resveratrol can also activate cellular pathways, such as SIRT1, AMPK, and Nrf2, which regulate mitochondrial biogenesis, energy metabolism, and redox balance [454,455]. Preclinical trials have consistently demonstrated that resveratrol can enhance exercise-induced adaptations, including improvements in mitochondrial function, endurance performance, and resistance to oxidative stress [456–458]. However, existing human data remain less supportive, reporting mixed or limited benefits. Some trials suggest that resveratrol may blunt training adaptations by interfering with redox-sensitive signaling [459–461]. For instance, Gliemann et al. [459] found that 250 mg/day of resveratrol in older men undergoing 8 weeks of high-intensity training attenuated gains in VO₂max and cardiovascular improvements. Similarly, Tsao et al. [462] found that although 480 mg/day resveratrol supplementation for 4 days reduced IL-6 levels in young athletes, it had no effect on other oxidative or performance markers. Because of these inconsistencies, resveratrol is not currently considered a strong candidate for use as a performance or recovery aid for healthy, active individuals or athletes, and further research is needed to determine the optimal dose. The heterogeneity in human responses underscores the need for carefully designed, randomized controlled trials, particularly among populations that stand to benefit most, such as older adults or individuals with physical limitations. For example, a pilot randomized controlled trial was conducted by Anton et al. [463] explored the efficacy of resveratrol combined with exercise in older adults, highlighting the importance of dose-dependent effects and potential improvements in functional outcomes. Future research should clarify optimal dosing, timing, and bioavailability issues, as well as interactions with physical training, to better define the role of resveratrol in supporting physical performance and health.

### 11.19 Selenium

Selenium is an essential trace mineral found in foods such as Brazil nuts, seafood, organ meats, and whole grains [464,465] that serves as a cofactor for various selenoproteins (e.g. GPx) involved in endogenous antioxidant defense and immune regulation. Selenium exerts many of its biological effects through its incorporation into GPxs, thioredoxin reductases, and selenoprotein P – key enzymes that mitigate oxidative stress by neutralizing hydrogen peroxide and lipid hydroperoxides [466]. Several studies have investigated the impact of selenium on redox balance and inflammation in physically active populations. For instance, trained cyclists who were supplemented with 240 µg/day of selenium-enriched yeast for 10 weeks showed increased GPx activity and reduced MDA levels compared with those in the placebo group [467]. In another study among active females, selenium supplementation (250 µg/day for 3 weeks) reduced oxidative stress and increased antioxidant levels, with the selenium-only group showing greater changes than the selenium + HIIT group [468]. In addition to its antioxidant effects, selenium supplementation has been shown to reduce serum IL-6 levels, though it does not affect CRP or TNF-α levels [469]. In addition, a selenium-containing supplement providing 50 µg/day of selenium over 21 days of intensive training supported physical performance and heart rate regulation in highly skilled tracks and field athletes [470]. Furthermore, in a recent study, individuals who were supplemented with selenium experienced faster decreases in IL-6 and CRP levels after high-intensity exercise, suggesting that selenium may enhance post-exercise recovery through its anti-inflammatory effects [471]. While selenium does not consistently enhance performance [472], supplementation can prevent deficiencies, reduce EIOS and mitochondrial changes, and may aid recovery – though evidence in trained athletes remains limited.

### 11.20 Spirulina

Spirulina is a blue‒green algae-derived, photosynthetic, gram-negative bacterium (*Arthrospira platensis*) that has been shown to have antioxidant, anti-inflammatory, anti-diabetic, anti-hypertensive, and lipid-lowering effects, with a safe dosage range of 3–10 g/day [473]. Currently, in the field of sport and exercise nutrition, spirulina supplementation is not well established for its positive ergogenic effects; however, several human trials may provide insight into its potential applications [474]. Koite and colleagues [475] found that supplementation with 2 g/L of Arthrospira extract (containing 1 g/L of phycocyanin and 0.5 g/L of Spirulysat, which includes proteins, amino acids, enzymes, vitamins, and minerals) for 12 weeks led to a reduction in urinary isoprostane levels. Bohorquez-Mordai et al. [476] conducted a systematic review and meta-analysis. They found that spirulina supplementation led to reductions in body mass, body fat percentage, and waist circumference among obese adults. Furthermore, Ismail and colleagues [477] found that spirulina (i.e. [500 × 2] or [500 × 4] mg for 60 days) resulted in reductions in the serum concentrations of MDA, lipid hydroperoxide, and total cholesterol, with increased GSH, vitamin C, SOD, glutathione-s-transferase activity, and glutathione. Recently, a study by Krokidas et al. [478] demonstrated that 42 mg/kg/day of spirulina for 15 days did not affect isometric muscle performance or exercise-induced muscle damage. More research is needed to better understand the impact of spirulina supplementation in athletic and trained populations.

### 11.21 Sulforaphane

Sulforaphane (SFN) is a naturally occurring isothiocyanate derived from cruciferous vegetables (i.e. broccoli sprouts), which contain high concentrations of its precursor, glucoraphanin. Upon chewing or processing, the enzyme myrosinase converts glucoraphanin to SFN (allowing it to be bioavailable) [479,480], which has drawn interest for its potent indirect antioxidant, anti-inflammatory, and cytoprotective properties [16,481–483]. Importantly, cooking deactivates myrosinase, and many broccoli supplements contain only glucoraphanin, reducing its efficacy unless it is paired with active myrosinase [484,485]. SFN primarily exerts its antioxidant effects [17,486,487], enabling a hormetic effect. This makes it distinct from traditional antioxidants (e.g. vitamins C and E), which can blunt training adaptations when used excessively [488]. SFN has also been shown to suppress pro-inflammatory cytokines (e.g. IL-6 and TNF-α), reduce NF-κB activation, and improve mitochondrial health [489–491]. These characteristics make SFN a compelling candidate for supporting recovery from intense training, reducing inflammation, and promoting metabolic adaptations in athletic populations [492–494]. In general, supplementation protocols have involved participants ingesting ≈20–40 mg/day of SFN derived from broccoli sprout extracts or supplements for 4–12 weeks [495,496]. Recent studies [492–494] suggest that SFN supplementation reduces post-exercise CK levels and shortens perceived recovery time without hindering hypertrophy or strength development [497]. Limited but promising data are available on the application of SFN in the context of exercise and recovery. For instance, Sato et al. [494] reported that 28 days of SFN supplementation (30 mg/day) in healthy men reduced plasma creatine kinase levels following an upper-body exercise-induced muscle-damage protocol compared with placebo. Similarly, Komine et al. [497] demonstrated that, compared with a control diet, 14 days of SFN supplementation (30 mg/day) before a muscle-damaging protocol reduced the expression of markers of soreness and oxidative stress. While more large-scale human trials are needed, the current findings support SFN’s use as a non-blunting, recovery-promoting antioxidant, especially when derived from myrosinase-active broccoli sprout extracts.

### 11.22 Tart cherry

Montmorency tart cherries (*Prunus cerasus L.*) are rich in phytonutrients, particularly flavonoids and other phenolic compounds, which have potent antioxidant and anti-inflammatory properties [498]. Emerging evidence suggests that the consumption of tart cherry juice or a powdered form may help lower cholesterol and triglyceride levels, improve blood pressure regulation and blood glucose control, enhance sleep quality and cognitive function, and support exercise recovery [498–501]. Given their high content of anthocyanins and polyphenols, tart cherries have garnered widespread interest among athletes and researchers for their potential to reduce muscle soreness, accelerate recovery, and support performance following high-intensity training [501,502].

To date, numerous studies have assessed the impact of tart cherry supplementation on resistance training [399,503–509], long-distance running [503,510–512], cycling [513,514], repeated sprints [513], and sport-specific training [515]. In terms of resistance training, as with some of the results seen with AST supplementation, the data demonstrate no unfavorable impact on key aspects of training adaptation [507]. For example, Jackman et al. [507] found that supplementation with 60 mL/day of Montmorency tart cherry concentrate for two weeks did not affect the anabolic response to resistance training. Notably, dietary antioxidants can negatively influence the natural training adaptation response [4]. Nevertheless, this does not appear to be the case with short-term tart cherry supplementation, at least at present.

Additionally, a 2020 meta-analysis of 10 studies revealed that consuming tart cherries for 7 days before and up to 1.5 h before an endurance exercise bout improved performance outcomes, such as time trials, time to exhaustion, and total work performed [516]. Wangdi and colleagues [517] later demonstrated that acute supplementation with tart cherry providing over 800 mg of polyphenols improved performance in a 15-km cycling time trial. The greatest improvements occurred when supplementation occurred 90 min before exercise (compared to 30 or 150 min), aligning with the greatest phenolic metabolite exposure during exercise. Notably, the performance benefits were more pronounced in trained individuals than in recreationally active individuals.

Tart cherry supplementation has also been shown to support post-exercise recovery [502]. Preloading tart cherry juice (i.e. two daily 30 mL servings) for several days before exercise can help preserve muscle function across various forms of physical activity [502,518]. A 2021 meta-analysis of 14 studies reported improvements in strength, reduced DOMS, and lower CRP and IL-6 levels, but no effect on CK or TNF-α [502]. Moreover, tart cherry supplementation has been shown to enhance the recovery of muscle function, reduce inflammation and oxidative stress markers, and alleviate pain following high-intensity running and cycling [510,511,519,520], with similar benefits observed after repeated sprint exercise [513,521,522]. Some studies have shown that tart cherry juice can reduce pain and promote strength recovery following heavy eccentric resistance exercise [505,508]. Supplementation with tart cherry powder (480 mg/day) has also been shown to reduce markers of muscle damage and inflammation and to decrease perceived soreness following intense strength and endurance exercise in trained individuals [504,512]. Finally, tart cherry supplementation may also increase circulating melatonin levels, thereby improving sleep duration and quality, further supporting recovery [523].

Given its potent antioxidant and anti-inflammatory properties, tart cherry supplementation appears to be a viable strategy for athletic populations to enhance exercise and sports performance while attenuating EIOS and inflammation, thereby promoting quicker recovery following high-intensity training [501–505,508,510–513,515,516,521,524]. Several tested supplementation dose and duration protocols have been reported in the literature, utilizing both powdered and juice tart cherry and varying timing strategies [502,516,525]. Generally, athletes considering tart cherry supplementation should aim for one of the following protocols: (1) a 200–500 mg capsule of tart cherry powder (containing 66–257 mg of anthocyanin), (2) 60–90 mL of cherry juice concentrate diluted with water (containing ≈550–820 mg of anthocyanin), or (3) 300–400 mL of cherry juice (containing ≈80 mg of anthocyanin) [502,516,525].

### 11.23 Vitamin E and vitamin C

Although the FDA recognizes Vitamin E (α-tocopherol) and Vitamin C (ascorbic acid) as antioxidants, the data overwhelmingly indicate mixed or limited performance and recovery-enhancing benefits when taken alone or in combination within the context of exercise and sport [3]. Vitamin E comprises lipid-soluble compounds that contain four tocopherols and four tocotrienols, with α-tocopherol being the most biologically active form. Tocopherols and tocotrienols are potent free radical scavengers. Once vitamin E neutralizes radicals (e.g. fatty acid peroxyl or tocopheroxyl radicals), vitamin C can regenerate it, allowing it to continue scavenging ROS/RNS [526]. Vitamin C is hydro-soluble and can also play a role in direct free radical scavenging. While vitamins E and C can be taken alone, Higgins and colleagues [3] conducted a systematic review of studies up to October 2020, concluding that vitamin E is most effective when combined with vitamin C, while vitamin E alone lacks strong support for enhancing sports performance.

Currently, the recommended daily allowance (RDA) for vitamin E is 15 mg (α-tocopherol) for both men and women, while the RDA for vitamin C is 90 mg for men and 75 mg for women. Notably, most athletes who are supplemented with dietary antioxidants are likely to meet or exceed these recommended intakes. However, it is important to note that the RDA may not necessarily be considered optimal for athletic populations [527]. Therefore, before considering dietary supplementation, it is recommended that individuals evaluate their diet and aim for a food-first approach, in which they consume foods rich in vitamins E and C to reap the benefits of these key antioxidants [3], as some data suggest that when one consumes substantially more than the RDA for these nutrients, the national training adaption response may be blunted [528]. For example, Ristow and associates [528] found that administering 400 IU/day of vitamin E and 1000 mg/day of vitamin C for 4 weeks to 39 untrained, healthy young men blunted PGC1-α induction and subsequent mitochondrial biogenesis, as well as key endogenous antioxidant enzymes. Therefore, if dietary supplementation is warranted, it is essential to consider the sport in which an individual is engaged and their training status.

To date, numerous studies [488,528–547] have investigated the effects of vitamin E, with or without vitamin C, on exercise and sports performance, as have studies [548–556] examining vitamin C alone. Following various supplementation protocols, most studies have reported no effect [488,529,531,533,534,536,540,542,543,545,549,553,554] on performance outcomes, with some reporting unfavorable outcomes (i.e. blunted training response) [528,530,551]. Furthermore, Nikolaidis et al. [557] conducted a review of 10 investigations on the ergogenic benefits of vitamin E and/or C supplementation during chronic exercise and subsequent adaptations, noting that only 2 of the 10 studies showed an ergogenic effect, while another 2 showed an ergolytic effect. In contrast, the remaining six studies reported no effect. Notably, in some cases, reports have also demonstrated no negative impact on the training response. For example, Zoppi et al. [546] assessed the effects of vitamin E (800 mg) and C (1000 mg) supplementation (divided into four daily doses) on performance, inflammation, and oxidative stress among male soccer players during a three-month pre-season training period. These findings demonstrated that the group supplementing with Vitamins E and C, compared to the placebo group, had reduced lipid peroxidation and muscle damage without hindering training adaptations. The most important consideration for individuals in regard to vitamins E and C is a well-balanced diet. For athletes following a standard American diet, supplementation with vitamins E or C may be appropriate, as dietary factors (e.g. a Western diet) can lead to relatively low vitamin stores, which can increase muscular fatigue and soreness. Therefore, supplementation can be promising in this context [558].

### 11.24 Zinc

Zinc, an essential trace element, plays a crucial role in endogenous antioxidant defense and redox homeostasis through both direct and indirect mechanisms, despite not being redox-active itself. One of the most well-established roles of zinc is serving as a cofactor for antioxidant enzymes, particularly copper–zinc-SOD, which is responsible for catalyzing the dismutation of O_2_^·–^ [559]. A study by Ribeiro and colleagues [560] revealed that 70 mg/day of zinc over 16 weeks in patients undergoing chemotherapy for colorectal cancer led to higher SOD activity during chemotherapy compared with placebo, demonstrating the indirect antioxidant role of zinc. Zinc is also a membrane stabilizer that protects cellular structures by maintaining sulfhydryl groups and displacing redox-active metals such as iron and copper, thereby reducing their pro-oxidant activity [561]. Zinc has been shown to induce the expression of metallothionein, a zinc-binding protein with potent free radical-scavenging capacity [562]. At the molecular level, zinc can activate the Nrf2 pathway, thereby enhancing the expression of endogenous antioxidant genes [563]. In the context of exercise and sports performance, zinc supplementation at 5 mg/kg/day for 4–6 days/week increases the levels of GSH, GPx, and SOD while reducing the level of MDA [564]. A meta-analysis of 21 RCTs involving 1321 participants further supports zinc’s ability to reduce biomarker concentrations of oxidative stress and inflammation, such as CRP, TNF-α, and MDA [565]. Collectively, these findings suggest that zinc can support endogenous antioxidant defense; however, more work is warranted among athletic populations, with exercise and sports performance outcomes at the forefront of assessment to better understand the application of zinc supplementation in sports.

### 11.25 Purported antioxidants: emerging or very limited evidence to support efficacy

Several nutrients and compounds are claimed to have antioxidant properties. However, it is important to recognize that most of these studies lack extensive pre-clinical data and have not yet been evaluated in randomized human clinical trials. Therefore, while acknowledging that these nutrients may offer some benefits to consumers, it is also clear that, like most dietary antioxidants with currently low efficacy evidence, these claimed antioxidants are not generally recommended and need further research. Table 3 lists these purported antioxidants and their respective mechanisms of action.

**Table 3.** Purported antioxidants with limited data.

Compound	Purported mechanism of action	Evidence strength
Beta-carotene	Scavenges singlet oxygen; precursor to vitamin A	Limited/preclinical
Melatonin	Regulates sleep; indirect antioxidant effects	Limited/preclinical
Lycopene	Quenches singlet oxygen; lipid peroxidation protection	Limited/preclinical
Carnosine	Buffers pH, some antioxidant properties	Limited/preclinical
Hesperidin	Citrus flavonoid; modulates antioxidant enzymes	Limited/preclinical
Silymarin (Milk thistle)	Flavonoid complex with hepatoprotective, antioxidant action	Limited/preclinical
Pterostilbene	Resveratrol analog; SIRT1 activator	Limited/preclinical
Gamma oryzanol	Plant sterol; antioxidant and anabolic claims	Limited/preclinical
Spinach thylakoid extract	Contains chloroplast-bound antioxidant enzymes (SOD, GPx)	Limited/human
GPLC (Glycine propionyl-L-carnitine)	Enhances nitric oxide; reduces lipid peroxidation (MDA)	Limited/human
SkQ (Mitochondria-targeted antioxidant)	Plastoquinone derivatives target mitochondrial ROS	Preclinical
Ketones	Promotes ketolysis; lowers oxidative stress markers (e.g. MDA)	Preclinical
Theaflavins	Polyphenols in black tea; scavenge ROS and modulate inflammatory pathways	Limited/preclinical
Apigenin	Flavonoid; Nrf2 activator, anti-inflammatory and neuroprotective	Preclinical
Melatonin	Endogenous antioxidant and sleep regulator	Limited/human
Lycopene	Carotenoid; quenches singlet oxygen	Limited/human
Olive fruit water (Hydroxytyrosol)	Phenolic compound; powerful free radical scavenger	Limited/human
PQQ (Pyrroloquinoline quinone)	Redox cofactor; supports mitochondrial biogenesis	Preclinical
Pterostilbene	Resveratrol analog; higher bioavailability; activates SIRT1, Nrf2	Limited/preclinical
Sulforaphane	Isothiocyanate; activates Nrf2, enhances endogenous defenses	Limited/human
Tyrosol	Phenolic from olive oil; antioxidant and anti-inflammatory	Preclinical
Watercress	Rich in isothiocyanates and polyphenols; indirect antioxidant	Limited/human
Ergothioneine	Sulfur-containing amino acid derivative; cytoprotective antioxidant	Preclinical

ROS, reactive oxygen species; SOD, superoxide dismutase; GPx, glutathione peroxidase; MDA, malondialdehyde; GPLC, glycine propionyl-L-carnitine; SkQ, plastoquinone antioxidant targeted to mitochondria; PQQ, pyrroloquinoline quinone; SIRT1, sirtuin 1 (a protein involved in cellular stress resistance and metabolic regulation); Nrf2, nuclear factor erythroid 2-related factor 2 (a transcription factor that regulates antioxidant defense pathways).

### 11.26 Section summary

There are numerous dietary antioxidants that athletes or performance professionals may consider leveraging in the context of exercise and sports; however, it is paramount that these individuals evaluate the data supporting these nutrients. There are a few “top-tier” dietary antioxidants with strong evidence supporting their efficacy for performance and recovery-related outcomes.

## 12 Special considerations

### 12.1 Elderly and masters athletes

Master’s athletes are generally defined as individuals over 35 or 40 years of age, but categories and age cutoffs vary by sport. Recently, increasing attention has been given to master’s athletics because this unique subgroup of adults is interested in optimizing their athletic performance while reducing the risk of disease burden, as they face physiological challenges associated with aging, including increased oxidative stress, inflammation, and anabolic resistance. While regular exercise can help maintain redox homeostasis [566–568], aging can independently increase ROS/RNS production [569,570], increasing the susceptibility of individuals to oxidative damage and, thus, poor health, performance, and recovery outcomes [568,571]. Given this, dietary antioxidant supplementation offers a potential solution to support these individuals in their respective training settings while also helping combat some of the natural processes of aging. Currently, research indicates that master’s athletes may not have adequate dietary antioxidant intake to support muscle function, immune health, and recovery [8]. Notably, although numerous studies have reported that redox and performance benefits from antioxidant supplementation, the overall evidence remains mixed. Some high-dose antioxidant studies have shown no effect, ergolytic effects, impaired performance, blunted training adaptations, or reduced endogenous antioxidant signaling [488]. As such, an antioxidant-rich food-first approach should be employed. Any complementary course of antioxidant supplementation should be tailored to specific scientific evidence, such as that presented herein. Interestingly, Guo and colleagues [572] recently conducted a systematic review of 26 studies encompassing 2819 master’s athletes and found that these individuals tend to consume more micronutrients than the general public. However, they may benefit from certain dietary antioxidant supplementation strategies, such as *ω*-3 fatty acids and polyphenols.

### 12.2 Tactical and occupational athletes

Tactical and occupational athletes are negatively affected by occupation-specific stressors, which can increase exposure to oxidative stress, inflammation, and increased disease risk [573]. There are several reasons for occupation-induced oxidative stress and inflammation, including intense physical exertion, psychological stress, shift work, poor dietary habits, traumatic experience, and other occupation-specific pressures (i.e. deployment and family separation) [574]. These findings highlight the key reasons why tactical or occupational athletes may consider supplementing with dietary antioxidants. However, to date, data are limited, and the results are mixed. For instance, Gonzalez and colleagues [189] assessed the impact of four weeks of AST (12 mg/day) supplementation among 15 career male firefighters and found that participants increased their ventilatory anaerobic threshold by 8.8%. In addition, they found that the firefighters showed lower physiological, oxidative, and inflammatory biomarker responses to simulated firefighting tasks after supplementation with AST. Another study by McAllister et al. [575] found that an acute dose of curcumin (i.e. a single dose of 1.5 g of curcumin/69 mg of curcuminoids) did not impact the oxidative stress response or physical performance during a simulated live burn, victim search, and rescue exercise. Regardless, tactical and occupational athletes need to identify pragmatic strategies to improve their performance, health, and recovery. While dietary antioxidants could benefit these populations, the data are limited.

### 12.3 Sex differences

It has been suggested that more than half of collegiate female athletes use traditional and non-traditional dietary supplements at least once a month to gain a competitive edge [576,577]. Previous reports have demonstrated sex-based differences in antioxidant status and responses to dietary antioxidant intake [577,578], with biological differences in hormonal regulation, body composition, and metabolism also likely affecting how men and women respond to increased free radical production and antioxidant intake [577,578]. Estrogen, for instance, exhibits intrinsic antioxidant properties and may enhance endogenous defense systems by modulating the expression and activity of key antioxidant enzymes, such as SOD and GPx [579]. It seems that females may produce less ROS and have higher antioxidant enzyme activity than males; however, this is not conclusive, as few studies have shown a uniform consensus or differences in several key antioxidant enzymes [578]. There is some evidence that men may derive greater protective benefits from dietary antioxidants, particularly as measured by the Composite Dietary Antioxidant Index (CDAI), compared to women [580]. However, it remains unclear whether these sex-based differences are consistent across women with different reproductive or menopausal statuses, as fluctuations in estrogen may substantially influence antioxidant defenses. Nevertheless, Duan et al. [580] assessed data from adults in the National Health and Nutrition Examination Survey (NHANES) cycles spanning 2001–2018 and found that the CDAI (calculated by summing the standardized intake values of six antioxidants: vitamin A, vitamin C, vitamin E, zinc, selenium, and total carotenoids) was negatively associated with all-cause mortality exclusively in men. The authors noted that estrogen may play a role by activating antioxidant pathways, thereby reducing the benefits of exogenous antioxidants in this context [578,579,581–583]. A 2023 ISSN position stand [584] highlights several dietary supplementation strategies for female athletes and women engaged in exercise (i.e. resistance or aerobic) training, including several dietary antioxidants, such as nitrates and CrM. These dietary supplements were found to be efficacious in the available literature for female populations, based on physiological theory and sex physiology [584].

### 12.4 Mitigating traumatic brain injury

Traumatic brain injuries (TBIs) are a major public health issue that can result in disability or premature mortality [585–588]. Daneshvar et al. [589] have suggested that an estimated 1.7 million individuals have sustained a TBI annually, with other reports indicating that over 69,000 TBI-related deaths occurred in the US in 2012 [585]. It is important to note that concerns about TBIs in sport have persisted for decades [589,590], with reports by Powell and Barber-Foss [591] suggesting that as many as 1219 mild TBIs were reported across various high school sports in 1999. Nowadays, the Centers for Disease Control have estimated that between 1.6 and 3.8 million concussions occur in sports and recreational activities annually [589]. This is also a major concern for tactical and occupational populations. For instance, approximately 500,000 US service members have sustained TBIs within the past 20 years [587]. Strack et al. [592] also noted that, in a sample of 1112 firefighters, 66% had reported at least one head injury in their lifetime, which was associated with higher levels of post-traumatic stress disorder and depression symptoms when compared to those who had not suffered a head injury. It is clear that TBIs are an issue, and these reports do not account for those individuals who suffer from TBI but do not seek medical attention [589]. Given that TBIs pose challenges and that sustaining a TBI induces an increased state of oxidative stress/redox imbalance, identifying avenues for the prevention and treatment of these injuries has led to interest in TBI-specific supplementation with select nutraceuticals and antioxidant compounds [586]. A 2024 review by Conti and colleagues [586] noted the specific supplementation recommendations for CrM (i.e. 4 × 5 g/day), *ω*-3 s (2–4 g/day with 2 g from DHA), choline (1–2 g/day), magnesium (400 md/day), NAC (4 g/day for 7 days followed by 3 g/day thereafter) and berry anthocyanins (250–400 mg/day), alongside food sources that should be considered for an individual needing to follow a nutritional and/or supplementation protocol for prevention and/or treatment for TBIs.

## 13 Conclusion and position of the International Society of Sports nutrition

Dietary antioxidants play a complex, context-dependent role in exercise and sport, striking a delicate balance between supporting recovery and potentially blunting physiological adaptations (e.g. with high-dose supplementation). While endogenous antioxidant systems are critical for maintaining redox balance, strategically increasing dietary antioxidants, preferably from whole foods, can enhance defense against excessive oxidative stress, especially during periods of high training load or inadequate recovery. However, the timing, dosage, and type of antioxidant compound must be carefully considered, as their effects vary according to training goals, individual physiology, and environmental demands. Ultimately, a personalized, evidence-based approach that acknowledges both the hormetic nature of exercise-induced oxidative stress and the complex biology of antioxidant action offers the greatest potential to optimize health and performance outcomes. Based on a comprehensive review and critical analysis of the literature regarding antioxidants conducted by experts in the field and selected members of the International Society of Sports Nutrition (ISSN), the following conclusions represent the official position of ISSN:

1. Redox balance exists on a spectrum, with mild oxidative eustress driving beneficial adaptations, while excessive distress impairing recovery and performance.
2. Exercise-induced ROS can support adaptation at moderate levels but may cause muscle damage, inflammation, and reduced endurance when excessive.
3. Antioxidants from endogenous systems and dietary sources protect cells by neutralizing free radicals and limiting oxidative damage to key biomolecules.
4. FDA labeling for “antioxidant” claims applies to nutrients with established RDIs and demonstrated antioxidant activity; this typically includes vitamins C and E, β-carotene (a source of vitamin A), selenium, zinc, copper, and manganese.
5. Several dietary compounds exhibit antioxidant activity, showing potential for both direct and indirect antioxidant effects. However, the strength of evidence varies, and their use should be tailored to align with specific performance or recovery goals.
6. Regular exercise improves endogenous antioxidant defenses and should be the primary strategy for enhancing redox capacity before considering supplementation.
7. Whole foods rich in flavonoids, polyphenols, carotenoids, vitamins, and minerals are the preferred antioxidant sources, with supplements used to fill dietary gaps.
8. Supplementation is best reserved for nutrient insufficiencies and deficiencies, inadequate dietary intake, or periods of high stress, while chronic high-dose use may blunt training adaptations.
9. Responses to supplementation vary by individual factors such as training status, baseline antioxidant capacity, demographics, diet, and injury risk, with some compounds offering cognitive, behavioral, or trauma-related benefits in specific populations.
10. Supplements such as creatine (i.e. 0.1 g/kg/day), omega-3 fatty acids (1000–6000 mg/day EPA+DHA for 6–12 weeks), tart cherry (480 mg powder or 60–90 mL juice/day for 7–14 days), and astaxanthin (4–12 mg/day for 4–12 weeks) rank among the top nutrients for their antioxidant effects, with moderate- to high-quality evidence supporting their use in recovery or performance without interfering with training adaptations. Most others exhibit weak or mixed data; therefore, selection should be tailored to training goals, biology, and the strength of the evidence.

## References

[1] Mason SA, Trewin AJ, Parker L, et al. Antioxidant supplements and endurance exercise: current evidence and mechanistic insights. Redox Biol. 2020 Aug;35:101471. doi: 10.1016/j.redox.2020.101471 32127289 PMC7284926

[2] Martinez-Ferran M, Sanchis-Gomar F, Lavie CJ, et al. Do antioxidant vitamins prevent exercise-induced muscle damage? A systematic review. Antioxidants. 2020;9(5):372. doi: 10.3390/antiox9050372 32365669 PMC7278664

[3] Higgins MR, Izadi A, Kaviani M. Antioxidants and exercise performance: with a focus on vitamin E and C supplementation. Int J Environ Res Public Health. 2020 Nov 15;17(22):8452. doi: 10.3390/ijerph17228452 33203106 PMC7697466

[4] Clemente-Suárez VJ, Bustamante-Sanchez Á, Mielgo-Ayuso J, et al. Antioxidants and sports performance. Nutrients. 2023 May 18;15(10):2371. doi: 10.3390/nu15102371 37242253 PMC10220679

[5] Sen CK. Oxidants and antioxidants in exercise. J Appl Physiol. 1995;79(3):675–686. doi: 10.1152/jappl.1995.79.3.675 8567503

[6] Powers SK, Jackson MJ. Exercise-induced oxidative stress: cellular mechanisms and impact on muscle force production. Physiol Rev. 2008 Oct;88(4):1243–1276. doi: 10.1152/physrev.00031.2007 18923182 PMC2909187

[7] Powers SK, Deminice R, Ozdemir M, et al. Exercise-induced oxidative stress: friend or foe? J Sport Health Sci. 2020 Sep;9(5):415–425. doi: 10.1016/j.jshs.2020.04.001 32380253 PMC7498668

[8] Maughan RJ, Burke LM, Dvorak J, et al. IOC consensus statement: dietary supplements and the high-performance athlete. Br J Sports Med. 2018 Apr;52(7):439–455. doi: 10.1136/bjsports-2018-099027 29540367 PMC5867441

[9] Powers SKaJ, Michael J. Endurance exercise and antioxidant supplementation: Sense or nonsense? – Part 2​. Sports Sci Exchange (Gatorade Sports Science Institute). 2014;27(138):1–6.

[10] Margaritelis NV, Paschalis V, Theodorou AA, et al. Antioxidants in personalized nutrition and exercise. Adv Nutr. 2018;9(6):813–823. 2018/11/01/. doi: 10.1093/advances/nmy052 30256898 PMC6247356

[11] Ismaeel A, Holmes M, Papoutsi E, et al. Resistance training, antioxidant status, and antioxidant supplementation. Int J Sport Nutr Exerc Metab. 2019 Sep 1;29(5):539–547. doi: 10.1123/ijsnem.2018-0339 30859847

[12] Kerksick CM, Wilborn CD, Roberts MD, et al. ISSN exercise & sports nutrition review update: research & recommendations. J Int Soc Sports Nutr. 2018;15(1):38. 2018/08/01. doi: 10.1186/s12970-018-0242-y 30068354 PMC6090881

[13] Clemente-Suárez VJ, Bustamante-Sanchez Á, Mielgo-Ayuso J, et al. Antioxidants and sports performance. Nutrients. 2023;15(10):2371. doi: 10.3390/nu15102371 37242253 PMC10220679

[14] Losada-Barreiro S, Sezgin-Bayindir Z, Paiva-Martins F, et al. Biochemistry of antioxidants: mechanisms and pharmaceutical applications. Biomedicines. 2022 Nov 25;10(12):3051. doi: 10.3390/biomedicines10123051 36551806 PMC9776363

[15] Halliwell B. Understanding mechanisms of antioxidant action in health and disease. Nat Rev Mol Cell Biol. 2024;25(1):13–33. 2024/01/01. doi: 10.1038/s41580-023-00645-4 37714962

[16] Dinkova-Kostova AT, Talalay P. Direct and indirect antioxidant properties of inducers of cytoprotective proteins. Mol Nutr Food Res. 2008 Jun;52 Suppl 1:S128–S138. 18327872 10.1002/mnfr.200700195

[17] Hayes JD, Dinkova-Kostova AT. The Nrf2 regulatory network provides an interface between redox and intermediary metabolism. Trends Biochem Sci. 2014;39(4):199–218. doi: 10.1016/j.tibs.2014.02.002 24647116

[18] Sies H. Editor. Oxidative stress. London: Academic Press; 1985.

[19] Sies H. Oxidative stress: concept and some practical aspects. Antioxidants. 2020;9(9):852. doi: 10.3390/antiox9090852 32927924 PMC7555448

[20] Sies H, Berndt C, Jones DP. Oxidative stress. Annu Rev Biochem. 2017;86(2017):715–748. 28441057 10.1146/annurev-biochem-061516-045037

[21] Powers SK, Radak Z, Ji LL. Exercise-induced oxidative stress: past, present and future. J Physiol. 2016;594(18):5081–5092. doi: 10.1113/JP270646 26893258 PMC5023699

[22] Jones DP. Redefining oxidative stress. Antioxid Redox Signaling. 2006;8(9-10):1865–1879. doi: 10.1089/ars.2006.8.1865 16987039

[23] Gomez-Cabrera MC, Carretero A, Millan-Domingo F, et al. Redox-related biomarkers in physical exercise. Redox Biol. 2021 Jun;42:101956. doi: 10.1016/j.redox.2021.101956 33811000 PMC8113051

[24] Davies KJ, Quintanilha AT, Brooks GA, et al. Free radicals and tissue damage produced by exercise. Biochem Biophys Res Commun. 1982 Aug 31;107(4):1198–1205. doi: 10.1016/S0006-291X(82)80124-1 6291524

[25] Mattson MP. Hormesis defined. Ageing Res Rev. 2008;7(1):1–7. doi: 10.1016/j.arr.2007.08.007 18162444 PMC2248601

[26] Radak Z, Chung HY, Goto S. Exercise and hormesis: oxidative stress-related adaptation for successful aging. Biogerontology. 2005;6:71–75. doi: 10.1007/s10522-004-7386-7 15834665

[27] Radak Z, Chung HY, Koltai E, et al. Exercise, oxidative stress and hormesis. Ageing Res Rev. 2008 Jan;7(1):34–42. doi: 10.1016/j.arr.2007.04.004 17869589

[28] Fisher-Wellman K, Bloomer RJ. Acute exercise and oxidative stress: a 30 year history. Dyn Med. 2009 Jan 13;8(1):1. doi: 10.1186/1476-5918-8-1 19144121 PMC2642810

[29] Reddy VP. Oxidative stress in health and disease. Biomedicines. 2023 Oct 29;11(11):2925. doi: 10.3390/biomedicines11112925 38001926 PMC10669448

[30] Zhao M, Veeranki SP, Magnussen CG, et al. Recommended physical activity and all cause and cause specific mortality in US adults: prospective cohort study. BMJ. 2020;370:m2031. doi: 10.1136/bmj.m2031 32611588 PMC7328465

[31] Lee DH, Rezende LFM, Joh H-K, et al. Long-term leisure-time physical activity intensity and all-cause and cause-specific mortality: a prospective cohort of US adults. Circulation. 2022;146(7):523–534. doi: 10.1161/CIRCULATIONAHA.121.058162 35876019 PMC9378548

[32] Paley CA, Johnson MI. Abdominal obesity and metabolic syndrome: exercise as medicine? BMC Sports Sci Med Rehabil. 2018;10:7. doi: 10.1186/s13102-018-0097-1 29755739 PMC5935926

[33] Ji LL. Exercise-induced modulation of antioxidant defense. Ann N Y Acad Sci. 2002 Apr;959:82–92. doi: 10.1111/j.1749-6632.2002.tb02085.x 11976188

[34] Powers SK, Goldstein E, Schrager M, et al. Exercise training and skeletal muscle antioxidant enzymes: an update. Antioxidants (Basel). 2022 Dec 25;12(1):39. doi: 10.3390/antiox12010039 36670901 PMC9854578

[35] Dillard C, Litov R, Savin W, et al. Effects of exercise, vitamin E, and ozone on pulmonary function and lipid peroxidation. J Appl Physiol. 1978;45(6):927–932. doi: 10.1152/jappl.1978.45.6.927 730598

[36] Lovlin R, Cottle W, Pyke I, et al. Are indices of free radical damage related to exercise intensity. Eur J Appl Physiol. 1987;56(3):313–316. doi: 10.1007/BF00690898 3569239

[37] Gohil K, Viguie C, Stanley WC, et al. Blood glutathione oxidation during human exercise. J Appl Physiol. 1988;64(1):115–119. doi: 10.1152/jappl.1988.64.1.115 3356628

[38] Fisher-Wellman K, Bloomer RJ. Acute exercise and oxidative stress: a 30 year history. Dyn Med. 2009;8:1–25. doi: 10.1186/1476-5918-8-1 19144121 PMC2642810

[39] Powers SK, Nelson WB, Hudson MB. Exercise-induced oxidative stress in humans: cause and consequences. Free Radic Biol Med. 2011 Sep 1;51(5):942–950. doi: 10.1016/j.freeradbiomed.2010.12.009 21167935

[40] Marzatico F, Pansarasa O, Bertorelli L, et al. Blood free radical antioxidant enzymes and lipid peroxides following long-distance and lactacidemic performances in highly trained aerobic and sprint athletes. J Sports Med Phys Fitness. 1997;37(4):235–239. 9509820

[41] McBride JM, Kraemer WJ, Triplett-McBride T, et al. Effect of resistance exercise on free radical production. Med Sci Sports Exerc. 1998 Jan;30(1):67–72. doi: 10.1097/00005768-199801000-00010 9475646

[42] Baker JS, Bailey DM, Hullin D, et al. Metabolic implications of resistive force selection for oxidative stress and markers of muscle damage during 30 s of high-intensity exercise. Eur J Appl Physiol. 2004;92:321–327. doi: 10.1007/s00421-004-1090-9 15098126

[43] Hudson MB, Hosick PA, McCaulley GO, et al. The effect of resistance exercise on humoral markers of oxidative stress. Med Sci Sports Exerc. 2008;40(3):542–548. doi: 10.1249/MSS.0b013e31815daf89 18379219

[44] Lee J, Goldfarb AH, Rescino MH, et al. Eccentric exercise effect on blood oxidative-stress markers and delayed onset of muscle soreness. Med Sci Sports Exerc. 2002 Mar;34(3):443–448. doi: 10.1097/00005768-200203000-00010 11880808

[45] Child R, Brown S, Day S, et al. Changes in indices of antioxidant status, lipid peroxidation and inflammation in human skeletal muscle after eccentric muscle actions. Clin Sci. 1999;96(1):105–115. doi: 10.1042/cs0960105 9857113

[46] Radák Z, Pucsok J, Mecseki S, et al. Muscle soreness-induced reduction in force generation is accompanied by increased nitric oxide content and DNA damage in human skeletal muscle. Free Radical Biol Med. 1999;26(7-8):1059–1063. doi: 10.1016/S0891-5849(98)00309-8 10232851

[47] Squillacioti G, Guglieri F, Colombi N, et al. Non-invasive measurement of exercise-induced oxidative stress in response to physical activity. A systematic review and meta-analysis. Antioxidants (Basel). 2021 Dec 17;10(12):2008. doi: 10.3390/antiox10122008 34943111 PMC8698343

[48] Boveris A, Chance B. The mitochondrial generation of hydrogen peroxide. General properties and effect of hyperbaric oxygen. Biochem J. 1973;134(3):707–716. doi: 10.1042/bj1340707 4749271 PMC1177867

[49] Powers SK, Deminice R, Ozdemir M, et al. Exercise-induced oxidative stress: friend or foe? J Sport Health Sci. 2020;9(5):415–425. 2020/09/01/. doi: 10.1016/j.jshs.2020.04.001 32380253 PMC7498668

[50] Vargas-Mendoza N, Angeles-Valencia M, Morales-González Á, et al. Oxidative stress, mitochondrial function and adaptation to exercise: new perspectives in nutrition. Life (Basel). 2021 Nov 22;11(11):1269. doi: 10.3390/life11111269 34833151 PMC8624755

[51] Ferreira LF, Laitano O. Regulation of NADPH oxidases in skeletal muscle. Free Radic Biol Med. 2016 Sep;98:18–28. doi: 10.1016/j.freeradbiomed.2016.05.011 27184955 PMC4975970

[52] Zhou Y, Zhang X, Baker JS, et al. Redox signaling and skeletal muscle adaptation during aerobic exercise. iScience. 2024 May 17;27(5):109643. doi: 10.1016/j.isci.2024.109643 38650987 PMC11033207

[53] Förstermann U, Sessa WC. Nitric oxide synthases: regulation and function. Eur Heart J. 2012 Apr;33(7):829–837. doi: 10.1093/eurheartj/ehr304 837a–837d. 21890489 PMC3345541

[54] Bouviere J, Fortunato RS, Dupuy C, et al. Exercise-stimulated ROS sensitive signaling pathways in skeletal muscle. Antioxidants (Basel). 2021 Mar 30;10(4):537. doi: 10.3390/antiox10040537 33808211 PMC8066165

[55] Ferreira LF, Reid MB. Muscle-derived ROS and thiol regulation in muscle fatigue. J Appl Physiol (1985). 2008 Mar;104(3):853–860. doi: 10.1152/japplphysiol.00953.2007 18006866

[56] Peternelj TT, Coombes JS. Antioxidant supplementation during exercise training: beneficial or detrimental? Sports Med. 2011 Dec 1;41(12):1043–1069. doi: 10.2165/11594400-000000000-00000 22060178

[57] McGinley C, Shafat A, Donnelly AE. Does antioxidant vitamin supplementation protect against muscle damage? Sports Med. 2009;39(12):1011–1032. doi: 10.2165/11317890-000000000-00000 19902983

[58] Stein JA, Farina EK, Karl JP, et al. Biomarkers of oxidative stress, diet and exercise distinguish soldiers selected and non-selected for special forces training. Metabolomics. 2023 Apr 11;19(4):39. doi: 10.1007/s11306-023-01998-9 37041398 PMC10090007

[59] Fiala O, Hanzlova M, Borska L, et al. Beyond physical exhaustion: understanding overtraining syndrome through the lens of molecular mechanisms and clinical manifestation. Sports Med Health Sci. 2025;7:237–248. doi: 10.1016/j.smhs.2025.01.006 40264836 PMC12010411

[60] Tanskanen M, Atalay M, Uusitalo A. Altered oxidative stress in overtrained athletes. J Sports Sci. 2010;28(3):309–317. doi: 10.1080/02640410903473844 20077275

[61] Nieman DC, Shanely RA, Luo B, et al. Metabolomics approach to assessing plasma 13- and 9-hydroxy-octadecadienoic acid and linoleic acid metabolite responses to 75-km cycling. Am J Physiol Regul Integr Comp Physiol. 2014 Jul 1;307(1):R68–74. doi: 10.1152/ajpregu.00092.2014 24760997

[62] Kargl CK, Gage CR, Forse JN, et al. Inflammatory and oxidant responses to arduous military training: associations with stress, sleep, and performance. Med Sci Sports Exerc. 2024 Dec 1;56(12):2315–2327. doi: 10.1249/MSS.0000000000003525 39160702

[63] Jówko E, Różański P, Tomczak A. Effects of a 36-h survival training with sleep deprivation on oxidative stress and muscle damage biomarkers in young healthy men. Int J Environ Res Public Health. 2018 Sep 20;15(10):2066. doi: 10.3390/ijerph15102066 30241324 PMC6211103

[64] Nieman DC, Henson DA, Smith LL, et al. Cytokine changes after a marathon race. J Appl Physiol (1985). 2001 Jul;91(1):109–114. doi: 10.1152/jappl.2001.91.1.109 11408420

[65] Rhind SG, Gannon GA, Shek PN, et al. Contribution of exertional hyperthermia to sympathoadrenal-mediated lymphocyte subset redistribution. J Appl Physiol (1985). 1999 Sep;87(3):1178–1185. doi: 10.1152/jappl.1999.87.3.1178 10484593

[66] Reid MB, Khawli FA, Moody MR. Reactive oxygen in skeletal muscle. Iii. contractility of unfatigued muscle. J Appl Physiol (1985). 1993 Sep;75(3):1081–1087. doi: 10.1152/jappl.1993.75.3.1081 8226515

[67] Peake JM, Markworth JF, Nosaka K, et al. Modulating exercise-induced hormesis: does less equal more? J Appl Physiol (1985). 2015 Aug 1;119(3):172–189. doi: 10.1152/japplphysiol.01055.2014 25977451

[68] Reid MB. Invited review: redox modulation of skeletal muscle contraction: what we know and what we Don't. J Appl Physiol (1985). 2001 Feb;90(2):724–731. doi: 10.1152/jappl.2001.90.2.724 11160074

[69] Close GL, Ashton T, McArdle A, et al. The emerging role of free radicals in delayed onset muscle soreness and contraction-induced muscle injury. Comp Biochem Physiol A Mol Integr Physiol. 2005 Nov;142(3):257–266. doi: 10.1016/j.cbpa.2005.08.005 16153865

[70] Lu Y, Wiltshire HD, Baker JS, et al. Effects of high intensity exercise on oxidative stress and antioxidant status in untrained humans: a systematic review. Biology (Basel). 2021 Dec 4;10(12):1272. doi: 10.3390/biology10121272 34943187 PMC8698973

[71] Di Meo S, Napolitano G, Venditti P. Mediators of physical activity protection against ROS-Linked skeletal muscle damage. Int J Mol Sci. 2019 Jun 20;20(12):3024. doi: 10.3390/ijms20123024 31226872 PMC6627449

[72] Cannon JG, St Pierre BA. Cytokines in exertion-induced skeletal muscle injury. Mol Cell Biochem. 1998 Feb;179(1-2):159–167. doi: 10.1023/A:1006828425418 9543358

[73] Powers SK, Ji LL, Kavazis AN, et al. Reactive oxygen species: impact on skeletal muscle. Compr Physiol. 2011 Apr;1(2):941–969. doi: 10.1002/j.2040-4603.2011.tb00348.x 23737208 PMC3893116

[74] Benedikter BJ, Weseler AR, Wouters EFM, et al. Redox-dependent thiol modifications: implications for the release of extracellular vesicles. Cell Mol Life Sci. 2018 Jul;75(13):2321–2337. doi: 10.1007/s00018-018-2806-z 29594387 PMC5986851

[75] Steinbacher P, Eckl P. Impact of oxidative stress on exercising skeletal muscle. Biomolecules. 2015 Apr 10;5(2):356–377. doi: 10.3390/biom5020356 25866921 PMC4496677

[76] Gill RM, O'Brien M, Young A, et al. Protein S-glutathionylation lowers superoxide/hydrogen peroxide release from skeletal muscle mitochondria through modification of complex I and inhibition of pyruvate uptake. PLoS One. 2018;13(2):e0192801. doi: 10.1371/journal.pone.0192801 29444156 PMC5812644

[77] Papanikolaou K, Veskoukis AS, Draganidis D, et al. Redox-dependent regulation of satellite cells following aseptic muscle trauma: implications for sports performance and nutrition. Free Radic Biol Med. 2020 Dec;161:125–138. 33039652 10.1016/j.freeradbiomed.2020.10.001

[78] Mason SA, Morrison D, McConell GK, et al. Muscle redox signalling pathways in exercise. Role of antioxidants. Free Radic Biol Med. 2016 Sep;98:29–45. doi: 10.1016/j.freeradbiomed.2016.02.022 26912034

[79] Close GL, Ashton T, Cable T, et al. Eccentric exercise, isokinetic muscle torque and delayed onset muscle soreness: the role of reactive oxygen species. Eur J Appl Physiol. 2004 May;91(5-6):615–621. doi: 10.1007/s00421-003-1012-2 14685863

[80] Maughan RJ, Donnelly AE, Gleeson M, et al. Delayed-onset muscle damage and lipid peroxidation in man after a downhill run. Muscle Nerve. 1989 Apr;12(4):332–336. doi: 10.1002/mus.880120412 2770784

[81] Kanda K, Sugama K, Hayashida H, et al. Eccentric exercise-induced delayed-onset muscle soreness and changes in markers of muscle damage and inflammation. Exerc Immunol Rev. 2013;19:72–85. 23977721

[82] Kerksick C, Willoughby D. The antioxidant role of glutathione and N-Acetyl-Cysteine supplements and exercise-induced oxidative stress. J Int Soc Sports Nutr. 2005;2(2):38. 2005/12/01. doi: 10.1186/1550-2783-2-2-38 18500954 PMC2129149

[83] Kerksick C, Taylor L, Harvey A, et al. Gender-related differences in muscle injury, oxidative stress, and apoptosis. Med Sci Sports Exerc. 2008 Oct;40(10):1772–1780. doi: 10.1249/MSS.0b013e31817d1cce 18799987

[84] Kerksick CM, Kreider RB, Willoughby DS. Intramuscular adaptations to eccentric exercise and antioxidant supplementation. Amino Acids. 2010 Jun;39(1):219–232. doi: 10.1007/s00726-009-0432-7 19967420

[85] Kerksick CM, Roberts MD, Dalbo VJ, et al. Changes in skeletal muscle proteolytic gene expression after prophylactic supplementation of EGCG and NAC and eccentric damage. Food Chem Toxicol. 2013 Nov;61:47–52. doi: 10.1016/j.fct.2013.01.026 23376779

[86] Kerksick CM, Willoughby D, Kouretas D, et al. Intramuscular responses with muscle damaging exercise and the interplay between multiple intracellular networks: a human perspective. Food Chem Toxicol. 2013 Nov;61:136–143. doi: 10.1016/j.fct.2013.04.029 23624378

[87] McKenzie DC. Markers of excessive exercise. Can J Appl Physiol. 1999 Feb;24(1):66–73. doi: 10.1139/h99-007 9916182

[88] Palazzetti S, Richard MJ, Favier A, et al. Overloaded training increases exercise-induced oxidative stress and damage. Can J Appl Physiol. 2003 Aug;28(4):588–604. doi: 10.1139/h03-045 12904636

[89] Cheng AJ, Jude B, Lanner JT. Intramuscular mechanisms of overtraining. Redox Biol. 2020 Aug;35:101480. doi: 10.1016/j.redox.2020.101480 32179050 PMC7284919

[90] Finaud J, Lac G, Filaire E. Oxidative stress: relationship with exercise and training. Sports Med. 2006;36(4):327–358. doi: 10.2165/00007256-200636040-00004 16573358

[91] Powers SK, Lennon SL. Analysis of cellular responses to free radicals: focus on exercise and skeletal muscle. Proc Nutr Soc. 1999 Nov;58(4):1025–1033. doi: 10.1017/S0029665199001342 10817171

[92] Tanskanen M, Atalay M, Uusitalo A. Altered oxidative stress in overtrained athletes. J Sports Sci. 2010 Feb;28(3):309–317. doi: 10.1080/02640410903473844 20077275

[93] Powers SK, Talbert EE, Adhihetty PJ. Reactive oxygen and nitrogen species as intracellular signals in skeletal muscle. J Physiol. 2011 May 1;589(Pt 9):2129–2138. doi: 10.1113/jphysiol.2010.201327 21224240 PMC3098692

[94] Ji LL, Kang C, Zhang Y. Exercise-induced hormesis and skeletal muscle health. Free Radic Biol Med. 2016 Sep;98:113–122. doi: 10.1016/j.freeradbiomed.2016.02.025 26916558

[95] Gomez-Cabrera MC, Domenech E, Viña J. Moderate exercise is an antioxidant: upregulation of antioxidant genes by training. Free Radic Biol Med. 2008 Jan 15;44(2):126–131. doi: 10.1016/j.freeradbiomed.2007.02.001 18191748

[96] Kramer HF, Goodyear LJ. Exercise, MAPK, and NF-κB signaling in skeletal muscle. J Appl Physiol. 2007;103(1):388–395. doi: 10.1152/japplphysiol.00085.2007 17303713

[97] Barbieri E, Sestili P. Reactive oxygen species in skeletal muscle signaling. J Signal Transduct. 2012;2012:982794. doi: 10.1155/2012/982794 22175016 PMC3235811

[98] Beckendorf L, Linke WA. Emerging importance of oxidative stress in regulating striated muscle elasticity. J Muscle Res Cell Motil. 2015 Feb;36(1):25–36. doi: 10.1007/s10974-014-9392-y 25373878 PMC4352196

[99] Fulle S, Protasi F, Di Tano G, et al. The contribution of reactive oxygen species to sarcopenia and muscle ageing. Exp Gerontol. 2004 Jan;39(1):17–24. doi: 10.1016/j.exger.2003.09.012 14724060

[100] Powers SK, Morton AB, Ahn B, et al. Redox control of skeletal muscle atrophy. Free Radic Biol Med. 2016 Sep;98:208–217. doi: 10.1016/j.freeradbiomed.2016.02.021 26912035 PMC5006677

[101] Watson TA, Callister R, Taylor RD, et al. Antioxidant restriction and oxidative stress in short-duration exhaustive exercise. Med Sci Sports Exerc. 2005 Jan;37(1):63–71. doi: 10.1249/01.MSS.0000150016.46508.A1 15632670

[102] Berzosa C, Cebrián I, Fuentes-Broto L, et al. Acute exercise increases plasma total antioxidant status and antioxidant enzyme activities in untrained men. J Biomed Biotechnol. 2011;2011:540458. doi: 10.1155/2011/540458 21436993 PMC3062968

[103] Georgakouli K, Manthou E, Fatouros IG, et al. Effects of acute exercise on liver function and blood redox status in heavy drinkers. Exp Ther Med. 2015 Dec;10(6):2015–2022. doi: 10.3892/etm.2015.2792 26668589 PMC4665762

[104] Finaud J, Scislowski V, Lac G, et al. Antioxidant status and oxidative stress in professional rugby players: evolution throughout a season. Int J Sports Med. 2006 Feb;27(2):87–93. doi: 10.1055/s-2005-837489 16475052

[105] Margonis K, Fatouros IG, Jamurtas AZ, et al. Oxidative stress biomarkers responses to physical overtraining: implications for diagnosis. Free Radic Biol Med. 2007 Sep 15;43(6):901–910. doi: 10.1016/j.freeradbiomed.2007.05.022 17697935

[106] Benedetti S, Catalani S, Peda F, et al. Impact of the 24-h ultramarathon race on homocysteine, oxidized low-density lipoprotein, and paraoxonase 1 levels in professional runners. PLoS One. 2018;13(2):e0192392. doi: 10.1371/journal.pone.0192392 29394290 PMC5796729

[107] Guerrero C, Collado-Boira E, Martinez-Navarro I, et al. Impact of plasma oxidative stress markers on post-race recovery in ultramarathon runners: a sex and age perspective overview. Antioxidants (Basel). 2021 Feb 27;10(3):355. doi: 10.3390/antiox10030355 33673404 PMC7996940

[108] He F, Li J, Liu Z, et al. Redox mechanism of reactive oxygen species in exercise. Front Physiol. 2016;7:486. doi: 10.3389/fphys.2016.00486 27872595 PMC5097959

[109] Nielsen HG, Hagberg IA, Lyberg T. Marathon running leads to partial exhaustion of ROS-generating capacity in leukocytes. Med Sci Sports Exerc. 2004 Jan;36(1):68–73. doi: 10.1249/01.MSS.0000106168.12113.95 14707770

[110] Turner JE, Hodges NJ, Bosch JA, et al. Prolonged depletion of antioxidant capacity after ultraendurance exercise. Med Sci Sports Exerc. 2011 Sep;43(9):1770–1776. doi: 10.1249/MSS.0b013e31821240bb 22534974

[111] Lewis NA, Howatson G, Morton K, et al. Alterations in redox homeostasis in the elite endurance athlete. Sports Med. 2015 Mar;45(3):379–409. doi: 10.1007/s40279-014-0276-5 25319354

[112] Knez WL, Jenkins DG, Coombes JS. Oxidative stress in half and full ironman triathletes. Med Sci Sports Exerc. 2007 Feb;39(2):283–288. doi: 10.1249/01.mss.0000246999.09718.0c 17277592

[113] Thirupathi A, Pinho RA, Ugbolue UC, et al. Effect of running exercise on oxidative stress biomarkers: a systematic review. Front Physiol. 2020;11:610112. doi: 10.3389/fphys.2020.610112 33551836 PMC7854914

[114] Niki E. Interaction of ascorbate and alpha-tocopherol. Ann N Y Acad Sci. 1987;498:186–199. doi: 10.1111/j.1749-6632.1987.tb23761.x 3304060

[115] Carr AC, Zhu B-Z, Frei B. Potential antiatherogenic mechanisms of ascorbate (Vitamin C) and α-Tocopherol (Vitamin E). Circ Res. 2000;87(5):349–354. 2000/09/01. doi: 10.1161/01.RES.87.5.349 10969031

[116] Nagaoka S-i, Kakiuchi T, Ohara K, et al. Kinetics of the reaction by which natural vitamin E is regenerated by vitamin C. Chem Phys Lipids. 2007;146(1):26–32. doi: 10.1016/j.chemphyslip.2006.12.001 17270164

[117] Carr A, Frei B. Does vitamin C act as a pro‐oxidant under physiological conditions? FASEB J. 1999;13(9):1007–1024. doi: 10.1096/fasebj.13.9.1007 10336883

[118] Alugoju P, Swamy K, Anthikapalli Nva VKD, et al. Health benefits of astaxanthin against age-related diseases of multiple organs: a comprehensive review. Crit Rev Food Sci Nutr. 2022 Jun 16; 1–66. 10.1080/10408398.2022.2084600 35708049

[119] Cao Y, Yang L, Qiao X, et al. Crit Rev Food Sci Nutr. 2021 Sep 28. 1–27.

[120] Chang MX, Xiong F. Astaxanthin and its effects in inflammatory responses and inflammation-associated diseases: recent advances and future directions. Molecules. 2020 Nov 16;25(22):5342. doi: 10.3390/molecules25225342 33207669 PMC7696511

[121] Kohandel Z, Farkhondeh T, Aschner M, et al. Anti-inflammatory action of astaxanthin and its use in the treatment of various diseases. Biomed Pharmacother. 2022 Jan;145:112179. doi: 10.1016/j.biopha.2021.112179 34736076

[122] Pereira CPM, Souza ACR, Vasconcelos AR, et al. Antioxidant and anti‑inflammatory mechanisms of action of astaxanthin in cardiovascular diseases (Review). Int J Mol Med. 2021 Jan;47(1):37–48. doi: 10.3892/ijmm.2020.4783 PMC7723678 33155666

[123] Kalyanaraman B. NAC, NAC, Knockin' on Heaven's door: interpreting the mechanism of action of N-acetylcysteine in tumor and immune cells. Redox Biol. 2022 Nov;57:102497. doi: 10.1016/j.redox.2022.102497 36242913 PMC9563555

[124] Rodrigues E, Mariutti LRB, Mercadante AZ. Scavenging capacity of marine carotenoids against reactive oxygen and nitrogen species in a membrane-mimicking system. Mar Drugs. 2012 Aug;10(8):1784–1798. doi: 10.3390/md10081784 23015774 PMC3447262

[125] Kurutas EB. The importance of antioxidants which play the role in cellular response against oxidative/nitrosative stress: current state. Nutr J. 2016 Jul 25;15(1):71. doi: 10.1186/s12937-016-0186-5 27456681 PMC4960740

[126] Sansone RA, Sansone LA. Getting a knack for NAC: N-Acetyl-Cysteine. Innov Clin Neurosci. 2011 Jan;8(1):10–14. PMC3036554 21311702

[127] Liebler DC, Kling DS, Reed DJ. Antioxidant protection of phospholipid bilayers by alpha-tocopherol. Control of alpha-tocopherol status and lipid peroxidation by ascorbic acid and glutathione. J Biol Chem. 1986 Sep 15;261(26):12114–12119. doi: 10.1016/S0021-9258(18)67210-2 3745181

[128] Ambati RR, Phang SM, Ravi S, et al. Astaxanthin: sources, extraction, stability, biological activities and its commercial applications – a review. Mar Drugs. 2014 Jan 7;12(1):128–152. doi: 10.3390/md12010128 24402174 PMC3917265

[129] Chandimali N, Bak SG, Park EH, et al. Free radicals and their impact on health and antioxidant defenses: a review. Cell Death Discov. 2025;11(1):19. doi: 10.1038/s41420-024-02278-8. 2025/01/24. 39856066 PMC11760946

[130] Ferran M, Sanchis-Gomar F, Lavie CJ, et al. Do antioxidant vitamins prevent exercise-induced muscle damage? A systematic review. Antioxidants. 2020;9(5):372. doi: 10.3390/antiox9050372 32365669 PMC7278664

[131] Margaritelis NV, Kyparos A, Paschalis V, et al. Reductive stress after exercise: the issue of redox individuality. Redox Biol. 2014;2:520–528. doi: 10.1016/j.redox.2014.02.003 24634834 PMC3953955

[132] Margaritelis NV, Paschalis V, Theodorou AA, et al. Antioxidant supplementation, redox deficiencies and exercise performance: a falsification design. Free Radical Biol Med. 2020;158:44–52. 2020/10/01/. doi: 10.1016/j.freeradbiomed.2020.06.029 32682929

[133] Margaritelis NV, Theodorou AA, Paschalis V, et al. Experimental verification of regression to the mean in redox biology: differential responses to exercise. Free Radical Res. 2016;50(11):1237–1244. doi: 10.1080/10715762.2016.1233330 27596985

[134] Margaritelis NV, Theodorou AA, Paschalis V, et al. Adaptations to endurance training depend on exercise‐induced oxidative stress: exploiting redox interindividual variability. Acta Physiol. 2018;222(2):e12898. doi: 10.1111/apha.12898 28544643

[135] Margaritelis NV, Nastos GG, Vasileiadou O, et al. Inter-individual variability in redox and performance responses after antioxidant supplementation: a randomized double blind crossover study. Acta Physiol (Oxford). 2023 Aug;238(4):e14017. doi: 10.1111/apha.14017 37401190

[136] Carlsen MH, Halvorsen BL, Holte K, et al. The total antioxidant content of more than 3100 foods, beverages, spices, herbs and supplements used worldwide. Nutr J. 2010 Jan 22;9:3. doi: 10.1186/1475-2891-9-3 20096093 PMC2841576

[137] Rahaman MM, Hossain R, Herrera-Bravo J, et al. Natural antioxidants from some fruits, seeds, foods, natural products, and associated health benefits: an update. Food Sci Nutr. 2023 Apr;11(4):1657–1670. doi: 10.1002/fsn3.3217 37051367 PMC10084981

[138] Ullah A, Munir S, Badshah SL, et al. Important flavonoids and their role as a therapeutic agent. Molecules. 2020 Nov 11;25(22):5243. doi: 10.3390/molecules25225243 33187049 PMC7697716

[139] Garthe I, Maughan RJ. Athletes and supplements: prevalence and perspectives. Int J Sport Nutr Exerc Metab. 2018 Mar 1;28(2):126–138. doi: 10.1123/ijsnem.2017-0429 29580114

[140] Daher J, Mallick M, El Khoury D. Prevalence of dietary supplement use among athletes worldwide: a scoping review. Nutrients. 2022 Oct 3;14(19):4109. doi: 10.3390/nu14194109 36235761 PMC9570738

[141] Weight LM, Myburgh KH, Noakes TD. Vitamin and mineral supplementation: effect on the running performance of trained athletes. AJCN. 1988 Feb;47(2):192–195. doi: 10.1093/ajcn/47.2.192 3341247

[142] Solmonson A, DeBerardinis RJ. Lipoic acid metabolism and mitochondrial redox regulation. J Biol Chem. 2018 May 18;293(20):7522–7530. doi: 10.1074/jbc.TM117.000259 29191830 PMC5961061

[143] Ou P, Tritschler HJ, Wolff SP. Thioctic (lipoic) acid: a therapeutic metal-chelating antioxidant? Biochem Pharmacol. 1995;50(1):123–126. doi: 10.1016/0006-2952(95)00116-H 7605337

[144] Suh JH, Moreau R, Heath S-HD, et al. Dietary supplementation with (R)-α-lipoic acid reverses the age-related accumulation of iron and depletion of antioxidants in the rat cerebral cortex. Redox Rep. 2005;10(1):52–60. doi: 10.1179/135100005X21624 15829111

[145] Hager K, Kenklies M, McAfoose J, et al. A-lipoic acid as a new treatment option for Alzheimer’s disease a 48 months follow-up analysis. J Neural Transm. 2007;72:189–193. 10.1007/978-3-211-73574-9_24 17982894

[146] Baziar N, Nasli-Esfahani E, Djafarian K, et al. The beneficial effects of alpha lipoic acid supplementation on Lp‐PLA2 mass and its distribution between HDL and apob‐containing lipoproteins in type 2 diabetic patients: a randomized, double‐blind, placebo‐controlled trial. Oxid Med Cell Longev. 2020;2020(1):5850865. doi: 10.1155/2020/5850865 32256955 PMC7085885

[147] Mendoza-Núñez VM, García-Martínez BI, Rosado-Pérez J, et al. The effect of 600 mg alpha‐lipoic acid supplementation on oxidative stress, inflammation, and RAGE in older adults with type 2 diabetes mellitus. Oxid Med Cell Longev. 2019;2019(1):3276958. doi: 10.1155/2019/3276958 31285784 PMC6594273

[148] Rahimlou M, Asadi M, Jahromi NB, et al. Alpha-lipoic acid (ALA) supplementation effect on glycemic and inflammatory biomarkers: a systematic review and meta-analysis. Clin Nutr ESPEN. 2019;32:16–28. doi: 10.1016/j.clnesp.2019.03.015 31221283

[149] Bobe G, Michels AJ, Zhang W-J, et al. A randomized controlled trial of long-term (R)-α-Lipoic acid supplementation promotes weight loss in overweight or obese adults without altering baseline elevated plasma triglyceride concentrations. J Nutr. 2020;150(9):2336–2345. 2020/09/01/. doi: 10.1093/jn/nxaa203 32692358 PMC7540064

[150] Isenmann E, Trittel L, Diel P. The effects of alpha lipoic acid on muscle strength recovery after a single and a short-term chronic supplementation - a study in healthy well-trained individuals after intensive resistance and endurance training. J Int Soc Sports Nutr. 2020 Dec 1;17(1):61. doi: 10.1186/s12970-020-00389-y 33261642 PMC7708149

[151] Morawin B, Turowski D, Naczk M, et al. The combination of α-lipoic acid intake with eccentric exercise modulates erythropoietin release. Biol Sport. 2014 Aug;31(3):179–185. doi: 10.5604/20831862.1111435 25177095 PMC4135061

[152] Fogarty MC, Devito G, Hughes CM, et al. Effects of α-lipoic acid on mtDNA damage after isolated muscle contractions. Med Sci Sports Exerc. 2013 Aug;45(8):1469–1477. doi: 10.1249/MSS.0b013e31828bf31e 23470303

[153] Bonilla DA, Moreno Y, Gho C, et al. Effects of ashwagandha (*Withania somnifera*) on physical performance: systematic review and Bayesian meta-analysis. J Funct Morphol Kinesiol. 2021;6(1):20. doi: 10.3390/jfmk6010020 33670194 PMC8006238

[154] Sprengel M, Laskowski R, Jost Z. Withania somnifera (Ashwagandha) supplementation: a review of its mechanisms, health benefits, and role in sports performance. Nutr Metabol. 2025;22(1):9. 2025/02/05. doi: 10.1186/s12986-025-00902-7 PMC11800443 39910586

[155] Bonilla DA, Moreno Y, Gho C, et al. Effects of ashwagandha (*Withania somnifera*) on physical performance: systematic review and Bayesian meta-analysis. J Funct Morphol Kinesiol. 2021 Feb 11;6(1):20. doi: 10.3390/jfmk6010020 33670194 PMC8006238

[156] Mikulska P, Malinowska M, Ignacyk M, et al. Ashwagandha (*Withania somnifera*)-Current research on the health-promoting activities: a narrative review. Pharmaceutics. 2023 Mar 24;15(4):1057. doi: 10.3390/pharmaceutics15041057 37111543 PMC10147008

[157] Baitharu I, Jain V, Deep SN, et al. Withanolide A prevents neurodegeneration by modulating hippocampal glutathione biosynthesis during hypoxia. PLoS One. 2014;9(10):e105311. doi: 10.1371/journal.pone.0105311 25310001 PMC4195593

[158] Wankhede S, Langade D, Joshi K, et al. Examining the effect of withania somnifera supplementation on muscle strength and recovery: a randomized controlled trial. J Int Soc Sports Nutr. 2015;12:1–11. doi: 10.1186/s12970-015-0104-9 26609282 PMC4658772

[159] Verma N, Gupta SK, Patil S, et al. Effects of ashwagandha (*Withania somnifera*) standardized root extract on physical endurance and VO (2max) in healthy adults performing resistance training: an eight-week, prospective, randomized, double-blind, placebo-controlled study. F1000Res. 2023;12:335. doi: 10.12688/f1000research.130932.1 38988644 PMC11234080

[160] Ziegenfuss TN, Kedia AW, Sandrock JE, et al. Effects of an aqueous extract of withania somnifera on strength training adaptations and recovery: the STAR trial. Nutrients. 2018;10(11):1807. doi: 10.3390/nu10111807 30463324 PMC6266766

[161] Raut AA, Rege NN, Tadvi FM, et al. Exploratory study to evaluate tolerability, safety, and activity of ashwagandha (*Withania somnifera*) in healthy volunteers. J Ayurveda Integr Med. 2012;3(3):111. doi: 10.4103/0975-9476.100168 23125505 PMC3487234

[162] Fassett RG, Coombes JS. Astaxanthin, oxidative stress, inflammation and cardiovascular disease. Future Cardiol. 2009 Jul;5(4):333–342. doi: 10.2217/fca.09.19 19656058

[163] Li J, Guo C, Wu J. Astaxanthin in liver health and disease: a potential therapeutic agent. Drug Des Devel Ther. 2020;14:2275–2285. doi: 10.2147/DDDT.S230749 PMC7293384 32606597

[164] Pashkow FJ, Watumull DG, Campbell CL. Astaxanthin: a novel potential treatment for oxidative stress and inflammation in cardiovascular disease. Am J Cardiol. 2008 May 22;101(10a):58d–68d. doi: 10.1016/j.amjcard.2008.02.010 18474276

[165] Ma B, Lu J, Kang T, et al. Astaxanthin supplementation mildly reduced oxidative stress and inflammation biomarkers: a systematic review and meta-analysis of randomized controlled trials. Nutr Res. 2022 Mar;99:40–50. doi: 10.1016/j.nutres.2021.09.005 35091276

[166] Chan K-c, Chen S-c, Chen P-c. Astaxanthin attenuated thrombotic risk factors in type 2 diabetic patients. J Funct Foods. 2019;53:22–27. 2019/02/01/. doi: 10.1016/j.jff.2018.12.012

[167] Baralic I, Andjelkovic M, Djordjevic B, et al. Effect of astaxanthin supplementation on salivary IgA, oxidative stress, and inflammation in young soccer players. Evid based Complement Alternat Med. 2015;2015:1–9. doi: 10.1155/2015/783761 PMC4488551 26167194

[168] Baralic I, Djordjevic B, Dikic N, et al. Effect of astaxanthin supplementation on paraoxonase 1 activities and oxidative stress status in young soccer players. Phytother Res. 2013 Oct;27(10):1536–1542. doi: 10.1002/ptr.4898 23192897

[169] Chen J-T, Kotani K. Effects of astaxanthin on liver and leukocyte parameters in healthy climacteric women: preliminary data. J Med Food. 2017;20(7):724–725. doi: 10.1089/jmf.2016.3819 28692413

[170] Choi HD, Kim JH, Chang MJ, et al. Effects of astaxanthin on oxidative stress in overweight and obese adults. Phytother Res. 2011;25(12):1813–1818. doi: 10.1002/ptr.3494 21480416

[171] Coombes JS, Sharman JE, Fassett RG. Astaxanthin has no effect on arterial stiffness, oxidative stress, or inflammation in renal transplant recipients: a randomized controlled trial (the XANTHIN trial). Am J Clin Nutr. 2016;103(1):283–289. doi: 10.3945/ajcn.115.115477 26675778

[172] Djordjevic B, Baralic I, Kotur-Stevuljevic J, et al. Effect of astaxanthin supplementation on muscle damage and oxidative stress markers in elite young soccer players. J Sports Med Phys Fitness. 2012 Aug;52(4):382–392. 22828460

[173] Karppi, Rissanen, Nyyssönen, et al. Effects of astaxanthin supplementation on lipid peroxidation. Int J Vitam Nutr Res. 2007;77(1):3–11. doi: 10.1024/0300-9831.77.1.3 17685090

[174] Kim JH, Chang MJ, Choi HD, et al. Protective effects of haematococcus astaxanthin on oxidative stress in healthy smokers. J Med Food. 2011 Nov;14(11):1469–1475. doi: 10.1089/jmf.2011.1626 21883001

[175] Klinkenberg LJ, Res PT, Haenen GR, et al. Effect of antioxidant supplementation on exercise-induced cardiac troponin release in cyclists: a randomized trial. PLoS One. 2013;8(11):e79280. doi: 10.1371/journal.pone.0079280 24260184 PMC3834092

[176] McAllister MJ, Mettler JA, Patek K, et al. Astaxanthin supplementation increases glutathione concentrations but does not impact fat oxidation during exercise in active young men. Int J Sport Nutr Exerc Metab. 2022 Jan 1;32(1):8–15. doi: 10.1123/ijsnem.2021-0138 34611051

[177] Nakagawa K, Kiko T, Miyazawa T, et al. Antioxidant effect of astaxanthin on phospholipid peroxidation in human erythrocytes. Br J Nutr. 2011 Jun;105(11):1563–1571. doi: 10.1017/S0007114510005398 21276280

[178] Park JS, Chyun JH, Kim YK, et al. Astaxanthin decreased oxidative stress and inflammation and enhanced immune response in humans. Nutr Metabol. 2010;7:1–10. 10.1186/1743-7075-7-18 PMC2845588 20205737

[179] Yoshida H, Yanai H, Ito K, et al. Administration of natural astaxanthin increases serum HDL-cholesterol and adiponectin in subjects with mild hyperlipidemia. Atherosclerosis. 2010 Apr;209(2):520–523. doi: 10.1016/j.atherosclerosis.2009.10.012 19892350

[180] Wika AA, Reason KW, Green JM, et al. Astaxanthin reduces heart rate and carbohydrate oxidation rates during exercise in overweight individuals. Int J Exerc Sci. 2023;16(2):252–266. 37114194 10.70252/FPGA5839 PMC10124739

[181] Okamoto T, Kase M, Yokoi M, et al. Reversible Horner's syndrome and dysthyroid ocular myopathy associated with Hashimoto's disease. Jpn J Ophthalmol. 2003 Nov–Dec;47(6):587–590. doi: 10.1016/S0021-5155(03)00145-X 14636849

[182] Hashimoto H, Arai K, Hayashi S, et al. Effects of astaxanthin on antioxidation in human aqueous humor. J Clin Biochem Nutr. 2013 Jul;53(1):1–7. doi: 10.3164/jcbn.13-6 23874063 PMC3705160

[183] Miki W. Biological functions and activities of animal carotenoids. Pure Appl Chem. 1991;63(1):141–146. doi: 10.1351/pac199163010141

[184] Visioli F, Artaria C. Astaxanthin in cardiovascular health and disease: mechanisms of action, therapeutic merits, and knowledge gaps. Food Funct. 2017 Jan 25;8(1):39–63. doi: 10.1039/C6FO01721E 27924978

[185] Brown DR, Gough LA, Deb SK, et al. Astaxanthin in exercise metabolism, performance and recovery: a review. Front Nutr. 2017;4:76. doi: 10.3389/fnut.2017.00076 29404334 PMC5778137

[186] Baralic I, Andjelkovic M, Djordjevic B, et al. Effect of astaxanthin supplementation on salivary IgA, oxidative stress, and inflammation in young soccer players. Evid based Complement Alternat Med. 2015;2015:783761. doi: 10.1155/2015/783761 26167194 PMC4488551

[187] Barker GA, Parten AL, Lara DA, et al. Astaxanthin supplementation reduces subjective markers of muscle soreness following eccentric exercise in resistance-trained men. Muscles. 2023;2(2):228–237. doi: 10.3390/muscles2020017 40757570 PMC12225492

[188] Fleischmann C, Horowitz M, Yanovich R, et al. Asthaxanthin improves aerobic exercise recovery without affecting heat tolerance in humans. Front Sports Act Living. 2019;1:17. doi: 10.3389/fspor.2019.00017 33344941 PMC7739736

[189] Gonzalez DE, Dickerson BL, Johnson SE, et al. Impact of astaxanthin supplementation on markers of cardiometabolic health and tactical performance among firefighters. J Int Soc Sports Nutr. 2024 Dec;21(1):2427751. doi: 10.1080/15502783.2024.2427751 39568140 PMC11583326

[190] Sawaki K, Yoshigi H, Aoki K, et al. Sports performance benefits from taking natural astaxanthin characterized by visual acuity and muscle fatigue improvements in humans. J Clin Therapeut Med. 2002;18(9):73–88.

[191] Malmsten C, Lignell A. Dietary supplementation with astaxanthin-rich algal meal improves strength Endurance–A double blind placebo controlled study on Male Students. Carotenoid Sci. 2008;13:20–22.

[192] Brown DR, Warner AR, Deb SK, et al. The effect of astaxanthin supplementation on performance and fat oxidation during a 40 km cycling time trial. J Sci Med Sport. 2021;24(1):92–97. doi: 10.1016/j.jsams.2020.06.017 32660833

[193] McAllister MJ, Mettler JA, Patek K, et al. Astaxanthin supplementation increases glutathione concentrations but does not impact fat oxidation during exercise in active young men. Int J Sport Nutr Exerc Metab. 2022;32(1):8–15. 01 Jan. 2022. doi: 10.1123/ijsnem.2021-0138​​​​​​ 34611051

[194] Res PT, Cermak NM, Stinkens R, et al. Astaxanthin supplementation does not augment fat use or improve endurance performance. Med Sci Sports Exerc. 2013;45(6):1158–1165. doi: 10.1249/MSS.0b013e31827fddc4 23274592

[195] Liu SZ, Ali AS, Campbell MD, et al. Building strength, endurance, and mobility using an astaxanthin formulation with functional training in elderly. J Cachexia Sarcopenia Muscle. 2018 Oct;9(5):826–833. doi: 10.1002/jcsm.12318 30259703 PMC6204600

[196] Behrens M, Gube M, Chaabene H, et al. Fatigue and human performance: an updated framework. Sports Med. 2023 Jan;53(1):7–31. doi: 10.1007/s40279-022-01748-2 PMC9807493 36258141

[197] Clifford T, Howatson G, West DJ, et al. The potential benefits of red beetroot supplementation in health and disease. Nutrients. 2015 Apr 14;7(4):2801–2822. doi: 10.3390/nu7042801 25875121 PMC4425174

[198] Bailey SJ, Winyard P, Vanhatalo A, et al. Dietary nitrate supplementation reduces the O2 cost of low-intensity exercise and enhances tolerance to high-intensity exercise in humans. J Appl Physiol (1985). 2009 Oct;107(4):1144–1155. doi: 10.1152/japplphysiol.00722.2009 19661447

[199] Domínguez R, Maté-Muñoz JL, Cuenca E, et al. Effects of beetroot juice supplementation on intermittent high-intensity exercise efforts. J Int Soc Sports Nutr. 2018;15(1):2. 2018/01/05. doi: 10.1186/s12970-017-0204-9 29311764 PMC5756374

[200] Nirmal S, Olatunde OO, Medhe S, et al. Betalains alleviate exercise-induced oxidative stress, inflammation, and fatigue and improve sports performance: an update on recent advancement. Curr Nutr Rep. 2023 Dec;12(4):778–787. doi: 10.1007/s13668-023-00500-0 37824059

[201] Zoughaib WS, Fry MJ, Singhal A, et al. Beetroot juice supplementation and exercise performance: is there more to the story than just nitrate? Front Nutr. 2024;11:1347242. doi: 10.3389/fnut.2024.1347242 38445207 PMC10912565

[202] Clifford T, Allerton DM, Brown MA, et al. Minimal muscle damage after a marathon and no influence of beetroot juice on inflammation and recovery. Appl Physiol Nutr Metab. 2017 Mar;42(3):263–270. doi: 10.1139/apnm-2016-0525 28165768

[203] Clifford T, Bell O, West DJ, et al. The effects of beetroot juice supplementation on indices of muscle damage following eccentric exercise. Eur J Appl Physiol. 2016 Feb;116(2):353–362. doi: 10.1007/s00421-015-3290-x 26537365

[204] Clifford T, Berntzen B, Davison GW, et al. Effects of beetroot juice on recovery of muscle function and performance between bouts of repeated sprint exercise. Nutrients. 2016 Aug 18;8(8):506. doi: 10.3390/nu8080506 27548212 PMC4997419

[205] Clifford T, Howatson G, West DJ, et al. Beetroot juice is more beneficial than sodium nitrate for attenuating muscle pain after strenuous eccentric-bias exercise. Appl Physiol Nutr Metab. 2017 Nov;42(11):1185–1191. doi: 10.1139/apnm-2017-0238 28719765

[206] Kozłowska L, Mizera O, Gromadzińska J, et al. Changes in oxidative stress, inflammation, and muscle damage markers following diet and beetroot juice supplementation in elite fencers. Antioxidants (Basel). 2020 Jul 1;9(7):571. doi: 10.3390/antiox9070571 32630279 PMC7402086

[207] Daab W, Bouzid MA, Lajri M, et al. Chronic beetroot juice supplementation accelerates recovery kinetics following simulated match play in soccer players. J Am Coll Nutr. 2021 Jan;40(1):61–69. doi: 10.1080/07315724.2020.1735571 32125249

[208] Vilar E, Collado-Boira E, Guerrero C, et al. Is there a role of beetroot consumption on the recovery of oxidative status and muscle damage in ultra-endurance runners? Nutrients. 2024 Feb 21;16(5):583. doi: 10.3390/nu16050583 38474711 PMC10934908

[209] Jones L, Bailey SJ, Rowland SN, et al. The effect of nitrate-rich beetroot juice on markers of exercise-induced muscle damage: a systematic review and meta-analysis of human intervention trials. J Diet Suppl. 2022;19(6):749–771. doi: 10.1080/19390211.2021.1939472 34151694

[210] Rojano-Ortega D, Peña Amaro J, Berral-Aguilar AJ, et al. Effects of beetroot supplementation on recovery after exercise-induced muscle damage: a systematic review. Sports Health. 2022 Jul–Aug;14(4):556–565. doi: 10.1177/19417381211036412 34399653 PMC9214898

[211] Tanabe Y, Fujii N, Suzuki K. Dietary supplementation for attenuating exercise-induced muscle damage and delayed-onset muscle soreness in humans. Nutrients. 2021 Dec 24;14(1):70. doi: 10.3390/nu14010070 35010943 PMC8746365

[212] Cook MD, Willems MET. Dietary anthocyanins: a review of the exercise performance effects and related physiological responses. Int J Sport Nutr Exerc Metab. 2019;29(3):322–330. doi: 10.1123/ijsnem.2018-0088 30160565

[213] de Pascual-Teresa S, Moreno DA, García-Viguera C. Flavanols and anthocyanins in cardiovascular health: a review of current evidence. Int J Mol Sci. 2010;11(4):1679–1703. doi: 10.3390/ijms11041679 20480037 PMC2871133

[214] Tang X, Shen T, Jiang X, et al. Purified anthocyanins from bilberry and black currant attenuate hepatic mitochondrial dysfunction and steatohepatitis in mice with methionine and choline deficiency. J Agric Food Chem. 2015;63(2):552–561. doi: 10.1021/jf504926n 25536170

[215] Braakhuis AJ, Somerville VX, Hurst RD. The effect of New Zealand blackcurrant on sport performance and related biomarkers: a systematic review and meta-analysis. J Int Soc Sports Nutr. 2020 May 27;17(1):25. doi: 10.1186/s12970-020-00354-9 32460873 PMC7251677

[216] Cook MD, Myers SD, Blacker SD, et al. New Zealand blackcurrant extract improves cycling performance and fat oxidation in cyclists. Eur J Appl Physiol. 2015 Nov;115(11):2357–2365. doi: 10.1007/s00421-015-3215-8 26175097

[217] Godwin C, Cook MD, Willems ME. Effect of New Zealand blackcurrant extract on performance during the running based anaerobic sprint test in trained youth and recreationally active male football players. Sports. 2017;5(3):69. doi: 10.3390/sports5030069 29910429 PMC5968969

[218] Hurst RD, Lyall KA, Roberts JM, et al. Consumption of an anthocyanin-rich extract made from New Zealand blackcurrants prior to exercise may assist recovery from oxidative stress and maintains circulating neutrophil function: a pilot study. Front Nutr. 2019;6:73. doi: 10.3389/fnut.2019.00073 31192216 PMC6548855

[219] Hurst RD, Lyall KA, Wells RW, et al. Daily consumption of an anthocyanin-rich extract made from New Zealand blackcurrants for 5 weeks supports exercise recovery through the management of oxidative stress and inflammation: a randomized placebo controlled pilot study. Front Nutr. 2020;7:16. doi: 10.3389/fnut.2020.00016 32175326 PMC7056812

[220] Lomiwes D, Ha B, Ngametua N, et al. Timed consumption of a New Zealand blackcurrant juice support positive affective responses during a self-motivated moderate walking exercise in healthy sedentary adults. J Int Soc Sports Nutr. 2019;16(1):33. doi: 10.1186/s12970-019-0300-0 31375128 PMC6679481

[221] Murphy CA, Cook MD, Willems ME. Effect of New Zealand blackcurrant extract on repeated cycling time trial performance. Sports. 2017;5(2):25. doi: 10.3390/sports5020025 29910385 PMC5968979

[222] Perkins IC, Blacker SD, Willems MET. Individual responses to repeated dosing with anthocyanin-rich New Zealand blackcurrant extract during high-intensity intermittent treadmill running in active males. Nutrients. 2024 Dec 10;16(24):4253. doi: 10.3390/nu16244253 39770875 PMC11677273

[223] Perkins IC, Vine SA, Blacker SD, et al. New Zealand blackcurrant extract improves high-intensity intermittent running. Int J Sport Nutr Exerc Metab. 2015;25(5):487–493. doi: 10.1123/ijsnem.2015-0020 25812064

[224] Potter JA, Hodgson CI, Broadhurst M, et al. Effects of New Zealand blackcurrant extract on sport climbing performance. Eur J Appl Physiol. 2020;120(1):67–75. doi: 10.1007/s00421-019-04226-2 31515632

[225] Strauss JA, Willems MET, Shepherd SO. New Zealand blackcurrant extract enhances fat oxidation during prolonged cycling in endurance-trained females. Eur J Appl Physiol. 2018 Jun;118(6):1265–1272. doi: 10.1007/s00421-018-3858-3 29619595 PMC5966492

[226] Willems ME, Cousins L, Williams D, et al. Beneficial effects of New Zealand blackcurrant extract on maximal sprint speed during the loughborough intermittent shuttle test. Sports. 2016;4(3):42. doi: 10.3390/sports4030042 29910290 PMC5968887

[227] Willems ME, Myers SD, Blacker SD, et al. CurraNZ blackcurrant improves cycling performance and recovery in trained endurance athletes. J Int Soc Sports Nutr. 2014;11(sup1):P14. doi: 10.1186/1550-2783-11-S1-P14

[228] Cook MD, Myers SD, Blacker SD, et al. New Zealand blackcurrant extract improves cycling performance and fat oxidation in cyclists. Eur J Appl Physiol. 2015;115(11):2357–2365. doi: 10.1007/s00421-015-3215-8 26175097

[229] Christensen PM, Shirai Y, Ritz C, et al. Caffeine and bicarbonate for speed. A meta-analysis of legal supplements potential for improving intense endurance exercise performance. Front Physiol. 2017;8:240. doi: 10.3389/fphys.2017.00240 28536531 PMC5422435

[230] Holt RR, Lazarus SA, Sullards MC, et al. Procyanidin dimer B2 [epicatechin-(4β-8)-epicatechin] in human plasma after the consumption of a flavanol-rich cocoa. Am J Clin Nutr. 2002;76(4):798–804. doi: 10.1093/ajcn/76.4.798 12324293

[231] Decroix L, Soares DD, Meeusen R, et al. Cocoa flavanol supplementation and exercise: a systematic review. Sports Med. 2018 Apr;48(4):867–892. doi: 10.1007/s40279-017-0849-1 29299877

[232] Efsa Panel on Dietetic P. NaAN. scientific opinion on the substantiation of a health claim related to cocoa flavanols and maintenance of normal endothelium dependent vasodilation. EFSA J. 2012;10(7):2809. 10.2903/j.efsa.2012.2809 PMC13093062 42016115

[233] Efsa Panel on Dietetic P. NaAN. scientific opinion on the modification of the authorisation of a health claim related to cocoa flavanols and maintenance of normal endothelium dependent vasodilation. EFSA J. 2014;12(5):3654. 10.2903/j.efsa.2014.3654 PMC13159857 42125362

[234] González-Garrido JA, García-Sánchez JR, Garrido-Llanos S, et al. An association of cocoa consumption with improved physical fitness and decreased muscle damage and oxidative stress in athletes. J Sports Med Phys Fitness. 2015;57(4):441–447. 26632851 10.23736/S0022-4707.16.06032-1

[235] Wiswedel I, Hirsch D, Kropf S, et al. Flavanol-rich cocoa drink lowers plasma F2-isoprostane concentrations in humans. Free Radical Biol Med. 2004;37(3):411–421. doi: 10.1016/j.freeradbiomed.2004.05.013 15223075

[236] Allgrove J, Farrell E, Gleeson M, et al. Regular dark chocolate consumption’s reduction of oxidative stress and increase of free-fatty-acid mobilization in response to prolonged cycling. Int J Sport Nutr Exerc Metab. 2011;21(2):113–123. doi: 10.1123/ijsnem.21.2.113 21558573

[237] Davison G, Callister R, Williamson G, et al. The effect of acute pre-exercise dark chocolate consumption on plasma antioxidant status, oxidative stress and immunoendocrine responses to prolonged exercise. Eur J Nutr. 2012;51(1):69–79. doi: 10.1007/s00394-011-0193-4 21465244

[238] Fraga CG, Actis-Goretta L, Ottaviani JI, et al. Regular consumption of a flavanol‐rich chocolate can improve oxidant stress in young soccer players. J Immunol Res. 2005;12(1):11–17. doi: 10.1080/10446670410001722159 PMC2270716 15712594

[239] Patel RK, Brouner J, Spendiff O. Dark chocolate supplementation reduces the oxygen cost of moderate intensity cycling. J Int Soc Sports Nutr. 2015;12(1):47. doi: 10.1186/s12970-015-0106-7 26674253 PMC4678700

[240] Stellingwerff T, Godin J-P, Chou CJ, et al. The effect of acute dark chocolate consumption on carbohydrate metabolism and performance during rest and exercise. Appl Physiol Nutr Metabol. 2014;39(2):173–182. doi: 10.1139/apnm-2013-0152 24476473

[241] Taub PR, Ramirez-Sanchez I, Patel M, et al. Beneficial effects of dark chocolate on exercise capacity in sedentary subjects: underlying mechanisms. A double blind, randomized, placebo controlled trial. Food Function. 2016;7(9):3686–3693. doi: 10.1039/C6FO00611F 27491778 PMC5025384

[242] Suárez-Rivero JM, Pastor-Maldonado CJ, Povea-Cabello S, et al. Coenzyme Q(10) analogues: benefits and challenges for therapeutics. Antioxidants (Basel). 2021 Feb 4;10(2):236. doi: 10.3390/antiox10020236 33557229 PMC7913973

[243] Crane F, Sun I, Sun E. The essential functions of coenzyme Q. Clin Invest. 1993;71:S55–S59. doi: 10.1007/BF00226841 8241706

[244] Ernster L, Dallner G. Biochemical, physiological and medical aspects of ubiquinone function. Biochim Biophys Acta (BBA)-Mol Basis Disease. 1995;1271(1):195–204. doi: 10.1016/0925-4439(95)00028-3 7599208

[245] Sue-Ling CB, Abel WM, Sue-Ling K. Coenzyme Q10 as adjunctive therapy for cardiovascular disease and hypertension: a systematic review. J Nutr. 2022;152(7):1666–1674. 35348726 10.1093/jn/nxac079

[246] Zhang S-y, Yang K-l, Zeng L-t, et al. Effectiveness of coenzyme Q10 supplementation for type 2 diabetes mellitus: a systematic review and meta-analysis. Int J Endocrinol. 2018;2018(1):6484839. doi: 10.1155/2018/6484839 30305810 PMC6165589

[247] Zhang T, He Q, Xiu H, et al. Efficacy and safety of coenzyme Q10 supplementation in the treatment of polycystic ovary syndrome: a systematic review and meta-analysis. Reprod Sci. 2023;30(4):1033–1048. doi: 10.1007/s43032-022-01038-2 35941510

[248] Braun B, Clarkson PM, Freedson PS, et al. Effects of coenzyme Q10 supplementation on exercise performance, VO2max, and lipid peroxidation in trained cyclists. Int J Sport Nutr Exerc Metab. 1991;1(4):353–365. doi: 10.1123/ijsn.1.4.353 1844568

[249] Diaz-Castro J, Mira-Rufino PJ, Moreno-Fernandez J, et al. Ubiquinol supplementation modulates energy metabolism and bone turnover during high intensity exercise. Food Function. 2020;11(9):7523–7531. doi: 10.1039/D0FO01147A 32797125

[250] Diaz-Castro J, Moreno-Fernandez J, Chirosa I, et al. Beneficial effect of ubiquinol on hematological and inflammatory signaling during exercise. Nutrients. 2020;12(2):424. doi: 10.3390/nu12020424 32041223 PMC7071169

[251] Ho C-C, Chang P-S, Chen H-W, et al. Ubiquinone supplementation with 300 mg on glycemic control and antioxidant status in athletes: a randomized, double-blinded, placebo-controlled trial. Antioxidants. 2020;9(9):823. doi: 10.3390/antiox9090823 32899227 PMC7555239

[252] Kizaki K, Terada T, Arikawa H, et al. Effect of reduced coenzyme Q10 (ubiquinol) supplementation on blood pressure and muscle damage during kendo training camp: a double-blind, randomized controlled study. J Sports Med Phys Fitness. 2014;55(7-8):797–804. 25369277

[253] Kon M, Tanabe K, Akimoto T, et al. Reducing exercise-induced muscular injury in kendo athletes with supplementation of coenzyme Q10. Br J Nutr. 2008;100(4):903–909. doi: 10.1017/S0007114508926544 18284711

[254] Suzuki Y, Nagato S, Sakuraba K, et al. Short-term ubiquinol-10 supplementation alleviates tissue damage in muscle and fatigue caused by strenuous exercise in Male distance runners. Int J Vitam Nutr Res. 2020 10.1024/0300-9831/a000627 32003645

[255] Weston SB, Zhou S, Weatherby RP, et al. Does exogenous coenzyme Q10 affect aerobic capacity in endurance athletes? Int J Sport Nutr. 1997;7(3):197–206. doi: 10.1123/ijsn.7.3.197 9286743

[256] Sarmiento A, Diaz‐Castro J, Pulido‐Moran M, et al. Short‐term ubiquinol supplementation reduces oxidative stress associated with strenuous exercise in healthy adults: a randomized trial. BioFactors. 2016;42(6):612–622. doi: 10.1002/biof.1297 27193497

[257] Deichmann RE, Lavie CJ, Dornelles AC. Impact of coenzyme Q-10 on parameters of cardiorespiratory fitness and muscle performance in older athletes taking statins. Phys Sportsmed. 2012;40(4):88–95. doi: 10.3810/psm.2012.11.1991 23306418

[258] Emami A. The impact of pre-cooling and CoQ10 supplementation on mediators of inflammatory cytokines in elite swimmers. Nutr Cancer. 2020;72(1):41–51. doi: 10.1080/01635581.2019.1614200 31094229

[259] Emami A, Bazargani-Gilani B. Effect of oral CoQ 10 supplementation along with precooling strategy on cellular response to oxidative stress in elite swimmers. Food Function. 2018;9(8):4384–4393. doi: 10.1039/C8FO00960K 30058646

[260] Emami A, Tofighi A, Asri-Rezaei S, et al. The effect of short-term coenzyme Q10 supplementation and pre-cooling strategy on cardiac damage markers in elite swimmers. Br J Nutr. 2018;119(4):381–390. doi: 10.1017/S0007114517003774 29498347

[261] Holloway CJ, Murray AJ, Mitchell K, et al. Oral coenzyme Q10 supplementation does not prevent cardiac alterations during a high altitude trek to everest base cAMP. High Altitude Med Biol. 2014;15(4):459–467. 10.1089/ham.2013.1053 PMC4273181 24661196

[262] Malm C, Svensson M, Ekblom B, et al. Effects of ubiquinone‐10 supplementation and high intensity training on physical performance in humans. Acta Physiol Scand. 1997;161(3):379–384. doi: 10.1046/j.1365-201X.1997.00198.x 9401591

[263] Mohammadi M, Naderi A, Siavoshi H, et al. The effect of short-term use of coenzyme Q10 supplementation on selected physical and physiological characteristics of young Male elite wrestlers. Rev Brasileira de Nutrição Esportiva. 2020;14(84):53–65.

[264] Orlando P, Silvestri S, Galeazzi R, et al. Effect of ubiquinol supplementation on biochemical and oxidative stress indexes after intense exercise in young athletes. Redox Rep. 2018;23(1):136–145. doi: 10.1080/13510002.2018.1472924 29734881 PMC6748686

[265] Östman B, Sjödin A, Michaëlsson K, et al. Coenzyme Q10 supplementation and exercise-induced oxidative stress in humans. Nutrition. 2012;28(4):403–417. 22079391 10.1016/j.nut.2011.07.010

[266] Shimizu K, Kon M, Tanimura Y, et al. Coenzyme Q10 supplementation downregulates the increase of monocytes expressing toll-like receptor 4 in response to 6-day intensive training in kendo athletes. Appl Physiol Nutr Metabol. 2015;40(6):575–581. doi: 10.1139/apnm-2014-0556 25941765

[267] Ylikoski T, Piirainen J, Hanninen O, et al. The effect of coenzyme Q10 on the exercise performance of cross-country skiers. Mol Aspects Med. 1997;18:283–290. doi: 10.1016/S0098-2997(97)00038-1 9266538

[268] Svensson M, Malm C, Tonkonogi M, et al. Effect of Q10 supplementation on tissue Q10 levels and adenine nucleotide catabolism during high-intensity exercise. Int J Sport Nutr Exerc Metab. 1999;9(2):166–180. doi: 10.1123/ijsn.9.2.166 10362453

[269] Díaz-Castro J, Guisado R, Kajarabille N, et al. Coenzyme Q 10 supplementation ameliorates inflammatory signaling and oxidative stress associated with strenuous exercise. Eur J Nutr. 2012;51:791–799. doi: 10.1007/s00394-011-0257-5 21990004

[270] Drobnic F, Lizarraga MA, Caballero-García A, et al. Coenzyme Q(10) supplementation and its impact on exercise and sport performance in humans: a recovery or a performance-enhancing molecule? Nutrients. 2022 Apr 26;14(9):1811. doi: 10.3390/nu14091811 35565783 PMC9104583

[271] Fernandes MSS, Fidelis D, Aidar FJ, et al. Coenzyme Q10 supplementation in athletes: a systematic review. Nutrients. 2023 Sep 15;15(18):3990. doi: 10.3390/nu15183990 37764774 PMC10535924

[272] Talebi S, Pourgharib Shahi MH, Zeraattalab-Motlagh S, et al. The effects of coenzyme Q10 supplementation on biomarkers of exercise-induced muscle damage, physical performance, and oxidative stress: a GRADE-assessed systematic review and dose-response meta-analysis of randomized controlled trials. Clin Nutr ESPEN. 2024;60:122–134. 2024/04/01/. doi: 10.1016/j.clnesp.2024.01.015 38479900

[273] Karlapudi V, Prasad Mungara AVV, Sengupta K, et al. A placebo-controlled double-blind study demonstrates the clinical efficacy of a novel herbal formulation for relieving joint discomfort in human subjects with osteoarthritis of knee. J Med Food. 2018;21(5):511–520. doi: 10.1089/jmf.2017.0065 29708818

[274] Sun J, Chen F, Braun C, et al. Role of curcumin in the management of pathological pain. Phytomedicine. 2018;48:129–140. doi: 10.1016/j.phymed.2018.04.045 30195871

[275] Mollazadeh H, Cicero AF, Blesso CN, et al. Immune modulation by curcumin: the role of interleukin-10. Crit Rev Food Sci Nutr. 2019;59(1):89–101. doi: 10.1080/10408398.2017.1358139 28799796

[276] Li H, Sureda A, Devkota HP, et al. Curcumin, the golden spice in treating cardiovascular diseases. Biotech Adv. 2020;38:107343. doi: 10.1016/j.biotechadv.2019.01.010 30716389

[277] Patel SS, Acharya A, Ray R, et al. Cellular and molecular mechanisms of curcumin in prevention and treatment of disease. Crit Rev Food Sci Nutr. 2020;60(6):887–939. doi: 10.1080/10408398.2018.1552244 30632782

[278] Salehi B, Stojanović-Radić Z, Matejić J, et al. The therapeutic potential of curcumin: a review of clinical trials. Eur J Med Chem. 2019;163:527–545. doi: 10.1016/j.ejmech.2018.12.016 30553144

[279] Chilelli NC, Ragazzi E, Valentini R, et al. Curcumin and boswellia serrata modulate the glyco-oxidative status and lipo-oxidation in master athletes. Nutrients. 2016;8(11):745. doi: 10.3390/nu8110745 27879642 PMC5133128

[280] Delecroix B, Leduc C, Dawson B, et al. Curcumin and piperine supplementation and recovery following exercise induced muscle damage: a randomized controlled trial. J Sports Sci Med. 2017;16(1):147. 28344463 PMC5358025

[281] Falgiano PA, Gillum TL, Schall ZJ, et al. Dietary curcumin supplementation does not alter peripheral blood mononuclear cell responses to exertional heat stress. Eur J Appl Physiol. 2018;118:2707–2717. doi: 10.1007/s00421-018-3998-5 30276476

[282] McAllister MJ, Basham SA, Waldman HS, et al. Effects of curcumin on the oxidative stress response to a dual stress challenge in trained men. J Dietary Suppl. 2020;17(3):261–272. doi: 10.1080/19390211.2018.1515142 30580652

[283] McFarlin BK, Venable AS, Henning AL, et al. Reduced inflammatory and muscle damage biomarkers following oral supplementation with bioavailable curcumin. BBA Clin. 2016;5:72–78. doi: 10.1016/j.bbacli.2016.02.003 27051592 PMC4802396

[284] Nicol LM, Rowlands DS, Fazakerly R, et al. Curcumin supplementation likely attenuates delayed onset muscle soreness (DOMS). Eur J Appl Physiol. 2015 Aug;115(8):1769–1777. doi: 10.1007/s00421-015-3152-6 25795285

[285] Sciberras JN, Galloway SDR, Fenech A, et al. The effect of turmeric (Curcumin) supplementation on cytokine and inflammatory marker responses following 2 hours of endurance cycling. J Int Soc Sports Nutr. 2015;12(1):5. 2015/01/21. doi: 10.1186/s12970-014-0066-3 25628521 PMC4307740

[286] Szymanski MC, Gillum TL, Gould LM, et al. Short-term dietary curcumin supplementation reduces gastrointestinal barrier damage and physiological strain responses during exertional heat stress. J Appl Physiol. 2018;124(2):330–340. doi: 10.1152/japplphysiol.00515.2017 28935827

[287] Takahashi M, Suzuki K, Kim HK, et al. Effects of curcumin supplementation on exercise-induced oxidative stress in humans. Int J Sports Med. 2014 Jun;35(6):469–475. 24165958 10.1055/s-0033-1357185

[288] Tanabe Y, Chino K, Ohnishi T, et al. Effects of oral curcumin ingested before or after eccentric exercise on markers of muscle damage and inflammation. Scand J Med Sci Sports. 2019;29(4):524–534. doi: 10.1111/sms.13373 30566760

[289] Tanabe Y, Chino K, Sagayama H, et al. Effective timing of curcumin ingestion to attenuate eccentric exercise-induced muscle soreness in men. J Nutr Sci Vitaminol (Tokyo). 2019;65(1):82–89. doi: 10.3177/jnsv.65.82 30814417

[290] Suhett LG, de Miranda Monteiro Santos R, Silveira BKS, et al. Effects of curcumin supplementation on sport and physical exercise: a systematic review. Crit Rev Food Sci Nutr. 2021;61(6):946–958. doi: 10.1080/10408398.2020.1749025 32282223

[291] Chilelli NC, Ragazzi E, Valentini R, et al. Curcumin and boswellia serrata modulate the glyco-oxidative status and lipo-oxidation in master athletes. Nutrients. 2016 Nov 21;8(11):745. doi: 10.3390/nu8110745 27879642 PMC5133128

[292] El-Saadony MT, Yang T, Korma SA, et al. Impacts of turmeric and its principal bioactive curcumin on human health: pharmaceutical, medicinal, and food applications: a comprehensive review. Front Nutr. 2022;9:1040259. doi: 10.3389/fnut.2022.1040259 36712505 PMC9881416

[293] Kreider RB, Gonzalez DE, Hines K, et al. Safety of creatine supplementation: analysis of the prevalence of reported side effects in clinical trials and adverse event reports. J Int Soc Sports Nutr. 2025;22(sup1):2488937. 2025/09/30. doi: 10.1080/15502783.2025.2488937 40198156 PMC11983583

[294] Kreider RB, Kalman DS, Antonio J, et al. International Society of Sports Nutrition position stand: safety and efficacy of creatine supplementation in exercise, sport, and Medicine. J Int Soc Sports Nutr. 2017;14:18. doi: 10.1186/s12970-017-0173-z 28615996 PMC5469049

[295] Butts J, Jacobs B, Silvis M. Creatine use in sports. Sports Health. 2018 Jan/Feb;10(1):31–34. doi: 10.1177/1941738117737248 29059531 PMC5753968

[296] Meyer LE, Machado LB, Santiago APS, et al. Mitochondrial creatine kinase activity prevents reactive oxygen species generation: antioxidant role of mitochondrial kinase-dependent ADP re-cycling activity. J Biol Chem. 2006;281(49):37361–37371. doi: 10.1074/jbc.M604123200 17028195

[297] Amiri E, Sheikholeslami-Vatani D. The role of resistance training and creatine supplementation on oxidative stress, antioxidant defense, muscle strength, and quality of life in older adults [Original Research]. Front Public Health. 2023;11. 2023–May–02. doi: 10.3389/fpubh.2023.1062832 PMC10189876 37206869

[298] Arazi H, Eghbali E, Suzuki K. Creatine supplementation, physical exercise and oxidative stress markers: a review of the mechanisms and effectiveness. Nutrients. 2021 Mar 6;13(3):869. doi: 10.3390/nu13030869 33800880 PMC8000194

[299] Bassit R, Curi R, Costa Rosa LFBP. Creatine supplementation reduces plasma levels of pro-inflammatory cytokines and PGE 2 after a half-ironman competition. Amino Acids. 2008;35:425–431. doi: 10.1007/s00726-007-0582-4 17917696

[300] Clarke H, Kim DH, Meza CA, et al. The evolving applications of creatine supplementation: could creatine improve vascular health? Nutrients. 2020 Sep 16;12(9):2834. doi: 10.3390/nu12092834 32947909 PMC7551337

[301] Cordingley DM, Cornish SM, Candow DG. Anti-inflammatory and anti-catabolic effects of creatine supplementation: a brief review. Nutrients. 2022;14(3):544. doi: 10.3390/nu14030544 35276903 PMC8839648

[302] Deminice R, Jordao AA. Creatine supplementation decreases plasma lipid peroxidation markers and enhances anaerobic performance in rats. Redox Rep. 2016 Jan;21(1):31–36. doi: 10.1179/1351000215Y.0000000020 26083240 PMC6837342

[303] Deminice R, Rosa FT, Franco GS, et al. Effects of creatine supplementation on oxidative stress and inflammatory markers after repeated-sprint exercise in humans. Nutrition. 2013 Sep;29(9):1127–1132. doi: 10.1016/j.nut.2013.03.003 23800565

[304] Rahimi R. Creatine supplementation decreases oxidative DNA damage and lipid peroxidation induced by a single bout of resistance exercise. J Strength Condition Res. 2011;25(12):3448–3455. doi: 10.1519/JSC.0b013e3182162f2b 22080314

[305] Sestili P, Martinelli C, Bravi G, et al. Creatine supplementation affords cytoprotection in oxidatively injured cultured mammalian cells via direct antioxidant activity. Free Radic Biol Med. 2006 Mar 1;40(5):837–849. doi: 10.1016/j.freeradbiomed.2005.10.035 16520236

[306] Matthews RT, Yang L, Jenkins BG, et al. Neuroprotective effects of creatine and cyclocreatine in animal models of Huntington's disease. J Neurosci. 1998 Jan 1;18(1):156–163. doi: 10.1523/JNEUROSCI.18-01-00156.1998 9412496 PMC6793381

[307] Lawler JM, Barnes WS, Wu G, et al. Direct antioxidant properties of creatine. Biochem Biophys Res Commun. 2002 Jan 11;290(1):47–52. doi: 10.1006/bbrc.2001.6164 11779131

[308] Kingsley M, Cunningham D, Mason L, et al. Role of creatine supplementation on exercise-induced cardiovascular function and oxidative stress. Oxid Med Cell Longev. 2009 Sep–Oct;2(4):247–254. doi: 10.4161/oxim.2.4.9415 20716911 PMC2763263

[309] Percário S, Domingues SP, Teixeira LF, et al. Effects of creatine supplementation on oxidative stress profile of athletes. J Int Soc Sports Nutr. 2012 Dec 21;9(1):56. doi: 10.1186/1550-2783-9-56 23259853 PMC3543170

[310] Santos RV, Bassit RA, Caperuto EC, et al. The effect of creatine supplementation upon inflammatory and muscle soreness markers after a 30km race. Life Sci. 2004 Sep 3;75(16):1917–1924. doi: 10.1016/j.lfs.2003.11.036 15306159

[311] Aguiar AF, Januário RS, Junior RP, et al. Long-term creatine supplementation improves muscular performance during resistance training in older women. Eur J Appl Physiol. 2013 Apr;113(4):987–996. doi: 10.1007/s00421-012-2514-6 23053133

[312] Kreider RB, Stout JR. Creatine in health and disease. Nutrients. 2021 Jan 29;13(2):447. doi: 10.3390/nu13020447 33572884 PMC7910963

[313] Lawler JM, Barnes WS, Wu G, et al. Direct antioxidant properties of creatine. Biochem Biophys Res Commun. 2002;290(1):47–52. doi: 10.1006/bbrc.2001.6164 11779131

[314] Rawson ES, Conti MP, Miles MP. Creatine supplementation does not reduce muscle damage or enhance recovery from resistance exercise. J Strength Cond Res. 2007 Nov;21(4):1208–1213. doi: 10.1519/00124278-200711000-00039 18076246

[315] Stiefvatter L, Neumann U, Rings A, et al. The microalgae phaeodactylum tricornutum is well suited as a food with positive effects on the intestinal microbiota and the generation of SCFA: results from a pre-clinical study. Nutrients. 2022 Jun 16;14(12):2504. doi: 10.3390/nu14122504 35745233 PMC9229211

[316] Peng J, Yuan JP, Wu CF, et al. Fucoxanthin, a marine carotenoid present in brown seaweeds and Diatoms: metabolism and bioactivities relevant to human health. Mar Drugs. 2011;9(10):1806–1828. doi: 10.3390/md9101806 22072997 PMC3210606

[317] Yan X, Chuda Y, Suzuki M, et al. Fucoxanthin as the major antioxidant in hijikia fusiformis, a common edible seaweed. Biosci Biotechnol Biochem. 1999;63(3):605–607. doi: 10.1271/bbb.63.605 10227153

[318] Hosokawa M, Okada T, Mikami N, et al. Bio-functions of marine carotenoids. Food Sci Biotechnol. 2009;18(1):1–11.

[319] Sangeetha RK, Bhaskar N, Baskaran V. Comparative effects of β-carotene and fucoxanthin on retinol deficiency induced oxidative stress in rats. Mol Cell Biochem. 2009;331(1):59–67. doi: 10.1007/s11010-009-0145-y. 2009/11/01. 19421712

[320] Abidov M, Ramazanov Z, Seifulla R, et al. The effects of Xanthigen™ in the weight management of obese premenopausal women with non-alcoholic fatty liver disease and normal liver fat. Diabetes Obes Metab. 2010;12(1):72–81. doi: 10.1111/j.1463-1326.2009.01132.x 19840063

[321] Dickerson B, Maury J, Jenkins V, et al. Effects of supplementation with microalgae extract from phaeodactylum tricornutum (Mi136) to support benefits from a weight management intervention in overweight women. Nutrients. 2024;16(7):990. doi: 10.3390/nu16070990 38613023 PMC11013338

[322] Leonard M, Maury J, Dickerson B, et al. Effects of dietary supplementation of a microalgae extract containing fucoxanthin combined with guarana on cognitive function and gaming performance. Nutrients. 2023 Apr 15;15(8):1918. doi: 10.3390/nu15081918 37111136 PMC10142384

[323] Yoo C, Maury J, Gonzalez DE, et al. Effects of supplementation with a microalgae extract from phaeodactylum tricornutum containing fucoxanthin on cognition and markers of health in older individuals with perceptions of cognitive decline. Nutrients. 2024 Sep 5;16(17):2999. doi: 10.3390/nu16172999 39275314 PMC11397347

[324] Stiefvatter L, Frick K, Lehnert K, et al. Potentially beneficial effects on healthy aging by supplementation of the EPA-Rich microalgae phaeodactylum tricornutum or its Supernatant-A randomized controlled pilot trial in elderly individuals. Mar Drugs. 2022 Nov 15;20(11):716. doi: 10.3390/md20110716 36421994 PMC9694444

[325] Dickerson B, Maury J, Jenkins V, et al. Effects of supplementation with microalgae extract from phaeodactylum tricornutum (Mi136) to support benefits from a weight management intervention in overweight women. Nutrients. 2024 Mar 28;16(7):990. doi: 10.3390/nu16070990 38613023 PMC11013338

[326] McFadden BA, Vincenty CS, Chandler AJ, et al. Effects of fucoidan supplementation on inflammatory and immune response after high-intensity exercise. J Int Soc Sports Nutr. 2023;20(1):2224751. 2023/12/31. doi: 10.1080/15502783.2023.2224751 37331983 PMC10281294

[327] Minich DM, Brown BI. A review of dietary (Phyto)Nutrients for glutathione support. Nutrients. 2019 Sep 3;11(9):2073. doi: 10.3390/nu11092073 31484368 PMC6770193

[328] Saitoh T, Satoh H, Nobuhara M, et al. Intravenous glutathione prevents renal oxidative stress after coronary angiography more effectively than oral N-acetylcysteine. Heart Vessels. 2011;26:465–472. doi: 10.1007/s00380-010-0078-0 21127883

[329] Schmitt B, Vicenzi M, Garrel C, et al. Effects of N-acetylcysteine, oral glutathione (GSH) and a novel sublingual form of GSH on oxidative stress markers: a comparative crossover study. Redox Biol. 2015;6:198–205. doi: 10.1016/j.redox.2015.07.012 26262996 PMC4536296

[330] Allen J, Bradley RD. Effects of oral glutathione supplementation on systemic oxidative stress biomarkers in human volunteers. J Altern Complement Med. 2011 Sep;17(9):827–833. doi: 10.1089/acm.2010.0716 21875351 PMC3162377

[331] Richie JP, Nichenametla S, Neidig W, et al. Randomized controlled trial of oral glutathione supplementation on body stores of glutathione. Eur J Nutr. 2015;54(2):251–263. 2015/03/01. doi: 10.1007/s00394-014-0706-z 24791752

[332] Hwang P, Morales Marroquín FE, Gann J, et al. Eight weeks of resistance training in conjunction with glutathione and L-Citrulline supplementation increases lean mass and has no adverse effects on blood clinical safety markers in resistance-trained males. J Int Soc Sports Nutr. 2018;15(1):30. 2018/06/27. doi: 10.1186/s12970-018-0235-x 29945625 PMC6020314

[333] Aoi W, Ogaya Y, Takami M, et al. Glutathione supplementation suppresses muscle fatigue induced by prolonged exercise via improved aerobic metabolism. J Int Soc Sports Nutr. 2015;12:7. doi: 10.1186/s12970-015-0067-x 25685110 PMC4328900

[334] Atkuri KR, Mantovani JJ, Herzenberg LA, et al. N-Acetylcysteine--a safe antidote for cysteine/glutathione deficiency. Curr Opin Pharmacol. 2007 Aug;7(4):355–359. doi: 10.1016/j.coph.2007.04.005 17602868 PMC4540061

[335] Paschalis V, Theodorou AA, Margaritelis NV, et al. N-acetylcysteine supplementation increases exercise performance and reduces oxidative stress only in individuals with low levels of glutathione. Free Radic Biol Med. 2018 Feb 1;115:288–297. doi: 10.1016/j.freeradbiomed.2017.12.007 29233792

[336] Rhodes K, Braakhuis A. Performance and side effects of supplementation with N-acetylcysteine: a systematic review and meta-analysis. Sports Med. 2017;47:1619–1636. doi: 10.1007/s40279-017-0677-3 28102488

[337] Murase T, Haramizu S, Shimotoyodome A, et al. Green tea extract improves endurance capacity and increases muscle lipid oxidation in mice. Am J Physiol-Regulatory Integrat Comparat Physiol. 2005;288:R708–R715. doi: 10.1152/ajpregu.00693.2004 15563575

[338] Nobari H, Saedmocheshi S, Chung LH, et al. An overview on how exercise with Green tea consumption can prevent the production of reactive oxygen species and improve sports performance. Int J Environ Res Public Health. 2021 Dec 25;19(1):218. doi: 10.3390/ijerph19010218 35010479 PMC8750450

[339] Prasanth MI, Sivamaruthi BS, Chaiyasut C, et al. A review of the role of Green tea (*Camellia sinensis*) in antiphotoaging, stress resistance, neuroprotection, and autophagy. Nutrients. 2019;11(2):474. doi: 10.3390/nu11020474 30813433 PMC6412948

[340] Rojano D, Naranjo Orellana J, Berral Aguilar A, et al. Regular green tea supplementation increases total antioxidant status and reduces exercise-induced oxidative stress: a systematic review. 2022. 10.1016/j.nutres.2021.08.004 34624703

[341] Saedmocheshi S, Saghebjoo M, Vahabzadeh Z, et al. Aerobic training and Green tea extract protect against N-methyl-N-nitrosourea-induced prostate cancer. Med Sci Sports Exerc. 2019;51(11):2210–2216. doi: 10.1249/MSS.0000000000002054 31626054

[342] Venables MC, Hulston CJ, Cox HR, et al. Green tea extract ingestion, fat oxidation, and glucose tolerance in healthy humans. AJCN. 2008 Mar;87(3):778–784. doi: 10.1093/ajcn/87.3.778 18326618

[343] Younes M, Aggett P, Aguilar F. Scientific opinion on the safety of Green tea catechins. EFSA J. 2018;16(4):e05239. doi: 10.2903/j.efsa.2018.5239 32625874 PMC7009618

[344] Hernández-Lepe MA, Hernández-Ontiveros DA, Chávez-Guevara IA, et al. Impact of exercise training at maximal fat oxidation intensity on metabolic and epigenetic parameters in patients with overweight and obesity: study protocol of a randomized controlled trial. J Funct Morphol Kinesiol. 2024 Oct 31;9(4):214. doi: 10.3390/jfmk9040214 39584867 PMC11587150

[345] Younes M, Aggett P, Aguilar F, et al. Scientific opinion on the safety of Green tea catechins. EFSA J. 2018;16. doi: 10.2903/j.efsa.2018.5239 PMC7009618 32625874

[346] US Department of Agriculture ARS. 2015. USDA national nutrient database for standard reference.

[347] Ribaya-Mercado JD, Blumberg JB. Lutein and zeaxanthin and their potential roles in disease prevention. J Am Coll Nutr. 2004;23(sup6):567S–587S. 2004/12/01. 15640510 10.1080/07315724.2004.10719427

[348] Scripsema NK, Hu DN, Rosen RB. Lutein, zeaxanthin, and meso-zeaxanthin in the clinical management of eye disease. J Ophthalmol. 2015;2015:865179. doi: 10.1155/2015/865179 26819755 PMC4706936

[349] Jia YP, Sun L, Yu HS, et al. The pharmacological effects of lutein and zeaxanthin on visual disorders and cognition diseases. Molecules. 2017 Apr 20;22(4):610. doi: 10.3390/molecules22040610 28425969 PMC6154331

[350] Ma L, Dou H-L, Wu Y-Q, et al. Lutein and zeaxanthin intake and the risk of age-related macular degeneration: a systematic review and meta-analysis. Br J Nutr. 2012;107(3):350–359. doi: 10.1017/S0007114511004260 21899805

[351] Wilson LM, Tharmarajah S, Jia Y, et al. The effect of Lutein/Zeaxanthin intake on human macular pigment optical density: a systematic review and meta-analysis. Adv Nutr. 2021;12(6):2244–2254. 2021/11/01/. doi: 10.1093/advances/nmab071 34157098 PMC8634499

[352] Liu X-H, Yu R-B, Liu R, et al. Association between lutein and zeaxanthin status and the risk of cataract: a meta-analysis. Nutrients. 2014;6(1):452–465. doi: 10.3390/nu6010452 24451312 PMC3916871

[353] Raman G, Haslam D, Avendano E, et al. Lutein/zeaxanthin intake and visual outcomes in adults with healthy eyes: qualitative gap analysis. Cogent Medicine. 2019;6(1):1683939. doi: 10.1080/2331205X.2019.1683939

[354] Tan JS, Wang JJ, Flood V, et al. Dietary antioxidants and the long-term incidence of age-related macular degeneration: the blue mountains eye study. Ophthalmology. 2008;115(2):334–341. doi: 10.1016/j.ophtha.2007.03.083 17664009

[355] Moeller SM, Parekh N, Tinker L, et al. Associations between intermediate age-related macular degeneration and lutein and zeaxanthin in the carotenoids in age-related eye disease study (CAREDS): ancillary study of the Women's health initiative. Arch Ophthal. 2006;124(8):1151–1162. doi: 10.1001/archopht.124.8.1151 16908818

[356] Cho E, Hankinson SE, Rosner B, et al. Prospective study of lutein/zeaxanthin intake and risk of age-related macular degeneration. Am J Clin Nutr. 2008;87(6):1837–1843. doi: 10.1093/ajcn/87.6.1837 18541575 PMC2504741

[357] Bovier ER, Hammond BR. A randomized placebo-controlled study on the effects of lutein and zeaxanthin on visual processing speed in young healthy subjects. Arch Biochem Biophys. 2015;572:54–57. doi: 10.1016/j.abb.2014.11.012 25483230

[358] Bovier ER, Renzi LM, Hammond BR. A double-blind, placebo-controlled study on the effects of lutein and zeaxanthin on neural processing speed and efficiency. PLoS One. 2014;9(9):e108178. doi: 10.1371/journal.pone.0108178 25251377 PMC4176961

[359] Landrum J, Bone R, Mendez V, et al. Comparison of dietary supplementation with lutein diacetate and lutein: a pilot study of the effects on serum and macular pigment. Acta Biochim Pol. 2012;59(1):167–169. doi: 10.18388/abp.2012_2198 22428144

[360] Thurnham DI, Nolan JM, Howard AN, et al. Macular response to supplementation with differing xanthophyll formulations in subjects with and without age-related macular degeneration. Graefe's Arch Clin Exp Ophthalmol. 2015;253:1231–1243. doi: 10.1007/s00417-014-2811-3 25311651

[361] Yao Y, Qiu Q-h, Wu X-W, et al. Lutein supplementation improves visual performance in Chinese drivers: 1-year randomized, double-blind, placebo-controlled study. Nutrition. 2013;29(7-8):958–964. doi: 10.1016/j.nut.2012.10.017 23360692

[362] Hammond BR, Fletcher LM. Influence of the dietary carotenoids lutein and zeaxanthin on visual performance: application to baseball123. Am J Clin Nutr. 2012;96(5):1207S–1213S. 2012/11/01/. doi: 10.3945/ajcn.112.034876 23053558

[363] Yoshida K, Sakai O, Honda T, et al. Effects of astaxanthin, lutein, and zeaxanthin on Eye–Hand coordination and smooth-pursuit eye movement after visual display terminal operation in healthy subjects: a randomized, double-blind placebo-controlled intergroup trial. Nutrients. 2023;15(6):1459. doi: 10.3390/nu15061459 36986186 PMC10054128

[364] Murphy CH, Duggan E, Davis J, et al. Plasma lutein and zeaxanthin concentrations associated with musculoskeletal health and incident frailty in the Irish longitudinal study on ageing (TILDA). Exp Gerontol. 2023;171:112013. 2023/01/01/. doi: 10.1016/j.exger.2022.112013 36336250

[365] Sahni S, Dufour AB, Fielding RA, et al. Total carotenoid intake is associated with reduced loss of grip strength and gait speed over time in adults: the framingham offspring study. Am J Clin Nutr. 2021;113(2):437–445. doi: 10.1093/ajcn/nqaa288 33181830 PMC7851823

[366] Mason RP, Libby P, Bhatt DL. Emerging mechanisms of cardiovascular protection for the Omega-3 fatty acid eicosapentaenoic acid. Arter Thromb Vasc Biol. 2020;40(5):1135–1147. doi: 10.1161/ATVBAHA.119.313286 PMC7176343 32212849

[367] Sherratt SC, Mason RP. Eicosapentaenoic acid and docosahexaenoic acid have distinct membrane locations and lipid interactions as determined by X-ray diffraction. Chem Phys Lipids. 2018;212:73–79. doi: 10.1016/j.chemphyslip.2018.01.002 29355517

[368] Mason RP, Jacob RF, Shrivastava S, et al. Eicosapentaenoic acid reduces membrane fluidity, inhibits cholesterol domain formation, and normalizes bilayer width in atherosclerotic-like model membranes. Biochim Biophys Acta Biomembr. 2016;1858(12):3131–3140. doi: 10.1016/j.bbamem.2016.10.002 27718370

[369] Mason RP, Sherratt SC, Jacob RF. Eicosapentaenoic acid inhibits oxidation of ApoB-containing lipoprotein particles of different size in vitro when administered alone or in combination with atorvastatin active metabolite compared with other triglyceride-lowering agents. J Cardiovasc Pharmacol. 2016;68(1):33–40. doi: 10.1097/FJC.0000000000000379 26945158 PMC4936437

[370] Tatsumi YA-O, Kato A, & Sango K, et al. (2019). Omega-3 polyunsaturated fatty acids exert anti-oxidant effects through the nuclear factor (erythroid-derived 2)-related factor 2 pathway in immortalized mouse Schwann cells. (2040-1124 (Electronic)). 10.1111/jdi.12931 PMC6497605 30216708

[371] Zgórzyńska E, Dziedzic B, & Gorzkiewicz A, et al. (2017). Omega-3 polyunsaturated fatty acids improve the antioxidative defense in rat astrocytes via an Nrf2-dependent mechanism. (2299-5684 (Electronic)). 10.1016/j.pharep.2017.04.009 28662394

[372] Stanley WC, Khairallah RJ, Dabkowski ER. Update on lipids and mitochondrial function: impact of dietary n-3 polyunsaturated fatty acids. Curr Opin Clin Nutr Metabolic Care. 2012;15(2):122–126. 10.1097/MCO.0b013e32834fdaf7 PMC4067133 22248591

[373] Pepe S, Tsuchiya NF, & Lakatta EG, et al. (1999). PUFA and aging modulate cardiac mitochondrial membrane lipid composition and Ca2+ activation of PDH. (0002-9513 (Print)). 10.1152/ajpheart.1999.276.1.H149 9887028

[374] Oppedisano FA-O, Macrì R, & Gliozzi M, et al. (2020). The Anti-Inflammatory and Antioxidant Properties of n-3 PUFAs: Their Role in Cardiovascular Protection. LID - doi: 10.3390/biomedicines8090306 [doi] LID - 306. (2227-9059 (Print)). PMC7554783 32854210

[375] Erdogan H, Fadillioglu E, Ozgocmen S, et al. Effect of fish oil supplementation on plasma oxidant/antioxidant status in rats. Prostaglandins Leukot Essent Fatty Acids. 2004 Sep;71(3):149–152. doi: 10.1016/j.plefa.2004.02.001 15253883

[376] Heshmati J, Morvaridzadeh M, Maroufizadeh S, et al. Omega-3 fatty acids supplementation and oxidative stress parameters: a systematic review and meta-analysis of clinical trials. Pharmacol Res. 2019;149:104462. 2019/11/01/. doi: 10.1016/j.phrs.2019.104462 31563611

[377] Moosavian SP, Arab A, Mehrabani S, et al. The effect of omega-3 and vitamin E on oxidative stress and inflammation: systematic review and meta-analysis of randomized controlled trials. Hogrefe AG. 2020;90(5–6):553–563. doi: 10.1024/0300-9831/a000599 31442100

[378] Ghiasvand R, Djalali MF, & Djazayery S, et al. (2010) Effect of eicosapentaenoic Acid (EPA) and vitamin e on the blood levels of inflammatory markers, antioxidant enzymes, and lipid peroxidation in Iranian basketball players. (2251-6085 (Print)). PMC3468972 23112985

[379] Bozcali E, Babalik E, Himmetoglu S, et al. ω-3 fatty acid treatment in cardiac syndrome X: a double-blind, randomized, placebo-controlled clinical study. Coron Artery Dis. 2013;24(4):328–333. doi: 10.1097/MCA.0b013e32835f3005 23425772

[380] Eftekhari MH, Aliasghari F, Babaei-Beigi MA, et al. Effect of conjugated linoleic acid and omega-3 fatty acid supplementation on inflammatory and oxidative stress markers in atherosclerotic patients. ARYA Atheroscler. 2013;9(6):311. 24575132 PMC3933057

[381] Nemati A, Mahdavi R, Faizi-Khankandi I, et al. Effects of omega-3 fatty acids supplementation on oxidative stress and MMP2/9 in patients with gastric cancer during chemotherapy. 2012;1858–1863.

[382] Shidfar F, Keshavarz A, Hosseyni S, et al. Effects of omega-3 fatty acid supplements on serum lipids, apolipoproteins and malondialdehyde in type 2 diabetes patients. East Mediterr Health J. 2008;14(2). 18561722

[383] Mohammadi E, Rafraf M. Benefits of omega-3 fatty acids supplementation on serum paraoxonase 1 activity and lipids ratios in polycystic ovary syndrome. Health Promot Perspect. 2012;2(2):197. 24688934 10.5681/hpp.2012.023 PMC3963626

[384] Amini M, Bahmani F, Foroozanfard F, et al. Retracted article: the effects of fish oil omega-3 fatty acid supplementation on mental health parameters and metabolic status of patients with polycystic ovary syndrome: a randomized, double-blind, placebo-controlled trial. J Psychosomat Obstetr Gynecol. 2024;45(1):1508282. doi: 10.1080/0167482X.2018.1508282 30230402

[385] Asemi Z, Soleimani A, Bahmani F, et al. Effect of the omega-3 fatty acid plus vitamin E supplementation on subjective global assessment score, glucose metabolism, and lipid concentrations in chronic hemodialysis patients. Mol Nutr Food Res. 2016;60(2):390–398. doi: 10.1002/mnfr.201500584 26518514

[386] Rahmani E, Samimi M, Ebrahimi FA, et al. The effects of omega-3 fatty acids and vitamin E co-supplementation on gene expression of lipoprotein (a) and oxidized low-density lipoprotein, lipid profiles and biomarkers of oxidative stress in patients with polycystic ovary syndrome. Mol Cell Endocrinol. 2017;439:247–255. doi: 10.1016/j.mce.2016.09.008 27619403

[387] Saboori S, Koohdani F, Nematipour E, et al. Beneficial effects of omega-3 and vitamin E coadministration on gene expression of SIRT1 and PGC1α and serum antioxidant enzymes in patients with coronary artery disease. Nutr Metabol Cardiovascular Dis. 2016;26(6):489–494. doi: 10.1016/j.numecd.2015.11.013 27033026

[388] Taghizadeh M, Tamtaji OR, Dadgostar E, et al. The effects of omega-3 fatty acids and vitamin E co-supplementation on clinical and metabolic status in patients with Parkinson's disease: a randomized, double-blind, placebo-controlled trial. Neurochem Int. 2017;108:183–189. doi: 10.1016/j.neuint.2017.03.014 28342967

[389] Barquilha G, Dos Santos CMM, Caçula KG, et al. Fish oil supplementation improves the repeated-bout effect and redox balance in 20–30-year-old men submitted to strength training. Nutrients. 2023;15(7):1708. doi: 10.3390/nu15071708 37049548 PMC10096819

[390] Lee S-R, Directo D, Khamoui AV. Fish oil administration combined with resistance exercise training improves strength, resting metabolic rate, and inflammation in older adults. Aging Clin Exp Res. 2022;34(12):3073–3081. doi: 10.1007/s40520-022-02250-5 36136236

[391] VanDusseldorp TA, Escobar KA, Johnson KE, et al. Impact of varying dosages of fish oil on recovery and soreness following eccentric exercise. Nutrients. 2020;12(8):2246. doi: 10.3390/nu12082246 32727162 PMC7468920

[392] DiLorenzo FM, Drager CJ, Rankin JW. Docosahexaenoic acid affects markers of inflammation and muscle damage after eccentric exercise. J Strength Conditioning Res. 2014;28(10):2768–2774. doi: 10.1519/JSC.0000000000000617 25029008

[393] El‐Nemr S, Ismail I, Ragab M. Chemical composition of juice and seeds of pomegranate fruit. Food/Nahrung. 1990;34(7):601–606. doi: 10.1002/food.19900340706

[394] Bibi J, Lei Y, Kotwica-Mojzych K, et al. The power of pomegranate as natural supplement remedy for sportsmen and athletes: a systematic review and meta-analysis. J Funct Foods. 2024;121:106453. doi: 10.1016/j.jff.2024.106453. 2024/10/01/.

[395] Ammar A, Bailey SJ, Chtourou H, et al. Effects of pomegranate supplementation on exercise performance and post-exercise recovery in healthy adults: a systematic review. Br J Nutr. 2018 Dec;120(11):1201–1216. doi: 10.1017/S0007114518002696 30350760

[396] Ammar A, Turki M, Chtourou H, et al. Pomegranate supplementation accelerates recovery of muscle damage and soreness and inflammatory markers after a weightlifting training session. PLoS One. 2016;11(10):e0160305. doi: 10.1371/journal.pone.0160305 27764091 PMC5072630

[397] Trombold JR, Reinfeld AS, Casler JR, et al. The effect of pomegranate juice supplementation on strength and soreness after eccentric exercise. J Strength Cond Res. 2011 Jul;25(7):1782–1788. doi: 10.1519/JSC.0b013e318220d992 21659887

[398] Mohd Daud SM, Sukri NM, Johari MH, et al. Pure juice supplementation: its effect on muscle recovery and sports performance. Malays J Med Sci. 2023 Feb;30(1):31–48. doi: 10.21315/mjms2023.30.1.4 36875192 PMC9984102

[399] Lamb KL, Ranchordas MK, Johnson E, et al. No effect of tart cherry juice or pomegranate juice on recovery from exercise-induced muscle damage in non-resistance trained men. Nutrients. 2019;11(7):1593. doi: 10.3390/nu11071593 31337122 PMC6683053

[400] Ammar A, Trabelsi K, Bailey SJ, et al. Effects of natural polyphenol-rich pomegranate juice supplementation on plasma ion and lipid profiles following resistance exercise: a placebo-controlled trial. Nutr Metabol. 2020;17(1):31. 2020/04/16. doi: 10.1186/s12986-020-00451-1 PMC7164179 32322289

[401] Crum EM, Che Muhamed AM, Barnes M, et al. The effect of acute pomegranate extract supplementation on oxygen uptake in highly-trained cyclists during high-intensity exercise in a high altitude environment. J Int Soc Sports Nutr. 2017;14:14. doi: 10.1186/s12970-017-0172-0 28572749 PMC5452353

[402] Trombold J, Reinfeld A, Casler J. The effect of pomegranate juice supplementation on strength and soreness after eccentric exercise. J Strength Condition Res/National Strength Condition Assoc. 2011;25:1782–1788. 07/01. doi: 10.1519/JSC.0b013e318220d992 21659887

[403] Roelofs EJ, Smith-Ryan AE, Trexler ET, et al. Effects of pomegranate extract on blood flow and vessel diameter after high-intensity exercise in young, healthy adults. Eur J Sport Sci. 2017;17(3):317–325. doi: 10.1080/17461391.2016.1230892 27644475 PMC5563971

[404] Wang M, Yu L. Emerging evidence of urolithin A in sports nutrition: bridging preclinical findings to athletic applications [Review]. Front Nutr. 2025;Volume 12:2025. 2025–May–16. doi: 10.3389/fnut.2025.1585922 PMC12122305 40453722

[405] Ryu D, Mouchiroud L, Andreux PA, et al. Urolithin A induces mitophagy and prolongs lifespan in *C. elegans* and increases muscle function in rodents. Nat Med. 2016;22(8):879–888. doi: 10.1038/nm.4132 27400265

[406] D'Amico D, Olmer M, Fouassier AM, et al. Urolithin A improves mitochondrial health, reduces cartilage degeneration, and alleviates pain in osteoarthritis. Aging Cell. 2022;21(8):e13662. doi: 10.1111/acel.13662 35778837 PMC9381911

[407] Girotra M, Chiang Y-H, Charmoy M, et al. Induction of mitochondrial recycling reverts age-associated decline of the hematopoietic and immune systems. Nat Aging. 2023;3(9):1057–1066. doi: 10.1038/s43587-023-00473-3 37653255

[408] Fang EF, Hou Y, Palikaras K, et al. Mitophagy inhibits amyloid-β and tau pathology and reverses cognitive deficits in models of Alzheimer’s disease. Nat Neurosci. 2019;22(3):401–412. doi: 10.1038/s41593-018-0332-9 30742114 PMC6693625

[409] Lee HJ, Jung YH, Choi GE, et al. Urolithin A suppresses high glucose-induced neuronal amyloidogenesis by modulating TGM2-dependent ER-mitochondria contacts and calcium homeostasis. Cell Death Differen. 2021;28(1):184–202. doi: 10.1038/s41418-020-0593-1 PMC7852667 32704090

[410] Singh A, D’Amico D, Andreux PA, et al. Urolithin A improves muscle strength, exercise performance, and biomarkers of mitochondrial health in a randomized trial in middle-aged adults. Cell Rep Med. 2022;3(5):100633. doi: 10.1016/j.xcrm.2022.100633 35584623 PMC9133463

[411] Whitfield J, McKay AKA, Tee N, et al. Evaluating the impact of urolithin A supplementation on running performance, recovery, and mitochondrial biomarkers in highly trained Male distance runners. Sports Med. 2025. 21. 10.1007/s40279-025-02292-5 PMC12628386 40839339

[412] Zhao H, Zhu H, Yun H, et al. Assessment of urolithin A effects on muscle endurance, strength, inflammation, oxidative stress, and protein metabolism in Male athletes with resistance training: an 8-week randomized, double-blind, placebo-controlled study. J Int Soc Sports Nutr. 2024;21(1):2419388. doi: 10.1080/15502783.2024.2419388 39487653 PMC11536656

[413] D'Andrea G. Pycnogenol: a blend of procyanidins with multifaceted therapeutic applications? Fitoterapia. 2010;81(7):724–736. 2010/10/01/. doi: 10.1016/j.fitote.2010.06.011 20598812

[414] Maritim A, Dene B, Sanders R, et al. Effects of pycnogenol treatment on oxidative stress in streptozotocin‐induced diabetic rats. J Biochem Mol Toxicol. 2003;17(3):193–199. doi: 10.1002/jbt.10078 12815616

[415] Fitzpatrick DF, Bing B, Rohdewald P. Endothelium-dependent vascular effects of pycnogenol. J Cardiovasc Pharmacol. 1998;32(4):509–515. doi: 10.1097/00005344-199810000-00001 9781917

[416] Sivoňová M, Waczulíková I, Kilanczyk E, et al. The effect of pycnogenol on the erythrocyte membrane fluidity. Gen Physiol Biophys. 2004;23:39–51. 15270128

[417] Kim YJ, Kang KS, Yokozawa T. The anti-melanogenic effect of pycnogenol by its anti-oxidative actions. Food Chem Toxicol. 2008;46(7):2466–2471. doi: 10.1016/j.fct.2008.04.002 18482785

[418] Pourmasoumi M, Hadi A, Mohammadi H, et al. Effect of pycnogenol supplementation on blood pressure: a systematic review and meta‐analysis of clinical trials. Phytother Res. 2020;34(1):67–76. doi: 10.1002/ptr.6515 31637782

[419] Zhang Z, Tong X, & Wei YL, et al. (2020) Effect of Pycnogenol Supplementation on Blood Pressure: A Systematic Review and Meta-analysis. (2251-6085 (Print)). PMC6077626 30087862

[420] Belcaro G, Cornelli U, Luzzi R, et al. Pycnogenol® supplementation improves health risk factors in subjects with metabolic syndrome. Phytother Res. 2013;27(10):1572–1578. doi: 10.1002/ptr.4883 23359520

[421] Nikpayam O, Rouhani MH, Pourmasoumi M, et al. The effect of pycnogenol supplementation on plasma C-reactive protein concentration: a systematic review and meta-analysis. Clin Nutr Res. 2018;7(2):117. doi: 10.7762/cnr.2018.7.2.117 29713620 PMC5921329

[422] Zibadi S, Rohdewald PJ, Park D, et al. Reduction of cardiovascular risk factors in subjects with type 2 diabetes by pycnogenol supplementation. Nutr Res. 2008;28(5):315–320. 2008/05/01/. doi: 10.1016/j.nutres.2008.03.003 19083426

[423] Hadi A, Pourmasoumi M, Mohammadi H, et al. The impact of pycnogenol supplementation on plasma lipids in humans: a systematic review and meta‐analysis of clinical trials. Phytother Res. 2019;33(2):276–287. doi: 10.1002/ptr.6234 30456865

[424] Farid R, Mirfeizi Z, Mirheidari M, et al. Pycnogenol supplementation reduces pain and stiffness and improves physical function in adults with knee osteoarthritis. Nutr Res. 2007;27(11):692–697. 2007/11/01/. doi: 10.1016/j.nutres.2007.09.007 20934601

[425] Bentley DJ, Dank S, Coupland R, et al. Acute antioxidant supplementation improves endurance performance in trained athletes. Res Sports Med. 2012;20(1):1–12. doi: 10.1080/15438627.2011.608050 22242733

[426] Hara T, Igawa T, Ishizaka M, et al. Effects of pycnogenol-containing supplement on professional cycling performance: a single-group pretest-posttest pilot study. J Phys Fitness Sports Med. 2021;10(4):219–224. doi: 10.7600/jpfsm.10.219

[427] Vinciguerra G, Belcaro G, & Feragalli B, et al. (2019) PycnoRacer®, a fitness drink including Pycnogenol®, improves recovery and training in the Cooper test. (1827-1898 (Electronic)). 10.23736/S0031-0808.19.03639-5 32043843

[428] Vinciguerra G, Belcaro GF, & Bonanni E, et al. (2019) Evaluation of the effects of supplementation with Pycnogenol® on fitness in normal subjects with the Army Physical Fitness Test and in performances of athletes in the 100-minute triathlon. (0022-4707 (Print)). 24247188

[429] Igawa T, Hara T, Ishizaka M, et al. Changes in muscle strength and endurance of professional cyclists due to PycnoRacerTM. J Phys Ther Sci. 2021;33(4):339–344. doi: 10.1589/jpts.33.339 33935358 PMC8079890

[430] Vinciguerra G, Belcaro G, Cesarone MR, et al. Cramps and muscular pain: prevention with Pycnogenol® in normal subjects, venous patients, athletes, claudicants and in diabetic microangiopathy. Angiology. 2006;57(3):331–339. doi: 10.1177/000331970605700309 16703193

[431] Hosoi M, Belcaro G, Saggino A, et al. Pycnogenol® supplementation in minimal cognitive dysfunction. J Neurosurg Sci. 2018;62(3):279–284. doi: 10.23736/S0390-5616.18.04382-5 29754480

[432] Belcaro G, Cesarone MR, Hu S, et al. Pycnogenol® supplementation alleviates symptoms of Parkinson's disease with mild cognitive impairment. J Neurosurg Sci. 2022;66(4):371–377. doi: 10.23736/S0390-5616.22.05715-0 36153882

[433] Belcaro G, Dugall M, Hosoi M, et al. Pycnogenol® improves cognitive function in post-stroke patients: a 6 month-study. J Neurosurg Sci. 2024;68(1):109–116. doi: 10.23736/S0390-5616.22.05855-6 38299491

[434] Luzzi R, Belcaro G, Zulli C, et al. Pycnogenol® supplementation improves cognitive function, attention and mental performance in students. Panminerva Med. 2011;53(3 Suppl 1):75–82. 22108481

[435] Belcaro G, Hu S, Hosoi M, et al. Prevention and control of jet lag symptoms and temporary impairment of cognitive function with Pycnogenol® in healthy individuals and in hypertensives. Minerva Med. 2024;115. doi: 10.23736/S0026-4806.23.08974-7 38197571

[436] Ryan J, Croft K, Mori T, et al. An examination of the effects of the antioxidant Pycnogenol® on cognitive performance, serum lipid profile, endocrinological and oxidative stress biomarkers in an elderly population. J Psychopharmacol. 2008;22(5):553–562. doi: 10.1177/0269881108091584 18701642

[437] Belcaro G, Dugall M, Ippolito E, et al. The COFU3 study. Improvement in cognitive function, attention, mental performance with Pycnogenol® in healthy subjects (55-70) with high oxidative stress. J Neurosurg Sci. 2015;59(4):437–446. 26635191

[438] Belcaro G, Luzzi R, Dugall M, et al. Pycnogenol® improves cognitive function, attention, mental performance and specific professional skills in healthy professionals aged 35-55. J Neurosurg Sci. 2014;58(4):239–248. 24675223

[439] Aghababaei F, Hadidi M. Recent advances in potential health benefits of quercetin. Pharmaceuticals (Basel). 2023 Jul 18;16(7):1020. doi: 10.3390/ph16071020 37513932 PMC10384403

[440] Vollmannová A, Bojňanská T, Musilová J, et al. Quercetin as one of the most abundant represented biological valuable plant components with remarkable chemoprotective effects – a review. Heliyon. 2024;10(12):e33342. 2024/06/30/. doi: 10.1016/j.heliyon.2024.e33342 39021910 PMC11253541

[441] Chen S, Jiang H, Wu X, et al. Therapeutic effects of quercetin on inflammation, obesity, and type 2 diabetes. Mediat Inflamm. 2016;2016:9340637. doi: 10.1155/2016/9340637 PMC5149671 28003714

[442] Li Y, Yao J, Han C, et al. Quercetin, inflammation and immunity. Nutrients. 2016 Mar 15;8(3):167. doi: 10.3390/nu8030167 26999194 PMC4808895

[443] Wang Y, Li Z, He J, et al. Quercetin regulates lipid metabolism and fat accumulation by regulating inflammatory responses and glycometabolism pathways: a review. Nutrients. 2024 Apr 9;16(8):1102. doi: 10.3390/nu16081102 38674793 PMC11053503

[444] Carrillo-Martinez EJ, Flores-Hernández FY, Salazar-Montes AM, et al. Quercetin, a flavonoid with great pharmacological capacity. Molecules. 2024 Feb 25;29(5):1000. doi: 10.3390/molecules29051000 38474512 PMC10935205

[445] Hsu MY, Hsiao YP, Lin YT, et al. Quercetin alleviates the accumulation of superoxide in sodium iodate-induced retinal autophagy by regulating mitochondrial reactive oxygen species homeostasis through enhanced Deacetyl-SOD2 via the Nrf2-PGC-1α-Sirt1 pathway. Antioxidants (Basel). 2021 Jul 14;10(7):1125. doi: 10.3390/antiox10071125 34356358 PMC8301007

[446] Xie J, Song W, Liang X, et al. Protective effect of quercetin on streptozotocin-induced diabetic peripheral neuropathy rats through modulating gut microbiota and reactive oxygen species level. Biomed Pharmacother. 2020 Jul;127:110147. doi: 10.1016/j.biopha.2020.110147 32559841

[447] Alharbi HOA, Alshebremi M, Babiker AY, et al. The role of quercetin, a flavonoid in the management of pathogenesis through regulation of oxidative stress, inflammation, and biological activities. Biomolecules. 2025 Jan 20;15(1):151. doi: 10.3390/biom15010151 39858545 PMC11763763

[448] Kurtz JA, Grazer J, Wilson K, et al. The effect of quercetin and citrulline on cycling time trial performance. J Int Soc Sports Nutr. 2024 Dec;21(1):2416909. doi: 10.1080/15502783.2024.2416909 39417670 PMC11488173

[449] MacRae HS, Mefferd KM. Dietary antioxidant supplementation combined with quercetin improves cycling time trial performance. Int J Sport Nutr Exerc Metab. 2006 Aug;16(4):405–419. doi: 10.1123/ijsnem.16.4.405 17136942

[450] Kurtz JA, Vandusseldorp TA, Uken B, et al. Quercetin in sports and exercise: a review. Int J Exerc Sci. 2023;16(2):1334–1384. 38288402 10.70252/GQOK2958 PMC10824311

[451] Hai Y, Zhang Y, Liang Y, et al. Advance on the absorption, metabolism, and efficacy exertion of quercetin and its important derivatives. Food Frontiers. 2020;1(4):420–434. doi: 10.1002/fft2.50

[452] Brown K, Theofanous D, Britton RG, et al. Resveratrol for the management of human health: how far have we come? A systematic review of resveratrol clinical trials to highlight gaps and opportunities. Int J Mol Sci. 2024 Jan 6;25(2):747. doi: 10.3390/ijms25020747 38255828 PMC10815776

[453] Ruggiero M, Motti ML, Meccariello R, et al. Resveratrol and physical activity: a successful combination for the maintenance of health and wellbeing? Nutrients. 2025 Feb 28;17(5):837. doi: 10.3390/nu17050837 40077707 PMC11902109

[454] Bonnefont-Rousselot D. Resveratrol and cardiovascular diseases. Nutrients. 2016;8(5):250. doi: 10.3390/nu8050250 27144581 PMC4882663

[455] Kulkarni SS, Cantó C. The molecular targets of resveratrol. Biochim Biophys Acta. 2015 Jun;1852(6):1114–1123. doi: 10.1016/j.bbadis.2014.10.005 25315298

[456] Dolinsky VW, Jones KE, Sidhu RS, et al. Improvements in skeletal muscle strength and cardiac function induced by resveratrol during exercise training contribute to enhanced exercise performance in rats. J Physiol. 2012 Jun 1;590(11):2783–2799. doi: 10.1113/jphysiol.2012.230490 22473781 PMC3424731

[457] Hart N, Sarga L, Csende Z, et al. Resveratrol enhances exercise training responses in rats selectively bred for high running performance. Food Chem Toxicol. 2013 Nov;61:53–59. doi: 10.1016/j.fct.2013.01.051 23422033 PMC3703486

[458] Rodríguez-Bies E, Tung BT, Navas P, et al. Resveratrol primes the effects of physical activity in old mice. Br J Nutr. 2016 Sep;116(6):979–988. doi: 10.1017/S0007114516002920 27488121

[459] Gliemann L, Schmidt JF, Olesen J, et al. Resveratrol blunts the positive effects of exercise training on cardiovascular health in aged men. J Physiol. 2013 Oct 15;591(20):5047–5059. doi: 10.1113/jphysiol.2013.258061 23878368 PMC3810808

[460] Olesen J, Gliemann L, Biensø R, et al. Exercise training, but not resveratrol, improves metabolic and inflammatory status in skeletal muscle of aged men. J Physiol. 2014 Apr 15;592(8):1873–1886. doi: 10.1113/jphysiol.2013.270256 24514907 PMC4001758

[461] Scribbans TD, Ma JK, Edgett BA, et al. Resveratrol supplementation does not augment performance adaptations or fibre-type-specific responses to high-intensity interval training in humans. Appl Physiol Nutr Metab. 2014 Nov;39(11):1305–1313. doi: 10.1139/apnm-2014-0070 25211703

[462] Tsao JP, Liu CC, Wang HF, et al. Oral resveratrol supplementation attenuates exercise-induced Interleukin-6 but not oxidative stress after a high intensity cycling challenge in adults. Int J Med Sci. 2021;18(10):2137–2145. doi: 10.7150/ijms.55633 33859520 PMC8040419

[463] Anton SD, Embry C, Marsiske M, et al. Safety and metabolic outcomes of resveratrol supplementation in older adults: results of a twelve-week, placebo-controlled pilot study. Exp Gerontol. 2014 Sep;57:181–187. doi: 10.1016/j.exger.2014.05.015 24866496 PMC4149922

[464] Alcântara DB, Dionísio AP, Artur AG, et al. Selenium in Brazil nuts: an overview of agronomical aspects, recent trends in analytical chemistry, and health outcomes. Food Chem. 2022 Mar 15;372:131207. doi: 10.1016/j.foodchem.2021.131207 34634585

[465] Hu W, Zhao C, Hu H, et al. Food sources of selenium and its relationship with chronic diseases. Nutrients. 2021 May 20;13(5):1739. doi: 10.3390/nu13051739 34065478 PMC8160805

[466] Rayman MP. Selenium and human health. Lancet. 2012 Mar 31;379(9822):1256–1268. doi: 10.1016/S0140-6736(11)61452-9 22381456

[467] Tessier F, Margaritis I, Richard M-J, et al. Selenium and training effects on the glutathione system and aerobic performance. Med Sci Sports Exercise. 1995;27(3):390–396. 7752866

[468] Keane K (2014) Impact of high intensity interval training (HIIT) and/or selenium (Se) supplementation on oxidative stress and antioxidant status in active females.

[469] Hariri M, Amirkalali B, Baradaran HR, et al. The effect of parenteral selenium therapy on serum concentration of inflammatory mediators: a systematic review and dose-response meta-analysis of randomized clinical trials. Biol Trace Elem Res. 2024 May;202(5):1910–1925. doi: 10.1007/s12011-023-03806-w 37606878

[470] Kornyakova V, Badtieva V, Stepanova I, et al. Selenium as a factor for maintaining physical performance in elite athletes. Человек Спорт Медицина. 2023;23(2):55–60.

[471] Irawan R, Martiana T, Mahmudiono T, et al. Attenuating plasma cytokines response after high-intensity exercise through selenium supplementation. Int J Disabilities Sports Health Sci. 2025;8(2):143–151. doi: 10.33438/ijdshs.1552674

[472] Fernández-Lázaro D, Fernandez-Lazaro CI, Mielgo-Ayuso J, et al. The role of selenium mineral trace element in exercise: antioxidant defense system, muscle performance, hormone response, and athletic performance. A systematic review. Nutrients. 2020;12(6):1790. doi: 10.3390/nu12061790 32560188 PMC7353379

[473] Gogna S, Kaur J, Sharma K, et al. Spirulina- an edible cyanobacterium with potential therapeutic health benefits and toxicological consequences. J Am Nutr Assoc. 2023;42(6):559–572. 2023/08/18. doi: 10.1080/27697061.2022.2103852 35916491

[474] Gurney T, Spendiff O. Algae supplementation for exercise performance: current perspectives and future directions for spirulina and chlorella. Front Nutr. 2022;9:865741. doi: 10.3389/fnut.2022.865741 35321288 PMC8937016

[475] Koite NLN, Sanogo NI, Lépine O, et al. Antioxidant efficacy of a spirulina liquid extract on oxidative stress status and metabolic disturbances in subjects with metabolic syndrome. Mar Drugs. 2022 Jul 1;20(7):441. doi: 10.3390/md20070441 35877734 PMC9318250

[476] Bohórquez-Medina SL, Bohórquez-Medina AL, Benites Zapata VA, et al. Impact of spirulina supplementation on obesity-related metabolic disorders: a systematic review and meta-analysis of randomized controlled trials. NFS J. 2021;25(11/01):21–30. doi: 10.1016/j.nfs.2021.09.003

[477] Ismail M, Hossain MF, Tanu AR, et al. Effect of spirulina intervention on oxidative stress, antioxidant status, and lipid profile in chronic obstructive pulmonary disease patients. BioMed Res Int. 2015;2015:486120. doi: 10.1155/2015/486120 25685791 PMC4320919

[478] Krokidas A, Gakis AG, Aktypi O, et al. Effect of spirulina Nigrita(®) supplementation on indices of exercise-induced muscle damage after eccentric protocol of upper limbs in apparently healthy volunteers. Nutrients. 2024 May 28;16(11):1651. doi: 10.3390/nu16111651 38892584 PMC11174877

[479] Egner PA, Chen JG, Wang JB, et al. Bioavailability of sulforaphane from two broccoli sprout beverages: results of a short-term, cross-over clinical trial in qidong, China. Cancer Prev Res. 2011;4(3):384–395. doi: 10.1158/1940-6207.CAPR-10-0296 PMC3076202 21372038

[480] Fahey JW, Holtzclaw WD, Wehage SL, et al. Sulforaphane bioavailability from glucoraphanin-rich broccoli: control by active endogenous myrosinase. PLoS One. 2015;10(11):e0140963. doi: 10.1371/journal.pone.0140963 26524341 PMC4629881

[481] Fahey JW, Zhang Y, Talalay P. Broccoli sprouts: an exceptionally rich source of inducers of enzymes that protect against chemical carcinogens. Proc Natl Acad Sci. 1997;94(19):10367–10372. doi: 10.1073/pnas.94.19.10367 9294217 PMC23369

[482] Myzak MC, Dashwood RH. Chemoprotection by sulforaphane: keep one eye beyond Keap1. Cancer Lett. 2006;233(2):208–218. doi: 10.1016/j.canlet.2005.02.033 16520150 PMC2276573

[483] Zhang Y, Gordon GB. A strategy for cancer prevention: stimulation of the Nrf2-ARE signaling pathway. Mol Cancer Ther. 2004 Jul;3(7):885–893. doi: 10.1158/1535-7163.885.3.7 15252150

[484] Vermeulen M, Klöpping-Ketelaars IW, van den Berg R, et al. Bioavailability and kinetics of sulforaphane in humans after consumption of cooked versus raw broccoli. J Agric Food Chem. 2008;56(22):10505–10509. doi: 10.1021/jf801989e 18950181

[485] Yagishita Y, Fahey JW, Dinkova-Kostova AT, et al. Broccoli or sulforaphane: is it the source or dose that matters? Molecules. 2019;24(19):3593. doi: 10.3390/molecules24193593 31590459 PMC6804255

[486] Kensler TW, Wakabayashi N, Biswal S. Cell survival responses to environmental stresses via the Keap1-Nrf2-ARE pathway. Annu Rev Pharmacol Toxicol. 2007;47(1):89–116. doi: 10.1146/annurev.pharmtox.46.120604.141046 16968214

[487] Li W, Kong AN. Molecular mechanisms of Nrf2-mediated antioxidant response. Mol Carcinog. 2009;48(2):91–104. Published in cooperation with the University of Texas MD Anderson Cancer Center. doi: 10.1002/mc.20465 18618599 PMC2631094

[488] Paulsen G, Cumming KT, Holden G, et al. Vitamin C and E supplementation hampers cellular adaptation to endurance training in humans: a double‐blind, randomised, controlled trial. J Physiol. 2014;592(8):1887–1901. doi: 10.1113/jphysiol.2013.267419 24492839 PMC4001759

[489] Guerrero-Beltrán CE, Calderón-Oliver M, Pedraza-Chaverri J, et al. Protective effect of sulforaphane against oxidative stress: recent advances. Exp Toxicol Pathol. 2012;64(5):503–508. doi: 10.1016/j.etp.2010.11.005 21129940

[490] Heiss E, Herhaus C, Klimo K, et al. Nuclear factor κB is a molecular target for sulforaphane-mediated anti-inflammatory mechanisms. J Biol Chem. 2001;276(34):32008–32015. doi: 10.1074/jbc.M104794200 11410599

[491] Treasure K, Harris J, Williamson G. Exploring the anti‐inflammatory activity of sulforaphane. Immunol Cell Biol. 2023;101(9):805–828. doi: 10.1111/imcb.12686 37650498

[492] Ruhee RT, Suzuki K. The integrative role of sulforaphane in preventing inflammation, oxidative stress and fatigue: a review of a potential protective phytochemical. Antioxidants. 2020;9(6):521. doi: 10.3390/antiox9060521 32545803 PMC7346151

[493] Ruhee RT, Suzuki K. The immunomodulatory effects of sulforaphane in exercise-induced inflammation and oxidative stress: a prospective nutraceutical. Int J Mol Sci. 2024;25(3):1790. doi: 10.3390/ijms25031790 38339067 PMC10855658

[494] Sato K, Kihara H, Kumazawa Y, et al. Oral chronic sulforaphane effects on heavy resistance exercise: implications for inflammatory and muscle damage parameters in young practitioners. Nutrition. 2021;90:111266. doi: 10.1016/j.nut.2021.111266 34004418

[495] Armah CN, Derdemezis C, Traka MH, et al. Diet rich in high glucoraphanin broccoli reduces plasma LDL cholesterol: evidence from randomised controlled trials. Mol Nutr Food Res. 2015;59(5):918–926. doi: 10.1002/mnfr.201400863 25851421 PMC4692095

[496] López-Chillón MT, Carazo-Díaz C, Prieto-Merino D, et al. Effects of long-term consumption of broccoli sprouts on inflammatory markers in overweight subjects. Clin Nutr. 2019;38(2):745–752. doi: 10.1016/j.clnu.2018.03.006 29573889

[497] Komine S, Miura I, Miyashita N, et al. Effect of a sulforaphane supplement on muscle soreness and damage induced by eccentric exercise in young adults: a pilot study. Physiol Rep. 2021 Dec;9(24):e15130. doi: 10.14814/phy2.15130 34927380 PMC8685487

[498] Colletti A, Cravotto G, De Meo A, et al. Health benefits of (Poly) phenols from cherries: a review of clinical trials. Nutraceuticals. 2025;5(2):12. doi: 10.3390/nutraceuticals5020012

[499] Kelley DS, Adkins Y, Laugero KD. A review of the health benefits of cherries. Nutrients. 2018;10(3):368. doi: 10.3390/nu10030368 29562604 PMC5872786

[500] Alba CM-A, Daya M, Franck C. Tart cherries and health: current knowledge and need for a better understanding of the fate of phytochemicals in the human gastrointestinal tract. Crit Rev Food Sci Nutr. 2019;59(4):626–638. doi: 10.1080/10408398.2017.1384918 28956621

[501] Bell P, McHugh M, Stevenson E, et al. The role of cherries in exercise and health. Scand J Med Sci Sports. 2014;24(3):477–490. doi: 10.1111/sms.12085 23710994

[502] Hill JA, Keane KM, Quinlan R, et al. Tart cherry supplementation and recovery from strenuous exercise: a systematic review and meta-analysis. Int J Sport Nutr Exerc Metab. 2021;31(2):154–167. doi: 10.1123/ijsnem.2020-0145 33440334

[503] Hooper DR, Orange T, Gruber MT, et al. Broad spectrum polyphenol supplementation from tart cherry extract on markers of recovery from intense resistance exercise. J Int Soc Sports Nutr. 2021 Jun 14;18(1):47. doi: 10.1186/s12970-021-00449-x 34126996 PMC8204440

[504] Levers K, Dalton R, Galvan E, et al. Effects of powdered montmorency tart cherry supplementation on an acute bout of intense lower body strength exercise in resistance trained males. J Int Soc Sports Nutr. 2015;12(1):41. doi: 10.1186/s12970-015-0102-y 26578852 PMC4647629

[505] Bowtell J, Sumners D, Dyer A, et al. Montmorency cherry juice reduces muscle damage caused by intensive strength exercise. Med Sc Sports Exerc. 2011;43(8):1544–1551. 21233776 10.1249/MSS.0b013e31820e5adc

[506] Ortega DG, Coburn JW, Galpin AJ, et al. Effects of a tart cherry supplement on recovery from exhaustive exercise. J Funct Morphol Kinesiol. 2023 Aug 18;8(3):121. doi: 10.3390/jfmk8030121 37606416 PMC10443385

[507] Jackman SR, Brook MS, Pulsford RM, et al. Tart cherry concentrate does not enhance muscle protein synthesis response to exercise and protein in healthy older men. Exp Gerontol. 2018;110:202–208. doi: 10.1016/j.exger.2018.06.007 29890270

[508] Connolly D, McHugh M, Padilla-Zakour O. Efficacy of a tart cherry juice blend in preventing the symptoms of muscle damage. Br J Sports Med. 2006;40(8):679–683. doi: 10.1136/bjsm.2005.025429 16790484 PMC2579450

[509] Squires E, Walshe IH, Dodd A, et al. Acute dosing strategy with Vistula tart cherries for recovery of strenuous Exercise—A feasibility study. Nutrients. 2024;16(16):2709. doi: 10.3390/nu16162709 39203845 PMC11357489

[510] Howatson G, McHugh MP, Hill J, et al. Influence of tart cherry juice on indices of recovery following marathon running. Scand J Med Sci Sports. 2010;20(6):843–852. doi: 10.1111/j.1600-0838.2009.01005.x 19883392

[511] Kuehl KS, Perrier ET, Elliot DL, et al. Efficacy of tart cherry juice in reducing muscle pain during running: a randomized controlled trial. J Int Soc Sports Nutr. 2010;7:1–6. doi: 10.1186/1550-2783-7-17 20459662 PMC2874510

[512] Levers K, Dalton R, Galvan E, et al. Effects of powdered montmorency tart cherry supplementation on acute endurance exercise performance in aerobically trained individuals. J Int Soc Sports Nutr. 2016;13(1):22. doi: 10.1186/s12970-016-0133-z 27231439 PMC4880859

[513] Bell PG, Stevenson E, Davison GW, et al. The effects of montmorency tart cherry concentrate supplementation on recovery following prolonged, intermittent exercise. Nutrients. 2016;8(7):441. doi: 10.3390/nu8070441 27455316 PMC4963917

[514] Bell PG, Walshe IH, Davison GW, et al. Recovery facilitation with montmorency cherries following high-intensity, metabolically challenging exercise. Appl Physiol Nutr Metab. 2015 Apr;40(4):414–423. doi: 10.1139/apnm-2014-0244 25794236

[515] McCormick R, Peeling P, Binnie M, et al. Effect of tart cherry juice on recovery and next day performance in well-trained water polo players. J Int Soc Sports Nutr. 2016;13:41. doi: 10.1186/s12970-016-0151-x 27895542 PMC5109721

[516] Gao R, Chilibeck PD. Effect of tart cherry concentrate on endurance exercise performance: a meta-analysis. J Am Coll Nutr. 2020;39(7):657–664. doi: 10.1080/07315724.2020.1713246 31986108

[517] Wangdi JT, O’Leary MF, Kelly VG, et al. Montmorency cherry supplementation enhances 15 km cycling time trial performance: optimal timing 90‐min pre‐exercise. Eur J Sport Sci. 2024;24(10):1480–1494. doi: 10.1002/ejsc.12187 39213288 PMC11451560

[518] McHugh MP. “Precovery” versus recovery: understanding the role of cherry juice in exercise recovery. Scand J Med Sci Sports. 2022;32(6):940–950. 35119142 10.1111/sms.14141 PMC9306613

[519] Bell PG, Walshe IH, Davison GW, et al. Montmorency cherries reduce the oxidative stress and inflammatory responses to repeated days high-intensity stochastic cycling. Nutrients. 2014;6(2):829–843. doi: 10.3390/nu6020829 24566440 PMC3942735

[520] Bell PG, Walshe IH, Davison GW, et al. Recovery facilitation with montmorency cherries following high-intensity, metabolically challenging exercise. Appl Physiol Nutr Metabol. 2015;40(4):414–423. doi: 10.1139/apnm-2014-0244 25794236

[521] Brown MA, Stevenson EJ, Howatson G. Montmorency tart cherry (Prunus cerasus L.) supplementation accelerates recovery from exercise-induced muscle damage in females. Eur J Sport Sci. 2019;19(1):95–102. doi: 10.1080/17461391.2018.1502360 30058460

[522] Quinlan R, Hill JA. The efficacy of tart cherry juice in aiding recovery after intermittent exercise. Int J Sports Physiol Perform. 2020;15(3):368–374. doi: 10.1123/ijspp.2019-0101 31614329

[523] Stretton B, Eranki A, Kovoor J, et al. Too sour to be true? Tart cherries (*Prunus cerasus*) and sleep: a systematic review and meta-analysis. Curr Sleep Med Rep. 2023;9(3):225–233. doi: 10.1007/s40675-023-00261-w

[524] Chai SC, Davis K, Zhang Z, et al. Effects of tart cherry juice on biomarkers of inflammation and oxidative stress in older adults. Nutrients. 2019 Jan 22;11(2):228. doi: 10.3390/nu11020228 30678193 PMC6413159

[525] Moosavian SP, Maharat M, Chambari M, et al. Effects of tart cherry juice consumption on cardio-metabolic risk factors: a systematic review and meta-analysis of randomized-controlled trials. Complement Ther Med. 2022;71:102883. 2022/12/01/. doi: 10.1016/j.ctim.2022.102883 36038032

[526] Constantinescu A, Han D, Packer L. Vitamin E recycling in human erythrocyte membranes. J Biol Chem. 1993 May 25;268(15):10906–10913. 8388377

[527] Wardenaar F, Brinkmans N, Ceelen I, et al. Micronutrient intakes in 553 Dutch elite and sub-elite athletes: prevalence of low and high intakes in users and non-users of nutritional supplements. Nutrients. 2017;9(2):142. doi: 10.3390/nu9020142 28212284 PMC5331573

[528] Ristow M, Zarse K, Oberbach A, et al. Antioxidants prevent health-promoting effects of physical exercise in humans. Proc Natl Acad Sci U S A. 2009 May 26;106(21):8665–8670. doi: 10.1073/pnas.0903485106 19433800 PMC2680430

[529] Akova B, Sürmen-Gür E, Gür H, et al. Exercise-induced oxidative stress and muscle performance in healthy women: role of vitamin E supplementation and endogenous oestradiol. Eur J Appl Physiol. 2001 Jan;84(1-2):141–147. doi: 10.1007/s004210000331 11394244

[530] Bjørnsen T, Salvesen S, Berntsen S, et al. Vitamin C and E supplementation blunts increases in total lean body mass in elderly men after strength training. Scand J Med Sci Sports. 2016 Jul;26(7):755–763. doi: 10.1111/sms.12506 26129928

[531] Bobeuf F, Labonte M, Dionne IJ, et al. Combined effect of antioxidant supplementation and resistance training on oxidative stress markers, muscle and body composition in an elderly population. J Nutr Health Aging. 2011 Dec;15(10):883–889. doi: 10.1007/s12603-011-0097-2 22159777 PMC12878122

[532] Chou CC, Sung YC, Davison G, et al. Short-term high-dose vitamin C and E supplementation attenuates muscle damage and inflammatory responses to repeated taekwondo competitions: a randomized placebo-controlled trial. Int J Med Sci. 2018;15(11):1217–1226. doi: 10.7150/ijms.26340 30123060 PMC6097262

[533] Cumming KT, Raastad T, Sørstrøm A, et al. Vitamin C and E supplementation does not affect heat shock proteins or endogenous antioxidants in trained skeletal muscles during 12 weeks of strength training. BMC Nutr. 2017;3:70. doi: 10.1186/s40795-017-0185-8 32153849 PMC7050865

[534] de Oliveira DCX, Rosa FT, Simões-Ambrósio L, et al. Antioxidant vitamin supplementation prevents oxidative stress but does not enhance performance in young football athletes. Nutrition. 2019 Jul–Aug;63-64:29–35. doi: 10.1016/j.nut.2019.01.007 30927644

[535] He F, Hockemeyer JA, Sedlock D. Does combined antioxidant vitamin supplementation blunt repeated bout effect? Int J Sports Med. 2015 May;36(5):407–413. doi: 10.1055/s-0034-1395630 25607519

[536] Morrison D, Hughes J, Della Gatta PA, et al. Vitamin C and E supplementation prevents some of the cellular adaptations to endurance-training in humans. Free Radic Biol Med. 2015 Dec;89:852–862. doi: 10.1016/j.freeradbiomed.2015.10.412 26482865

[537] Nalbant O, Toktaş N, Toraman NF, et al. Vitamin E and aerobic exercise: effects on physical performance in older adults. Aging Clin Exp Res. 2009 Apr;21(2):111–121. doi: 10.1007/BF03325218 19448382

[538] Paulsen G, Hamarsland H, Cumming KT, et al. Vitamin C and E supplementation alters protein signalling after a strength training session, but not muscle growth during 10 weeks of training. J Physiol. 2014 Dec 15;592(24):5391–5408. doi: 10.1113/jphysiol.2014.279950 25384788 PMC4270502

[539] Shafat A, Butler P, Jensen R, et al. Effects of dietary supplementation with vitamins C and E on muscle function during and after eccentric contractions in humans. Eur J Appl Physiol. 2004;93:196–202. doi: 10.1007/s00421-004-1198-y 15309547

[540] Theodorou AA, Nikolaidis MG, Paschalis V, et al. No effect of antioxidant supplementation on muscle performance and blood redox status adaptations to eccentric training. AJCN. 2011 Jun;93(6):1373–1383. doi: 10.3945/ajcn.110.009266 21508092

[541] Wyckelsma VL, Venckunas T, Brazaitis M, et al. Vitamin C and E treatment blunts sprint interval training-induced changes in inflammatory Mediator-, Calcium-, and mitochondria-related signaling in recreationally active elderly humans. Antioxidants (Basel). 2020 Sep 17;9(9):879. doi: 10.3390/antiox9090879 32957522 PMC7555371

[542] Yfanti C, Akerström T, Nielsen S, et al. Antioxidant supplementation does not alter endurance training adaptation. Med Sci Sports Exerc. 2010 Jul;42(7):1388–1395. doi: 10.1249/MSS.0b013e3181cd76be 20019626

[543] Yfanti C, Fischer CP, Nielsen S, et al. Role of vitamin C and E supplementation on IL-6 in response to training. J Appl Physiol (1985). 2012 Mar;112(6):990–1000. doi: 10.1152/japplphysiol.01027.2010 22207723

[544] Yfanti C, Nielsen AR, Akerström T, et al. Effect of antioxidant supplementation on insulin sensitivity in response to endurance exercise training. Am J Physiol Endocrinol Metab. 2011 May;300(5):E761–70. doi: 10.1152/ajpendo.00207.2010 21325105

[545] Yfanti C, Tsiokanos A, Fatouros IG, et al. Chronic eccentric exercise and antioxidant supplementation: effects on lipid profile and insulin sensitivity. J Sports Sci Med. 2017 Sep;16(3):375–382. 28912655 PMC5592289

[546] Zoppi CC, Hohl R, Silva FC, et al. Vitamin C and e supplementation effects in professional soccer players under regular training. J Int Soc Sports Nutr. 2006 Dec 13;3(2):37–44. doi: 10.1186/1550-2783-3-2-37 18500971 PMC2129167

[547] Santos S, Silva E, Caris A, et al. Vitamin E supplementation inhibits muscle damage and inflammation after moderate exercise in hypoxia. J Hum Nutr Diet. 2016;29(4):516–522. doi: 10.1111/jhn.12361 27062041

[548] Bryer S, Goldfarb AH. Effect of high dose vitamin C supplementation on muscle soreness, damage, function, and oxidative stress to eccentric exercise. Int J Sport Nutr Exerc Metab. 2006;16(3):270–280. doi: 10.1123/ijsnem.16.3.270 16948483

[549] Connolly D, Lauzon C, Agnew J, et al. The effects of vitamin C supplementation on symptoms of delayed onset muscle soreness. J Sports Med Phys Fitness. 2006;46(3):462. 16998453

[550] Evans LW, Zhang F, Omaye ST. Vitamin C supplementation reduces exercise-induced oxidative stress and increases peak muscular force. Food Nutr Sci. 2017;8(8):812–822. doi: 10.4236/fns.2017.88058

[551] Gomez-Cabrera M-C, Domenech E, Romagnoli M, et al. Oral administration of vitamin C decreases muscle mitochondrial biogenesis and hampers training-induced adaptations in endurance performance. Am J Clin Nutr. 2008;87(1):142–149. doi: 10.1093/ajcn/87.1.142 18175748

[552] Paschalis V, Theodorou AA, Kyparos A, et al. Low vitamin C values are linked with decreased physical performance and increased oxidative stress: reversal by vitamin C supplementation. Eur J Nutr. 2016 Feb;55(1):45–53. doi: 10.1007/s00394-014-0821-x 25526969

[553] Roberts LA, Beattie K, Close GL, et al. Vitamin C consumption does not impair training-induced improvements in exercise performance. Int J Sports Physiol Perform. 2011;6(1):58–69. doi: 10.1123/ijspp.6.1.58 21487150

[554] Scalzo RL, Bauer TA, Harrall K, et al. Acute vitamin C improves cardiac function, not exercise capacity, in adults with type 2 diabetes. Diabetol Metabol Syndrome. 2018;10:1–9. doi: 10.1186/s13098-018-0306-9 PMC5813393 29456629

[555] Thompson D, Williams C, McGregor SJ, et al. Prolonged vitamin C supplementation and recovery from demanding exercise. Int J Sport Nutr Exerc Metab. 2001;11(4):466–481. doi: 10.1123/ijsnem.11.4.466 11915781

[556] Close GL, Ashton T, Cable T, et al. Ascorbic acid supplementation does not attenuate post-exercise muscle soreness following muscle-damaging exercise but May delay the recovery process. Br J Nutr. 2006;95(5):976–981. doi: 10.1079/BJN20061732 16611389

[557] Nikolaidis MG, Kerksick CM, Lamprecht M, et al. Does vitamin C and E supplementation impair the favorable adaptations of regular exercise? Oxid Med Cell Longev. 2012;2012:707941. doi: 10.1155/2012/707941 22928084 PMC3425865

[558] Powers S, Sollanek K, Wiggs M. Endurance exercise and antioxidant supplementation: sense or nonsense. Part, 1:1–4.

[559] Powell SR. The antioxidant properties of zinc. J Nutr. 2000;130(5):1447S–1454S. doi: 10.1093/jn/130.5.1447S 10801958

[560] Ribeiro SM, Braga CB, Peria FM, et al. Effect of zinc supplementation on antioxidant defenses and oxidative stress markers in patients undergoing chemotherapy for colorectal cancer: a placebo-controlled, prospective randomized trial. Biol Trace Elem Res. 2016 Jan;169(1):8–16. doi: 10.1007/s12011-015-0396-2 26066525

[561] Oteiza PI. Zinc and the modulation of redox homeostasis. Free Radic Biol Med. 2012 Nov 1;53(9):1748–1759. doi: 10.1016/j.freeradbiomed.2012.08.568 22960578 PMC3506432

[562] Cousins RJ, Liuzzi JP, Lichten LA. Mammalian zinc transport, trafficking, and signals. J Biol Chem. 2006 Aug 25;281(34):24085–24089. doi: 10.1074/jbc.R600011200 16793761

[563] Mocchegiani E, Romeo J, Malavolta M, et al. Zinc: dietary intake and impact of supplementation on immune function in elderly. Age (Dordr). 2013 Jun;35(3):839–860. doi: 10.1007/s11357-011-9377-3 22222917 PMC3636409

[564] Kara E, Gunay M, Cicioglu İ, et al. Effect of zinc supplementation on antioxidant activity in young wrestlers. Biol Trace Elem Res. 2010;134(1):55–63. 2010/04/01. doi: 10.1007/s12011-009-8457-z 19597720

[565] Hosseini R, Ferns GA, Sahebkar A, et al. Zinc supplementation is associated with a reduction in serum markers of inflammation and oxidative stress in adults: a systematic review and meta-analysis of randomized controlled trials. Cytokine. 2021 Feb;138:155396. doi: 10.1016/j.cyto.2020.155396 33333394

[566] Padilha C, Ribeiro A, Fleck S, et al. Effect of resistance training with different frequencies and detraining on muscular strength and oxidative stress biomarkers in older women. Age Dordr Neth. 2015;37(5):104. doi: 10.1007/s11357-015-9841-6 PMC5005843 26423425

[567] Bouzid MA, Filaire E, McCall A, et al. Radical oxygen species, exercise and aging: an update. Sports Med. 2015;45:1245–1261. doi: 10.1007/s40279-015-0348-1 26119427

[568] Joseph AM, Adhihetty PJ, Leeuwenburgh C. Beneficial effects of exercise on age‐related mitochondrial dysfunction and oxidative stress in skeletal muscle. J Physiol. 2016;594(18):5105–5123. doi: 10.1113/JP270659 26503074 PMC5023701

[569] Vina J, Borras C, Abdelaziz KM, et al. The free radical theory of aging revisited: the cell signaling disruption theory of aging. Antioxid Redox Signaling. 2013;19(8):779–787. doi: 10.1089/ars.2012.5111 PMC3749699 23841595

[570] Harman D. Aging: a theory based on free radical and radiation chemistry. J Gerontol. 1956 Jul;11(3):298–300. doi: 10.1093/geronj/11.3.298 13332224

[571] Bua E, Johnson J, Herbst A, et al. Mitochondrial DNA–deletion mutations accumulate intracellularly to detrimental levels in aged human skeletal muscle fibers. Am J Hum Genet. 2006;79(3):469–480. doi: 10.1086/507132 16909385 PMC1559550

[572] Guo S, Shaoni GLL, Stuart-Smith WA, et al. Dietary intake of masters athletes: a systematic review. Nutrients. 2023 Nov 30;15(23):4973. doi: 10.3390/nu15234973 38068832 PMC10708321

[573] Huang CJ, Webb HE, Zourdos MC, et al. Cardiovascular reactivity, stress, and physical activity. Front Physiol. 2013 Nov 7;4:314. doi: 10.3389/fphys.2013.00314 24223557 PMC3819592

[574] Gonzalez DE, McAllister MJ, Waldman HS, et al. International society of sports nutrition position stand: tactical athlete nutrition. J Int Soc Sports Nutr. 2022;19(1):267–315. doi: 10.1080/15502783.2022.2086017 35813846 PMC9261739

[575] McAllister MJ, Basham SA, Smith JW, et al. Effects of environmental heat and antioxidant ingestion on blood markers of oxidative stress in professional firefighters performing structural fire exercises. J Occup Environ Med. 2018 Nov;60(11):e595–e601. doi: 10.1097/JOM.0000000000001452 30252723

[576] Miao X, Li B, Zhu Z, et al. Sex differences in the association between composite dietary antioxidant index and hyperlipidemia: insights from NHANES. PLoS One. 2025;20(1):e0316130. doi: 10.1371/journal.pone.0316130 39792922 PMC11723626

[577] Tiberi J, Cesarini V, Stefanelli R, et al. Sex differences in antioxidant defence and the regulation of redox homeostasis in physiology and pathology. Mech Ageing Dev. 2023;211:111802. 2023/04/01/. doi: 10.1016/j.mad.2023.111802 36958540

[578] Kander MC, Cui Y, Liu Z. Gender difference in oxidative stress: a new look at the mechanisms for cardiovascular diseases. J Cell Mol Med. 2017 May;21(5):1024–1032. 27957792 10.1111/jcmm.13038 PMC5387169

[579] Strehlow K, Rotter S, Wassmann S, et al. Modulation of antioxidant enzyme expression and function by estrogen. Circ Res. 2003 Jul 25;93(2):170–177. doi: 10.1161/01.RES.0000082334.17947.11 12816884

[580] Duan L, Zeng R, Wang J, et al. Gender difference in the association between composite dietary antioxidant index and all-cause mortality [Original Research]. Front Nutr. 2025;12:2025. 2025–March–04. doi: 10.3389/fnut.2025.1523171 PMC11913696 40104815

[581] Aryan L, Younessi D, Zargari M, et al. The role of estrogen receptors in cardiovascular disease. Int J Mol Sci. 2020;21(12):4314. doi: 10.3390/ijms21124314 32560398 PMC7352426

[582] Ide T, Tsutsui H, Ohashi N, et al. Greater oxidative stress in healthy young men compared with premenopausal women. Arterioscler Thromb Vasc Biol. 2002 Mar 1;22(3):438–442. doi: 10.1161/hq0302.104515 11884287

[583] Xiang D, Liu Y, Zhou S, et al. Protective effects of estrogen on cardiovascular disease mediated by oxidative stress. Oxid Med Cell Longev. 2021;2021(1):5523516. doi: 10.1155/2021/5523516 34257804 PMC8260319

[584] Sims ST, Kerksick CM, Smith-Ryan AE, et al. International society of sports nutrition position stand: nutritional concerns of the female athlete. J Int Soc Sports Nutr. 2023 Dec;20(1):2204066. doi: 10.1080/15502783.2023.2204066 37221858 PMC10210857

[585] Association IBI. 2024. Brain Injury Facts 2024 [cited 2024 June 1]. Available from: https://www.internationalbrain.org/resources/brain-injury-facts

[586] Conti F, McCue JJ, DiTuro P, et al. Mitigating traumatic brain injury: a narrative review of supplementation and dietary protocols. Nutrients. 2024 Jul 26;16(15):2430. doi: 10.3390/nu16152430 39125311 PMC11314487

[587] Excellence TBICo Annual Report. Washington, DC, USA: TBICoE; 2023.

[588] Matney C, Bowman K, Berwick D, et al. The scope and burden of traumatic brain injury. Traumatic Brain Injury: a roadmap for accelerating progress. US: National Academies Press; 2022. 35533242

[589] Daneshvar DH, Nowinski CJ, McKee AC, et al. The epidemiology of sport-related concussion. Clin Sports Med. 2011 Jan;30(1):1–17. vii. doi: 10.1016/j.csm.2010.08.006 21074078 PMC2987636

[590] Clark M, Guskiewicz K. 2016. Sport-related traumatic brain injury. Translational research in traumatic brain injury. 26583180

[591] Powell JW, Barber-foss KD. Traumatic brain injury in high school athletes. J Am Med Assoc. 1999 Sep 8;282(10):958–963. doi: 10.1001/jama.282.10.958 10485681

[592] Strack JE, Torres VA, Pennington ML, et al. Psychological distress and line-of-duty head injuries in firefighters. Occup Med (Chic Ill). 2021;71(2):99–104. doi: 10.1093/occmed/kqab013 33598694

